# *In situ* Chemical Profiling and Imaging of Cultured and Natural *Cordyceps sinensis* by TOF-SIMS

**DOI:** 10.3389/fchem.2022.862007

**Published:** 2022-03-24

**Authors:** Qian-Bao Liu, Jing-Guang Lu, Zhi-Hong Jiang, Wei Zhang, Wen-Jia Li, Zheng-Ming Qian, Li-Ping Bai

**Affiliations:** ^1^ State Key Laboratory of Quality Research in Chinese Medicine, Macau Institute for Applied Research in Medicine and Health, Macau University of Science and Technology, Taipa, Macau, China; ^2^ Guangdong-Hong Kong-Macao Joint Laboratory of Respiratory Infectious Disease, Macau University of Science and Technology, Taipa, Macau, China; ^3^ Dongguan HEC Cordyceps R and D Co., Ltd., Dongguan, China

**Keywords:** time-of-flight secondary ion mass spectrometry, Cordyceps sinensis, *in situ* analysis, chemical imaging, traditional Chinese medicines

## Abstract

Time-of-flight secondary ion mass spectrometry (TOF-SIMS) is a sensitive surface analytical technology, which can simultaneously acquire diverse chemical components and their precise locations on the surfaces of samples without any requirements for chemical damage pretreatments or additional matrices. Commonly, the quality control of TCMs (traditional Chinese medicines) is limited by the qualitative and quantitative evaluations of the specifically extractive constituents. In this study, a practical sample preparation strategy named two-layered media embedding sample preparation was developed to obtain ideal freezing sections of dried materials of *Cordyceps sinensis*. Meanwhile, the well-established sample preparation method was applied for *in situ* chemical profiling and imaging of natural (NCS) and cultured *Cordyceps sinensis* (CCS) by using TOF-SIMS. More than 200 components were tentatively identified and imaged in NCS and CCS at the same time. Mass spectrometry imaging revealed that most components have even distributions in caterpillars of *Cordyceps sinensis*, while TAGs, DAGs, MAGs, and FAs only have distributions outside caterpillars’ digestive chambers. This is the first time that components were *in situ* imaged for *Cordyceps sinensis* to exhibit the chemical distributions which have never been achieved by other analytical techniques so far. In addition, chemometrics was used to simplify and explain the massive TOF-SIMS mass data sets, which revealed the high chemical similarity between CCS and NCS. Furthermore, the relative quantification of TOF-SIMS data showed that CCS has comparable proportions of amino acids, nucleosides, monosaccharides, sphingolipids, sterols and other principles to NCS except for fatty acids, glycerides and glycerophospholipids. The higher amounts of TAGs and DAGs in CCS were confirmed by quantitative ^1^H-NMR, indicating reliable relative quantification of TOF-SIMS. In general, our research developed a novel approach of TOF-SIMS for *in situ* chemical analysis of TCMs, and its successful application in comparative study of CCS and NCS suggested that TOF-SIMS is an advanced and promising analytical technology for the research of TCMs.

## 1 Introduction

*Cordyceps sinensis* (*C. sinensis*) is a precious food and well-known traditional Chinese medicine (TCM). It has been used as a medicinal food in China over 300 years. Forming on an insect larva infected by the *C. sinensis* fungus, it is a complex comprising of the stromata of fungus and the dead caterpillar. Owing to the changeable appearance during its growth process, *C. sinensis* is named *Dong Chong Xia Cao* (winter worm summer grass) in China. In recent years, it has been demonstrated that *C. sinensis* was rich in many bioactive constituents including nucleosides, saccharides, sterols, fatty acids, proteins, cyclic dipeptides, amino acids, polyamines, volatile components, organic acids, vitamins, and mannitol (Zhao et al., 2014). Modern pharmacological studies showed that *C. sinensis* had multiple pharmacological functions, including antitumor, immunomodulating, anti-oxidative, and anti-inflammatory activities (Olatunji et al., 2018).

The Tibetan Plateau and its environs are home to *C. sinensis*, with the lowest height for its distribution being 3,000 m (Li et al., 2019). The yield of natural *C. sinensis* (NCS) is dropping every year, and it is on the verge of extinction, due to the unpredictability of infection, restricted habitat, and high medicinal needs. As a result, there was a severe shortage of supply and a sharp rise in prices. Furthermore, the local natural ecosystem has been severely affected due to the excessive collecting. With the unremitting efforts of researchers, the breeding industry of cultured *C. sinensis* (CCS) has matured. Currently, the cultivation of CCS is mainly divided into two ways. One way is semi-wild breeding, which involves artificially infecting cordyceps and then releasing them into the wild to flourish (Li et al., 2016). The yield and quality of semi-wild breeding are readily unstable, inconsistent and uncontrollable due to the numerous unknown aspects of natural circumstances. The alternative way is imitating ecological breeding which requires performing all breeding processes indoors (Li, et al., 2016). This method necessitates not only strict environmental parameters including temperature, humidity, soil, and so on, but also professional breeding skills. At present, the prices of NCS and CCS might differ by over 100 folds (Li, et al., 2019). Consumers, on the other hand, preferred NCS over CCS due to the following concerns. Is it possible for CCS to take over the role of NCS? What are the similarities and differences in chemical composition between CCS and NCS? In order to address these issues, it is necessary to comprehensively explore the chemical composition of both NCS and CCS, particularly an *in situ* chemical comparison between CCS and NCS. Based on a thorough chemical analysis, a rigorous quality control is of great importance and significance for *C. sinensis*.

For quality control of *C. sinensis*, a variety of technical platforms have been used, including LC-MS (Mi et al., 2016a; Mi et al., 2016b; Mi et al., 2018); GC-MS (Jianshuang Zhang et al., 2020), HPLC-UV (Li et al., 2004), CE-MS (Yang et al., 2009) and HPLC-ICP-MS (Zuo et al., 2018). However, the above analytical approaches can only measure either a class of components (or several constituents) or inorganic molecules which are usually extracted with specified solvents under specific conditions. There is still a need for an efficient analytical approach that can simultaneously measure and assess various types of components and multiple indicators, with no need for solvent extraction of target analytes to achieve *in situ* chemical analysis and imaging in samples.

Mass spectrometry imaging (MSI) is a label-free technique that provide the holistic methods for omics analyses (Bednarz et al., 2019). MSI is capable of offering abundant chemical clues and spatial distributions of analytes within sample at the same time. During MSI measurement, sample surface is rastered over by the ionization beam to record the x, y-coordinates of data set (Altelaar et al., 2005). Each pixel distributed on the sample surface comprised a complete mass spectrum (Altelaar, et al., 2005). MSI is increasingly applied to directly discover and visualize diverse metabolites of sample, revealing the underlying mechanisms or assisting for clinical diagnosis (Bednarz, et al., 2019; Fincher et al., 2019; Kriegsmann et al., 2019; Li et al., 2021; Pessotti et al., 2019).

In the past decades, matrix-assisted laser desorption/ionization (MALDI) is a common technique used in mass spectrometry imaging, which has been proved to be a mature tool to answer some research questions in the biomedicine (Kriegsmann, et al., 2019) and plant metabolomics (Zhan et al., 2021). In MALDI measurement, most of the incident particle energy is firstly adsorbed by the added matrix crystals, and then the energy will be transmitted to the sample molecules by matrix. Thus, the signals of large molecules such as protein and peptide can be provided by soft ionization of MALDI. However, MALDI has some inevitable limitations, such as matrix interferences due to the fact that the suitable matrix must be added to obtain signals during the process of sample pretreatment. It is also challenging for MALDI to detect the reliable metabolites of low-molecular weight (e.g., less than 800Da) due to the matrix interferences (Hu et al., 2021).

As a new emerging alternative, TOF-SIMS is a powerful MSI tool. Its primary ion beam impact on the sample surface directly without tiresome sample pretreatment, providing abundant data with combination of precise mass resolution, lateral and spatial resolution. With the development of cluster ion sources of liquid metal ion source (LMIG) and the gas cluster ion source (GCIB), TOF-SIMS is being used in the *in situ* chemical analysis and imaging of organic samples. It is a particularly good technique to detect and image many biochemical compounds particularly with molecular weight less than 1,000 Da with high sensitivity (Van Nuffel et al., 2020). Hence, TOF-SIMS has been increasingly used in analyses of biological samples including cells (Brison et al., 2013; Liu et al., 2017; Jia et al., 2020) and tissues (Hanrieder et al., 2013), as well as plants (Fu et al., 2018). However, it was rare for the application of TOF-SIMS in the research of TCMs, particularly an *in situ* chemical investigation on the raw materials of TCMs due to the high preparative demand for the samples whose surface should be flat, smooth and unbroken (Yoon and Lee, 2018). So far, only one research has utilized TOF-SIMS to investigate six constituents (proline, leucine, uracil, thymidine, mannitol and ergosterol) in pressed powder of *C. sinensis*, providing information on the changing trend of these six components in the grow cycle of *C. sinensis* (Xia et al., 2021). To further advance the application of TOF-SIMS in the research of TCMs, a double-layered media embedding strategy was firstly devised and established for an optimal cryosection sample preparation of CS in this study. By utilizing the established sample preparation approach, *in situ* chemical profiling and imaging were performed on the longitudinal slice of *C. sinensis* by TOF-SIMS. Various kinds of components including amino acids, nucleosides, monosaccharides and lipids, were comprehensively and simultaneously identified by both essential (required) and characteristic fragment ions with the aid of 67 reference compounds. Analyses of chemometrics were subsequently employed to investigate the chemical similarities and differences between CCS and NCS samples. Finally, the relative amount of glycerides obtained by TOF-SIMS in CCS and NCS samples was validated by using a quantitative ^1^H-NMR analytical approach.

## 2 Materials and Methods

### 2.1 Establishment of Personal Database of Chemical Components for *C. sinensis*

A personal database of *C. sinensis* was created based on the Reaxys database (ELSEVIER, https://www.reaxys.com/), the Dictionary of Natural Products ( http://dnp.chemnetbase.com/ ) and published TOF-SIMS investigations on amino acids, carbohydrates and lipids standards (Leefmann et al., 2013; Kawecki and Bernard, 2018a; Kawecki and Bernard, 2018b; Taylor et al., 2018; Bernard et al., 2019a; Bernard et al., 2019b) as well as other components (Debois et al., 2009; Frisz et al., 2012; Hou et al., 2021; Hua et al., 2018; Magnusson et al., 2008; Mi, et al., 2018; Mi, et al., 2016a; Sjövall et al., 2018; Urbini et al., 2017; Xia, et al., 2021; Yanyan Zhang et al., 2020). Moreover, the database was expanded to include a total of 67 TOF-SIMS-studied standards by the authors. In the personal database, over 700 compounds were specifically documented, including chemical names, corresponding molecular formulas, and chemical structures.

### 2.2 Materials and Reagents

Three batches of cultured *C. sinensis* (No.1–3, No. 4–6, No. 7–9) were bred in Yidu Hubei province, China. Two batches of natural *C. sinensis* (No. 10–12, No. 13–15) were collected from Qinghai province, China, and a batch of natural *C. sinensis* (No. 16–18) was obtained from Sichuan province, China. The freeze-dried cultured *C. sinensis* products and freshly frozen natural *C. sinensis* were all provided by the Horizon East company (HEC, Dongguan, China). Freshly frozen natural *C. sinensis* was dried under vacuum before sample preparation for qNMR determination. Tissue-Tek OCT compound was purchased from SAKURA (Nagano, Japan). Gelatin was bought from Yatai food additive company (Tianjin, China). Carboxymethyl cellulose was purchased from Haisheng cellulose derivative factory (Ningbo, China). Silicon wafers were purchased from Zhongjingkeyi Technology (Beijing, China). Glass slides and cryomolds were obtained from Thermo scientific (Massachusetts, United States). Sterilized blade was purchased from LEICA (Buffalo Grove, United States). Organic solvents of n-hexane (ACS grade), pentane (ACS grade), and iso-pentane (ACS grade) were bought from Tokyo chemical industry (Tokyo, Japan). Water (MS grade), dichloromethane (ACS grade), acetone (MS grade), methanol (MS and HPLC grade), and ethanol (HPCL grade) were bought from Anaqua chemicals supply Company Limited (Hong Kong, China). Deuterated chloroform was obtained from Shanghai Xianding Biotechnology (Shanghai, China). Benzoic acid *Trace*CERT^®^ was brought from Sigma-Aldrich (St. Louis, Mo, United States). The following reference standards were used for TOF-SIMS analysis. 20 amino acids (alanine, arginine, asparagine, aspartic acid, cysteine, glutamine, glutamic acid, glycine, histidine, isoleucine, leucine, lysine, methionine, phenylalanine, proline, serine, threonine, tryptophan, tyrosine, and valine), three sugars (d-fructose, d-glucose, and saccharose), 19 nucleotides (AMP, ADP, ATP, dAMP, dADP, GMP, GDP, GTP, dGMP, dGDP, CMP, CDP, CTP, dCMP, dCDP, UMP, UDP, UTP, and TMP), eight nucleosides (adenosine, cytidine, uridine, thymidine, 2′-deoxyadenosine, 2′-deoxycytidine, 2′-deoxyguanosine, and inosine), and six nucleobases (adenine, thymine, cytosine, guanine, uracil, and hypoxanthine) were purchased from Sigma-Aldrich (St. Louis, Mo, United States). Palmitic acid and arachidic acid were purchased from Shanghai Xianding Biotechnology (Shanghai, China). Stearic acid and palmitoleic acid were purchased from Alfa Aesar (Ward Hill, Ma, United States). Oleic acid standard was acquired from Tokyo chemical industry (Tokyo, Japan). 1,3-dioleoylglycerol was bought from Toronto Research Chemicals (Toronto, Canada). Phosphatidylcholine was obtained from Aladdin Biochemical Technology (Shanghai, China). Ergosterol, ergosterol peroxide, cholesterol, and mannitol were purchased from National Institutes for Food and Drug Control (Beijing, China).

### 2.3 Preparation of Reference Standards

For TOF-SIMS analysis, each standard was prepared in solvents at a final concentration of 1 mg/ml. Palmitic acid, oleic acid, stearic acid and phosphatidylcholine were dissolved in ethanol. Fructose, glucose, sucrose, arginine, histidine, phenylalanine, tryptophan, alanine, valine, serine, glycine, proline, threonine, leucine, iso-leucine, cysteine, methionine, asparagine, glutamine and adenosine, cytidine, thymidine, 2′-deoxyadenosine, 2′-deoxycytidine, 2′-deoxyguanosine, AMP, ADP, ATP, GMP, GDP, GTP, UMP, UDP, UTP, CMP, CDP, CTP, TMP, inosine, 2′-dAMP, 2′-dADP, 2′-dCMP, 2′-dCDP, 2′-dGMP and 2′-dGDP were dissolved in 50% methanol. Lysine, tyrosine, aspartic acid and glutamic acid were prepared in 50% aqueous methanol containing 0.5% HCl. Uridine, adenine, thymine, cytosine, guanine, uracil, hypoxanthine, cholesterol, ergosterol, ergosterol peroxide and mannitol were prepared in methanol. 1,3-dioleoylglycerol was dissolved in n-hexane. For TOF-SIMS analysis, 20 μL of each standard solution was deposited onto silicon wafer. Those silicon wafers were allowed to dry in a biologically safe cabinet.

### 2.4. Preparation of Embedding Media and Pretreatment of Silicon Wafers

The solutions of 10% gelatin (W/V) and 4% CMC (W/V) were prepared according to the reported approaches (Li et al., 2018). The new silicon wafers were washed with water after being soaked in MS grade water overnight. Then the silicon wafers were sonicated for 5 min each in methanol, acetone and dichloromethane consecutively, and the procedure was repeated twice. Finally, the cleaned silicon wafers were allowed to dry at ambient temperature in a biologically safe environment (Taylor, et al., 2018).

### 2.5 A Double-Layered Media Embedding Approach for Cryosection Sample Preparation

A two-layered media embedding approach was devised and optimized to prepare the sliced samples of *C. sinensis* for TOF-SIMS analysis as follows. The freeze-dried samples were initially wrapped in dust-free tissue and moistened with a few MS grade water before being placed in a vacuum tank for 3 h to restore its original forms. The shape-restored caterpillar samples were immersed in the first medium (10% gelatin) and placed in an embedding mold for 2 min of solidification before entirely being embedded in the second medium (OCT). The double-coated samples were immediately frozen in the liquid N_2_-precooled iso-pentane (Kawamoto, 2003) for 3 min. The above preparation of frozen blocks of *C. sinensis* samples was named as a double-layered media embedding method. The frozen blocks were kept in a -80°C freezer for storage. For the preparation of sliced sample, the frozen blocks were placed in the CM1860 cryostat (Leica Biosystems, Wetzlar, Germany) at −20°C about at least half an hour for temperature equilibrium. The frozen block of sample was then sliced into 10 μm sections and thaw-mounted on silicon wafers for subsequent TOF-SIMS analysis. In this study, a total of 18 whole caterpillars were used for *in situ* chemical profiling and imaging of cultured and wild *C. sinensis* by TOF-SIMS (both are three batches, *n* = 3 in each batch).

### 2.6 TOF-SIMS Measurement

*In situ* chemical profiling of cultured and natural *C. sinensis* was performed on a commercial TOF-SIMS V instrument (ION-TOF GmbH, Munster, Germany) equipped with a bismuth (Bi) liquid metal ion gun (LMIG) as a primary ion source. The secondary ion spectra and images were recorded in spectrometry mode in both positive and negative polarities. The primary ion beam, delivering 30 keV Bi_3_^+^, was impacted on the analyte at an incidence angle of 45° with a pulse current of ∼0.40 pA and a pulse width of ∼20 ns. The cycle time was 150 µs, covering the secondary ion mass range of 0–2016 Da. The short pulse width offered the high mass resolution of the M/ΔM range mainly from 4,000 to 7,000 in bunched mode in this research. The primary ion beam was rastered on a field of 200 × 200 μm^2^ with 128 pixels × 128 pixels^2^ for analyses of standard compounds. The longitudinal slices with large areas were rastered stepwise with each pitch of 500 × 500 μm^2^ and 100 × 100 random raster pixel^2^ by using the stage raster mode. The pitches were joined together for a complete view of 42 × 16 mm^2^ of longitudinal section. Fifty scans were collected for standards and three scans were recorded on vertical slices in each measurement. The primary ion dose density was maintained below 10^12^ ions/cm^2^ in static SIMS condition. A pulsed low-energy electron flood gun of ∼20 eV was used to compensate charging effects during the acquisition. For standard spectra, interval mass calibrations were performed using ions of C^+^, C_3_H_7_^+^, and C_4_H_9_^+^ in positive polarity, or CH^−^, C_2_H^−^, C_3_H^−^, C_4_H^−^, and C_5_H^−^ were used in the negative polarity. Actually, some characteristic ions would be added for more accurate mass calibration. For instance, NH_3_^+^, CH_4_N^+^, C_2_H_4_N^+^ and C_10_H_15_N_5_O_4_^+^ were also added to internally calibrate the positive spectra of adenosine in addition to the aforementioned ions. For CS sample slices, positive spectra were internally calibrated with the common ions of C_4_^+^, C_6_H_13_^+^, C_8_H_13_^+^, C_9_H_13_^+^, C_5_H_13_NPO_3_^+^, C_5_H_15_NPO_4_^+^, C_18_H_33_O^+^ C_37_H_69_O_4_^+^, and C_37_H_71_O_4_^+^. The ions of CH^−^, C_2_H^−^, C_3_H^−^, C_4_H^−^, C_5_H^−^, C_16_H_29_O_2_^−^, C_16_H_31_O_2_^−^, and C_18_H_32_O_2_^−^were applied in the negative spectra.

### 2.7 TOF-SIMS Data Analysis

#### 2.7.1 TOF-SIMS Data Processing

The processing of TOF-SIMS mass data and mass spectrometry images was conducted by using SurfaceLab 6.7 software. The function of regions of interest (ROI) was firstly applied to remove the matrixes that embraced the sample. The mass data and mass spectrometry images merely characterized for caterpillars could be therefore extracted by reconstructing the individual ROIs. Automatic peak selection was carried out by the software under the conditions of *m*/*z* ranging from 0 to 1,000, a signal-to-noise ratio (SNR) of at least 3, and the minimum counts of 3,000. Each automatically generated peak list was checked manually to exclude the overlapped peaks and add some characteristic peaks with low intensity. All peak lists of CCS or NCS samples (n = 9) were united to maximize the inclusion of all detected ion peaks for subsequent chemical identification in both positive and negative modes. Furthermore, the peak lists of both CCS and NCS samples (n = 18) were also combined to generate a common mass interval list for multivariate statistical analysis and similarity evaluation. Each dataset of peak areas was normalized by the primary ion dose (PID) to decrease the systematic differences, and individual differences in sample size.

#### 2.7.2 Similarity Evaluation

Fingerprint similarity is an important criterion in quality control of TCMs. The similarities of TOF-SIMS spectra between CCS and NCS samples were herein calculated by Cosine coefficient (Peng et al., 2011) and Tanimoto coefficient (Bajusz et al., 2015), respectively. The Cosine coefficient was the basic algorithm of the Chinese Pharmacopoeia Committee’s similarity evaluation system for chromatographic fingerprints in TCMs. The TOF-SIMS datasets (n = 18) were imported into Microsoft Excel (Microsoft 2016; Roselle, IL, United States), and computed by the following formulae:

Cosine coefficient:

$$r_{A,B}=\frac{\sum_{\mathrm{i=}1}^{n}x_{\mathrm{iA}}x_{\mathrm{iB}}}{\sqrt{\sum_{i=1}^{n}{\left(x_{iA}\right)}^{2}\times \sum_{i=1}^{n}{\left(x_{iB}\right)}^{2}}}$$

Tanimoto coefficient:

$$r_{A,B}=\frac{\sum_{\mathrm{i=}1}^{n}x_{\mathrm{iA}}x_{\mathrm{iB}}}{\sum_{i=1}^{n}{\left(x_{iA}\right)}^{2}+\sum_{i=1}^{n}{\left(x_{iB}\right)}^{2}-\sum_{\mathrm{i=}1}^{n}x_{\mathrm{iA}}x_{\mathrm{iB}}}$$

The $r_{A,B}$ represents the correlation coefficient between fingerprint $x_{iA}$ and reference fingerprint $x_{iB}$ . Reference fingerprint was generated by the average of normalized peak areas of all samples. The $x_{iA}$ and $x_{iB}$ are the normalized areas of the *i*th peak of each spectrum. The value of the correlation coefficient is in the range $0<r_{A,B}\leq 1$ . The larger $r_{A,B}$ value means higher similarity. If the correlation coefficient is above 0.9, between 0.8 and 0.9, or below 0.8, it is considered as the best, the better or the worst similarity, respectively. When $r_{A,B}$ equals 1, it is regarded as identical ones (Peng, et al., 2011).

#### 2.7.3 Multivariate Statistical Analysis

The datasets of normalized peak areas of CCS and NCS (n = 18) were imported into SIMCA 15 software (Umetrics, umea, Sweden) for multivariate statistical analysis. The 3-D matrix was consisted of sample names (observations), chemical m/z (variables) and the normalized peak areas. Principal component analysis (PCA) was firstly used to depict the intrinsic similarities and differences between CCS and NCS. Then, orthogonal partial least-squares discriminant analysis (OPLS-DA) was carried out to identify the differentially expressed constituents which contributed to the separation of CCS and NCS. The differentially expressed components also needed to meet the statistical significance (*p* < 0.05) of the Student’s t-test analysis. Hierarchical cluster analysis (HCA) was additionally employed to depict the general clustering between CCS and NCS.

#### 2.7.4 Relative Quantification of Chemical Components by TOF-SIMS

The identified ions were concretely divided into amino acids, nucleosides, fatty acids, glycerides, glycerophospholipids, sphingolipids, sterols, monosaccharides, mannitol, vitamin E and so on. The contents of those components were calculated with the primary ion dose normalized peak areas. The total content was the sum of normalized peak areas of all detected molecular ions. Amino acids, fatty acids, PIs and PAs were calculated with the negative spectra data, while other chemical components were quantified by the positive spectra data. Data were expressed as average ± standard deviation (n = 9). Student’s t-test was carried out to determine the statistical significance (*p* < 0.05) of the contents between CCS and NCS samples by using SPSS 26.0 (SPSS Inc., United States).

### 2.8 The qNMR Determination of Glycerides

#### 2.8.1 Extraction of Glycerides in Cultured and Natural *C. sinensis*

Total 18 pieces of CS samples mentioned above were ground into powder, and each sample’s powder (∼60 mg) was accurately weighed. Sample was accurately added 3 ml of methanol, vortexed for 1 min, and then sonicated (220W, 50–60 kHZ) for 20 min. The mixture was centrifuged at 4,000 rpm for 10 min. The residue sample was extracted one more time in the same way. The supernatants were combined and dried by N_2_ gas flow, and then placed in a vacuum desiccator for 24 h prior to qNMR analysis.

#### 2.8.2 Preparation of Internal Standard Solution

A suitable glass vial equipped with a screwing cap was accurately weighed. The precise weight of internal standard benzoic acid (∼11 mg) and CDCl_3_ (∼25 ml) was successively added into the vial. Immediately, the vial was sealed and gently shaken until the benzoic acid was completely dissolved. Finally, total weight of mixture and vial was recorded and the exact concentration of IS solution was calculated.

#### 2.8.3 ^1^H-NMR Measurement

The dried extract of sample was dissolved in 900 μL IS solution. The reconstituted solution was filtered by a piece of defatted cotton and at least 500 μL of the subsequent filtrate was transferred to a 5 mm NMR tube. The ^1^H-NMR spectra were recorded on a 600 MHz NMR spectrometer (Bruker Ascend, Karlsruhe, Germany) with a cryoprobe by employing the following parameters: temperature at 298K, relaxation delay = 40 s, number of scans = 64, number of dummy scans = 16, acquisition time = 8.41s (128K points), and spectra width 13.0 ppm centered at 6.2 ppm (Saraf et al., 2021).

#### 2.8.4.^1^H-NMR Data Analysis

All the spectra were phased and baseline-corrected by using TopSpin 3.1 software (Bruker Analytische GmbH, Rheinstetten, Germany). The assignments of signals were matched by the chemical shifts (ppm) and splitting patterns. The signal of solvent (CDCl_3_) was observed at δ 7.26 ppm. The signal of internal standard (IS) of benzoic acid at δ 7.60 ppm was integrated to 100 for the quantification of glyceride in CS samples. The specific signals of *sn*-2 proton in various glycerides from the analyte were integrated. The moles of glycerides in CCS and NCS samples were computed by the following equations:

$$n\left(IS\right)=\frac{m\left(IS\right)}{m\left(IS+CDCl_{3}\right)}\times \frac{m\left(IS\;solution\right)}{M\left(IS\right)}$$ (1)

$$n\left(x\right)=\frac{A\left(x\right)\times N\left(IS\right)\times n\left(Is\right)}{A\left(IS\right)\times N\left(x\right)}$$ (2)

Where n(IS) is the calculated mole of benzoic acid added into each sample,  m(IS) is the weight of benzoic acid, $m\left(IS+CDCl_{3}\right)$ is the total weight of benzoic acid and CDCl_3_ initially used for the preparation of homologous internal standard solution, m(IS solution) is the weight of IS solution added into each sample, M(IS) is the molar mass of benzoic acid, A(x) is the integral area of *sn*-2 protonated signal of glyceride, A(IS) is the integral area of protonated IS signal at *δ* 7.60 [ A(IS) was calibrated to 100], N(x) is the number of protons for the integrated signal in the molecule of glyceride, N(IS) is the number of protons for the integrated signal (*δ* 7.60) in the molecule of benzoic acid. Both N(x) and N(IS) equal one in this study.

The amounts of glycerides in CCS and NCS samples were finally expressed as the number of moles per gram (mole/g, average ± standard deviation, n = 9). The data were further statistically analyzed by Student’s t-test. The level of statistical significance was set at 0.05. When more than one signal was assigned to each type of identified glyceride, such as DAGs consisting of 1-DAG and 2-DAG, the integrals of segmented ^1^H-NMR signals were summed for statistical analysis.

## 3 Results and Discussion

### 3.1 Establishment of an Optimal Double-Layered Media Embedding Method for *in situ* Chemical Analysis

**FIGURE 1:**
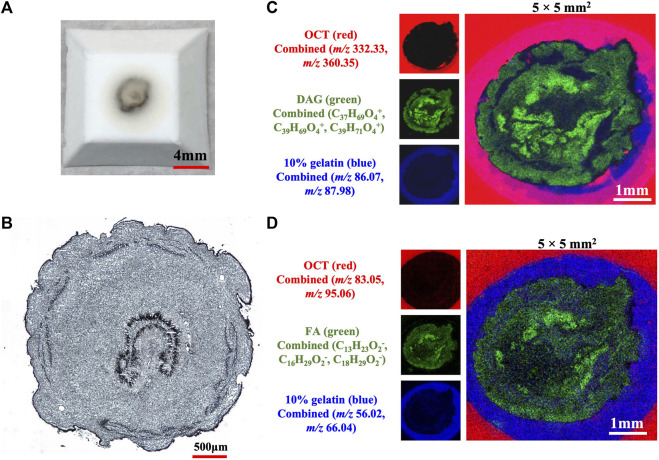
The sample prepared with a double-layered media embedding method **(A)** The solid block **(B)** The complete sample slice under a microscope **(C)** MSIs demonstrating the overlay of ions of OCT, 10% gelatin and DAGs in positive mode **(D)** MSIs exhibiting the overlay of ions of OCT, 10% gelatin and FAs in negative mode. The sample block embedded with 10% gelatin and OCT was frozen in the pre-cooled iso-pentane.

TOF-SIMS has the advantage of direct analyzing samples without applying extra organic molecules to the sample to assist ionization and allow for label-free detection. However, sample preparation is essential for TOF-SIMS analysis to keep the natural state of the components and provide precise spatial distribution information in samples. Therefore, the cryosection sample preparation suitable for TOF-SIMS analysis was firstly investigated including the softening of the dried caterpillars, the proper embedding medium, the suitable coolant, the cooling time, the completeness of the frozen solid block, the integrity of the cryosection, and so on. More critically, an ideal caterpillar cryosection for TOF-SIMS analysis should meet the following two key requirements. One is no matrix impact on the analytes caused by the embedding medium, and the other is no chemical migrations in the caterpillar sample. A double-layered media embedding strategy was finally refined for the establishment of an ideal caterpillar cryosection based on a series of sample preparation optimizations according to the aforesaid requirements. As shown in Figure 1, a nice frozen sample block was obtained, and the cryosection was also smooth and complete. In both positive and negative modes, there was no evident matrix effect on the sample and no chemical migrations. Therefore, this double-layered sample coating approach was employed for the TOF-SIMS examination of a longitudinal segment of *C. sinensis*. More detailed description of optimization was depicted in the supporting information (frozen blocks in Supplementary Figure S1, cryosections in Supplementary Figures S2-S3, matrix impacts in Supplementary Figures S4-S9, chemical migrations in Supplementary Figures S10-S11).

### 3.2 TOF-SIMS Analysis of Cultured and Natural *C. sinensis*

#### 3.2.1 TOF-SIMS Study on Standard Chemicals

**FIGURE 2:**
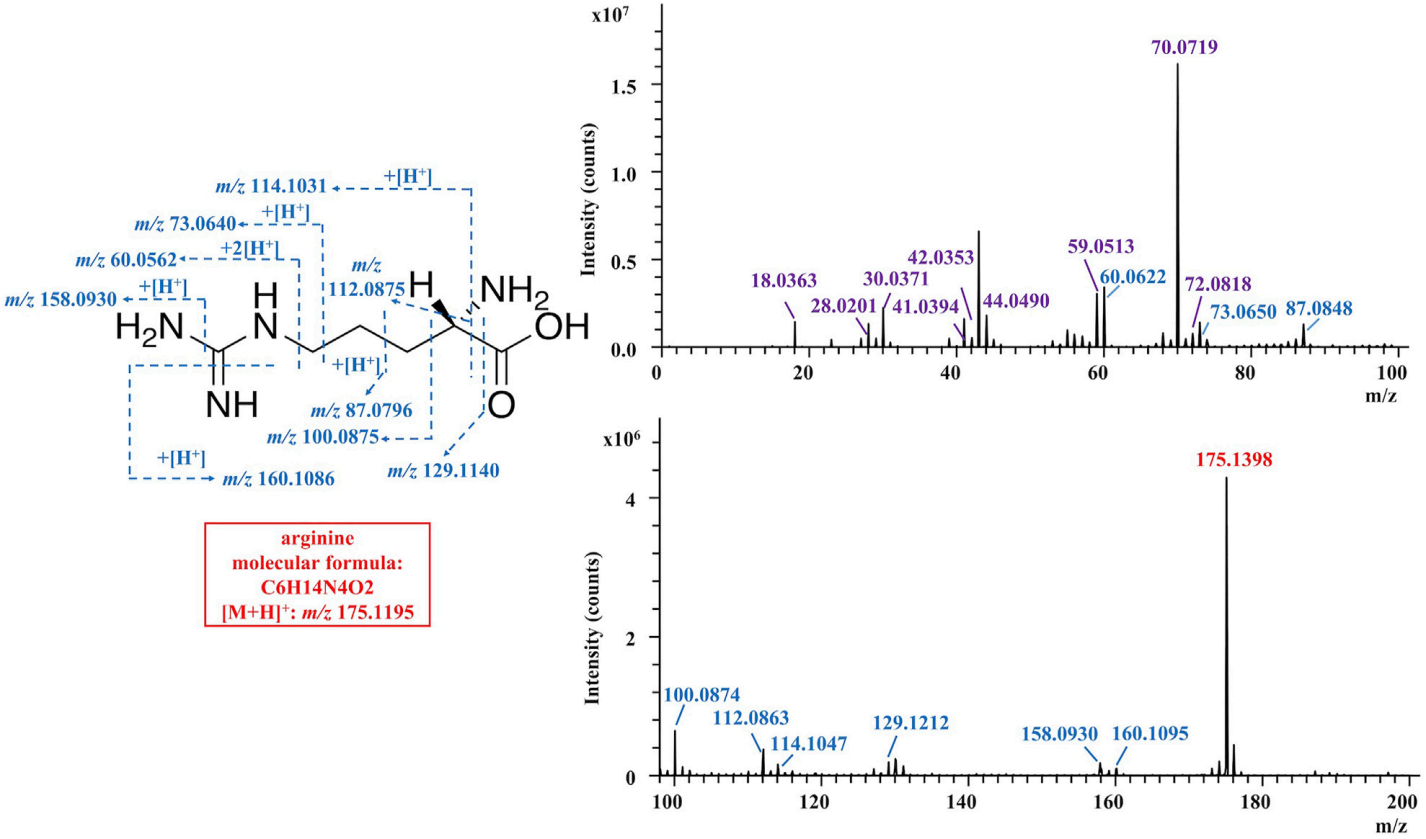
TOF-SIMS spectra of arginine standard in the positive mode.

To gather information on ionization behaviors and cleavage properties in TOF-SIMS, a total of 67 standard compounds were initially investigated by TOF-SIMS, including 20 amino acids, six nucleobases, eight nucleosides, 19 nucleotides, one disaccharide, two monosaccharides, one diacylglycerol (DAG), one phosphatidylcholine (PC), five fatty acids, three sterols and mannitol. An array of TOF-SIMS spectra and tabulated identified ions of representative standards were provided in the supplemental information (Supplementary Figures S12-S32, Supplementary Tables S1-S21). Both the essential ions and characteristic fragment ions were considered as decisive evidences for the identification of a component. The essential ions were defined as the required ions that are either the molecular ions or quasi-molecular ions including the protonated or deprotonated ion, sodium adduct ion, and potassium adduct ion, etc. The characteristic fragment ions were classified as the ions resulted from the removals of any functional group (including amino group, carboxyl group, hydroxyl group, alkyl group, and side chain, etc.) from the intact molecule, which supplied important supporting information for the assignment of parental compounds. For instance, the specific assignment of arginine (Figure 2, Table 1) was firstly attributed to the protonated molecular ion at m/z 175.1398 ([M + H]^+^). At the same time, the supporting evidence for the identification was provided by the characteristic fragment ions at *m/z* 158.0880 ([M-(NH_3_)+H]^+^), *m/z* 129.1212 ([M-(COOH)]^+^), *m/z* 114.1047 ([M-(NH_2_)-(COOH)+H]^+^) and *m/z* 112.0863 ([M-(NH_3_)-(COOH)]^+^). For each type of standard compound, the ionization patterns and cleavage features of both essential ions and characteristic fragment ions were summarized as follows in order to aid further comprehensive chemical identifications in *C. sinensis* samples. Firstly, the essential ions dominated in the TOF-SIMS spectra of amino acids, with both deaminated and decarboxylated ions serving as the middle abundance of characteristic fragment ions (Supplementary Figures S12-S14, and Supplementary Figure S27,S28). Secondly, due to the easy cleavage of the glycosidic bond under the high energy of the primary ion beam, the nucleobases’ ions were predominant in the nucleosides’ TOF-SIMS spectra compared to the corresponding essential ions (Supplementary Figure S15-S19, and Supplementary Figure S31). Similar phenomena were observed with nucleotides, in which the corresponding nucleobases showed dominating ion peaks whereas the parental nucleotide ions were barely visible. Thirdly, essential ions predominated in three monosaccharides (glucose, fructose and mannitol), followed by a succession of dehydrated ions (Supplementary Figure S20-S22, and Supplementary Figure S32). Fourthly, both essential and dehydrated ions were predominant in the TOF-SIMS spectrum of ergosterol (Supplementary Figure S24), whereas the dehydrated ion became the only predominant ion in the TOF-SIMS spectrum of cholesterol with a minor essential ion (Supplementary Figure S23). Fifthly, in the TOF-SIMS spectrum of 1,3-dioleoglyceride, a dehydrated monooleoylglycerol ion of [M-(H_2_O)+H]^+^ was the major ion, followed by the ion of dehydrated 1,3-dioleoglyceride of [M-(H_2_O)+H]^+^ and fatty acyl ions, as well as a trace abundance of the essential ion (Supplementary Figure S25). Finally, fatty acids were dominated by molecular ions, followed by a series of ions that removed numerous–CH2 groups (Supplementary Figure S29,S30).

**TABLE 1:** The peak assignments of arginine standard in TOF-SIMS spectra in the positive mode.

No	Observed mass (*m/z*)	Chemical formula	Deviation (ppm)	Species
required peaks				
1	175.1398	C_6_H_15_N_4_O_2_^+^	119.2	[M + H]^+^
characteristic peaks				
2	160.1095	C_6_H_14_N_3_O_2_^+^	9.0	[M-(NH)+H]^+^
3	158.0880	C_6_H_12_N_3_O_2_^+^	−28.0	[M-(NH_3_)+H]^+^
4	129.1212	C_5_H_13_N_4_^+^	59.6	[M-(COOH)]^+^
5	114.1047	C_5_H_12_N_3_^+^	18.7	[M-(NH_2_)-(COOH)+H]^+^
6	112.0863	C_5_H_10_N_3_^+^	−5.5	[M-(NH_3_)-(COOH)]^+^
7	100.0874	C_4_H_10_N_3_^+^	5.1	[M-(CH_3_N)-(COOH)]^+^
8	87.0848	C_3_H_9_N_3_^+^	65.6	[M-(C_3_H_6_NO_2_)+H]^+^
9	73.0650	C_2_H_7_N_3_^+^	20.6	[M-(C_4_H_8_NO_2_)+H]^+^
10	60.0622	CH_6_N_3_^+^	108.8	[M-(C_5_H_9_NO_2_)+H]^+^
other peaks				
11	72.0818	C_4_H_10_N^+^	14.2	—
12	70.0719	C_4_H_8_N^+^	96.1	—
13	59.0513	C_3_H_7_O^+^	37.0	—
14	44.0490	C_2_H_6_N^+^	-10.8	—
15	42.0353	C_2_H_4_N^+^	35.4	—
16	41.0394	C_3_H_5_^+^	19.6	—
17	30.0371	CH_4_N^+^	109.1	—
18	28.0201	CH_2_N^+^	68.7	—
19	18.0363	NH_4_^+^	136.0	—

#### 3.2.2 *In situ* Chemical Identification in Cultured and Natural *C. sinensis* by TOF-SIMS

**FIGURE 3:**
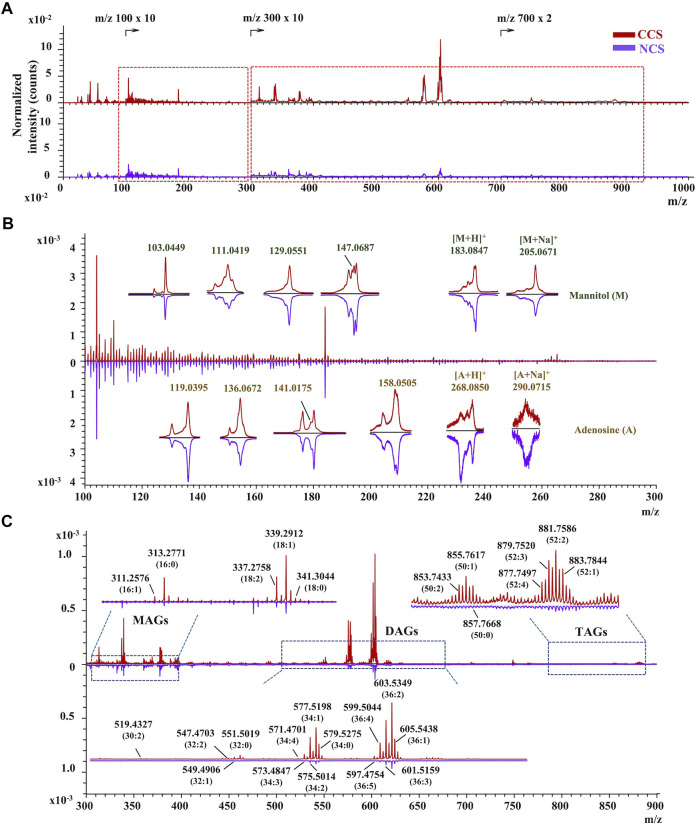
The TOF-SIMS spectra of CCS and NCS samples in positive mode. **(A)** The typical spectra of CCS (No. 6 in red) and NCS (No. 13 in purple) samples. **(B)** Identification of mannitol and adenosine with both essential and characteristic fragment ions in the magnified region with m/z value ranging from 100 to 300. **(C)** Characterization of representative MAGs, DAGs and TAGs in the enlarged region with m/z value from 300 to 900. The TOF-SIMS spectra were normalized by primary ion dose (PID).

**FIGURE 4:**
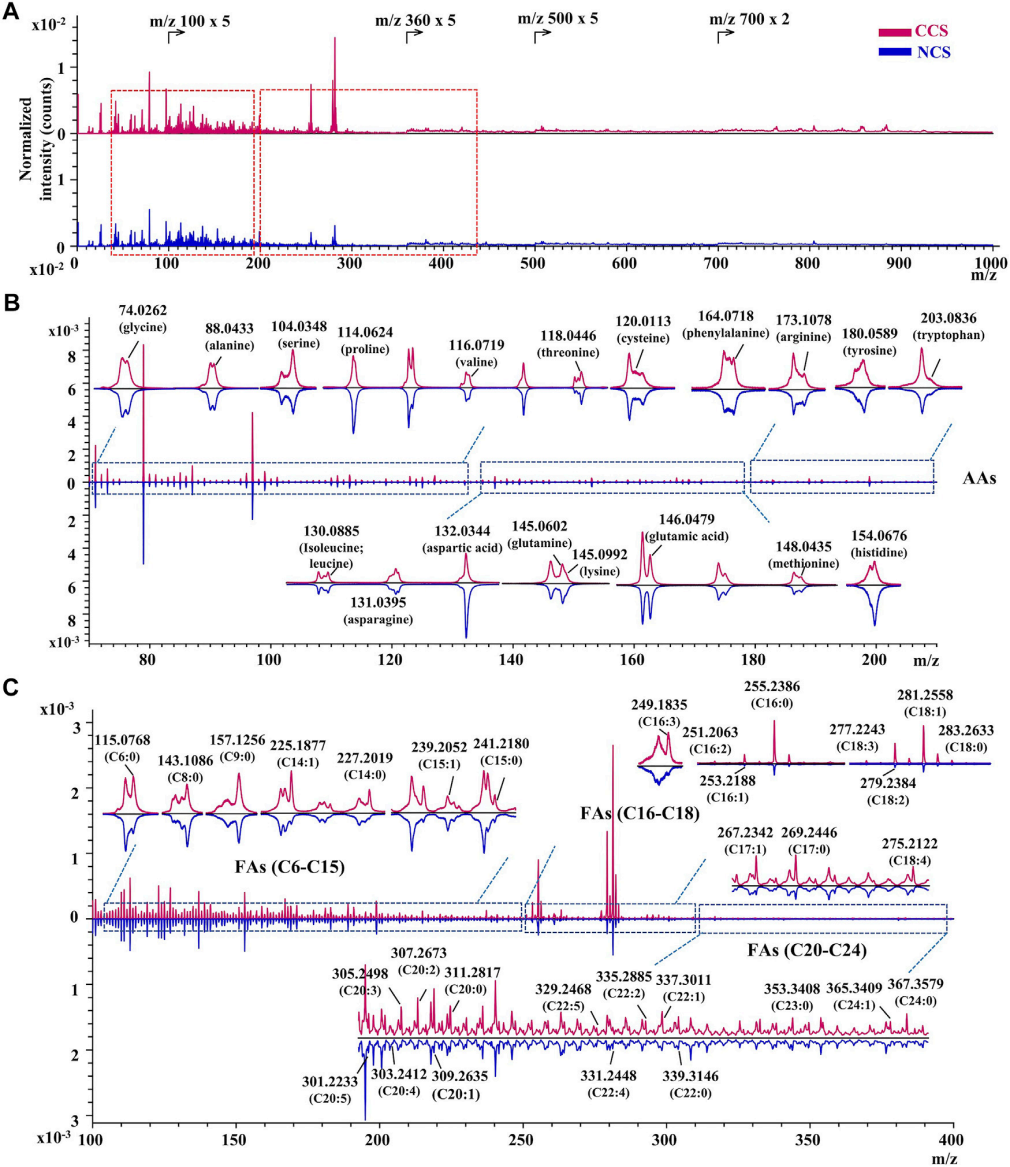
The TOF-SIMS spectra of CCS and NCS samples in negative mode. **(A)** The representative spectra of CCS (No. 4 in red) and NCS (No. 14 in blue) samples. **(B)** Identification of amino acids (AAs) in the magnified region with m/z value from 70 to 210. **(C)** Assignment of fatty acids in the magnified region with m/z value from 100 to 400. The TOF-SIMS spectra were normalized by PID.

*In situ* chemical profiling of caterpillars of CCS and NCS were performed based on the aforesaid ionization and fragmentation laws of various types of components in both positive and negative modes (Supplementary Figure S33,S34). One typical spectrum from each of the CCS and NCS was reversely superimposed and magnified for a detailed and complete comparison (Supplementary Figure S35,S36). In the united peak list of CCS samples (n = 9), more than 950 and 650 ion peaks were obtained in positive and negative mode, respectively. In NCS samples (n = 9), more than 900 and 450 ion peaks were acquired in positive and negative mode, respectively. These ions were then empirically assigned and tentatively identified based on the mass accuracy and valence rule supported by the established database. As the results (Supplementary Tables S25 in excel format), more than 218 chemical constituents were tentatively characterized from both CCS (positive ions identification in Supplementary Tables S25A; negative ions identification in Supplementary Tables S25C) and NCS (positive ions identification in Supplementary Tables S25B; negative ions identification in Supplementary Tables S25D) samples, including 20 amino acids, 6 nucleobases, 7 nucleosides, 181 lipids, vitamin E, 2 sugar alcohol (mannitol and myo-inositol), and 1 phenol (maltol). The lipids included 32 fatty acids, 28 glycerides (5 MAGs, 14 DAGs, and 9 TAGs), 10 glycerophospholipids (4 phosphatidylcholines, 4 phosphatidylinositols and 2 phosphatidic acids), 97 sphingolipids (15 sphingoid bases, 50 ceramides, 18 SMs, 14 HexCers) and 14 sterols. In addition, several monosaccharides were also detected in this study. The above identified chemical components should be recognizable by at least their essential ions. Moreover, most of constituents’ characterization were validated by their characteristic fragment ions. The specific *m*/*z* regions of representative ion peaks were magnified including adenosine, mannitol, MAGs, DAGs, TAGs, amino acids, and fatty acids (Figures 3, 4). The other enlarged ion peaks of typical compounds containing sphingoid bases, sterols, ceramide analogues, sphingomyelins, and glycosphingolipids were displayed in Supplementary Figure S37,S38. In summary, the chemical compositions of CCS and NCS were essentially identical, according to the full *in situ* chemical analysis, with the exception that the CCS samples contained more lipids.

### 3.3 Chemical Comparison Between Cultured and Natural *C. sinensis*

#### 3.3.1 Similarity Calculation of Cultured and Natural *C. sinensis*

The datasets of detected ions in the *m/z* range from 100 to 1,000 were utilized for the similarity evaluation between CCS and NCS samples in both positive and negative modes. The ions below *m/z* 100 were excluded since there were almost non-characteristic fragments or inorganic ions. As a result, there was a total of 845 positive ions and 573 negative ions screened out for similarity calculation by Cosine and Tanimoto coefficients (Supplementary Tables S22). All calculated Cosine coefficients ranged from 0.9918 to 0.8767 except for a value of 0.6532 in No.13 sample of NCS. Tanimoto coefficients varied from 0.9836 to 0.8127 in the positive mode and were more than 0.8 for most CS samples in the negative mode. Both correlation coefficients suggested CCS and NCS were very comparable. This finding suggested that CCS may be expected to alleviate dilemmas of NCS, including being an endangered species, being subjected to soaring prices, and being environmentally damaging due to over-collection.

#### 3.3.2 Multivariate Statistical Analysis of Cultured and Natural *C. sinensis*

**FIGURE 5:**
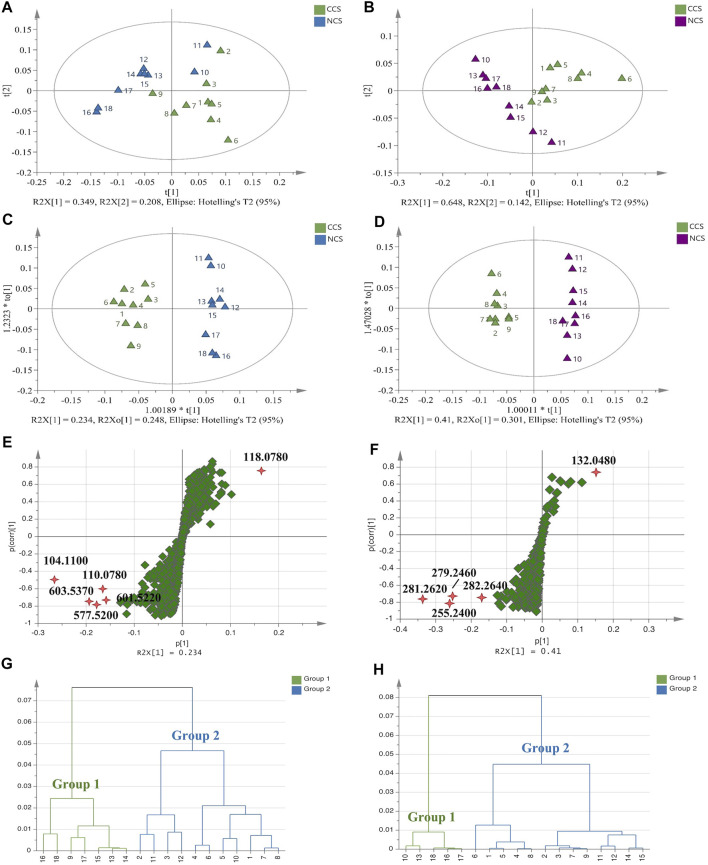
Multivariate statistical analysis of TOF-SIMS data of all CCS and NCS samples. **(A,B)**: PCA score plots. **(C,D)**: OPLS-DA score plots. **(E,F)**: S-plot diagrams. **(G,H)**: HCA dendrograms. **(A,C,E,G)**: Data in positive mode. **(B,D,F,H)**: Data in negative mode.

Principal component analysis (PCA) and orthogonal partial least-squares discriminant analysis (OPLS-DA) were further applied to compare CCS and NCS samples with the above datasets by using SIMCA software. In PCA score plots (Figure 5A,B), CCS and NCS had a tendency to separate but could not be distinguished in both positive and negative polarities, indicating a high degree of similarity in their components. The supervised model of OPLA-DA was then employed to differentiate CCS and NCS with grouping information provided in advance. In positive mode, the supervised model parameters of R^2^Y (cum) and Q^2^ (cum) of OPLA-DA were 96 and 75.3%, respectively. In negative polarity, the values of R^2^Y (cum) and Q^2^ (cum) were 97.9 and 86.5%, respectively. These R^2^Y (cum) and Q^2^ (cum) values indicated that the models were fit and predicted correctly. As the result (Figure 5C,D), CCS and NCS were clearly separated into two clusters in the OPLS-DA score plots. It was worth noting that the CCS points were relatively concentrated, whereas the NCS points tended to be discrete. S-plots were used to screen out the variables responsible for the separation. Variables far from the origin often contribute more to separation in the S-plot. The points of |*p*| > 0.15 and |*p* (corr)| > 0.4 were regarded as contributing significantly to clustering and separation in this study (Figure 5E,F). In positive mode (Supplementary Tables S23), the differentially expressed constituents between CCS and NCS were identified as ions with *m*/*z* 104.1100 (C_5_H_14_NO^+^, PC fragment), *m*/*z* 603.5370 [C_39_H_71_O_4_^+^, DAG (36:2)], *m*/*z* 577.5200 [C_37_H_69_O_4_^+^, DAG (34:1)], *m*/*z* 110.0780 (C_5_H_8_N_3_^+^, arginine and histidine fragments), and *m*/*z* 601.5220 [C_39_H_69_O_4_^+^, DAG (36:3)]. In negative mode (Supplementary Tables S24), the differential constituents were characterized as ions with *m*/*z* 281.2620 [C_18_H_33_O_2_^−^, oleic acid (18:1)], *m*/*z* 255.2400 [C_16_H_31_O_2_^−^, palmitic acid (C16:0)], *m*/*z* 279.2460 [C_18_H_31_O_2_^−^, linoleic acid (C18:2)], *m*/*z* 282.2640 [C_18_H_34_O_2_^−^, oleic acid (18:1)] and *m*/*z* 132.0480 [C_4_H_6_NO_4_^−^, aspartic acid]. Student’s t-test analysis was performed to further confirm these differentially expressed components. As shown in Supplementary Figure S39, the OPLS-DA models were demonstrated not overfitting by 200 iterations of replacement experiments in this study. The HCA analysis was conducted to further compare CCS with NCS with the fact that the shorter distance between samples meant less discrepancy. As the result, 18 samples were divided into two main clusters. In positive mode (Figure 5G), cluster I contained samples of No. 9, 13, 14, 15, 16, 17 and 18. The other samples No. 1, 2, 3, 4, 5, 6, 7, 8, 10, 11 and 12 were gathered in cluster II. In negative mode (Figure 5H), cluster I included samples of No. 10, 13, 16, 17 and 18, while samples of No. 1, 2, 3, 4, 5, 6, 7, 8, 9, 11, 12, 14, and 15 were gathered in cluster II. The results of HCA were in line with those of PCA and OPLS-DA. Taken together, the following findings were summarized. Firstly, the distances were greater between NCS samples than those between CCS samples, indicating higher chemical variations in NCS samples. Secondly, the chemical components of CCS were more consistent than those of NCS samples, as evidenced by the fact that CCS sample distances were closer than those of NCS sample. Finally, several NCS samples were clustered with CCS, implying that CCS had a high degree of similarity with some NCS.

#### 3.3.3 Quantitative Comparison of Cultured and Natural *C. sinensis* by TOF-SIMS

For quantitative comparisons of primary groups of components, the sum of peak areas of their essential ions was calculated between CCS and NCS samples. A statistical analysis was conducted by Student’s t-test. As shown in Table 2, significant differences were not discovered in the amounts of total amino acids, total nucleosides, total monosaccharides, total sterols and total sphingolipids between CCS and NCS samples. However, the contents of total fatty acids (*p* < 0.01), total glycerides (*p* < 0.001), total glycerophospholipids (*p* < 0.05), total ceramides (*p* < 0.01) and total Hexcers (*p* < 0.05) were remarkably higher in CCS samples than the counterparts in NCS samples. In particular, it was discovered that MAGs (*p* < 0.001), DAGs (*p* < 0.001) and TAGs (*p* < 0.001) all contributed to the crucial disparity in total glycerides between CCS and NCS samples. The major variation in glycerophospholipids between CCS and NCS samples was due to PIs (*p* < 0.01), as there were no significant changes in the other two subclasses of PCs and PAs. In terms of sphingolipids, total ceramides (*p* < 0.01) and total HexCers (*p* < 0.05) in CCS were considerably higher than those in NCS, but total sphingoid bases and total SMs did not differ significantly between NCS and CCS samples. As a result, there was no statistical difference in total sphingolipids between the NCS and CCS samples. As two representative individual components, mannitol and vitamin E also did not show any difference between CCS and NCS samples. Taken together, CCS has comparable proportions of major kinds of chemical components to NCS, including amino acids, nucleosides, monosaccharides, sphingolipids, sterols, and two common bioactive components (mannitol and vitamin E), which was in line with the previous studies (Guo et al., 2017; Ke et al., 2018; Bin Li et al., 2018; Zan et al., 2017; Zhao, et al., 2014). However, CCS possessed a relatively high lipid level in fatty acids, glycerides, and glycerophospholipids. Some research has been reported that neutral lipids, including TAGs, DAGs, and MAGs, are essential in energy storage. Moreover, some of them such as DAGs also act as second messengers in lipid signaling and help maintain sterol homeostasis (Fincher, et al., 2019). To be consistent with the theory of traditional Chinese medicine, *Cordyceps sinensis* is a famous tonic traditional Chinese medicine and mainly used to nourish the body for the weak people. Therefore, CCS might be more beneficial for the weak people due to the higher level of lipids.

**TABLE 2:** The amounts of various types of components in CCS and NCS samples by TOF-SIMS and qNMR.

Methods	Chemical components	CCS samples	NCS samples
TOF-SIMS^a^	total amino acids	9.16E-04 ± 1.27E-04	1.02E-03 ± 1.73E-04
	total monosaccharides	2.17E-03 ± 2.32E-04	2.22E-03 ± 3.53E-04
	total nucleosides	1.40E-03 ± 1.32E-04	1.52E-03 ± 1.50E-04
	total fatty acids	5.20E-03 ± 2.07E-03**	1.63E-03 ± 2.37E-04
	total glycerides	2.78E-03 ± 1.16E-03***	8.16E-04 ± 2.00E-04
	total MAGs	6.33E-04 ± 1.78E-04***	2.63E-04 ± 6.67E-05
	total DAGs	2.08E-03 ± 9.69E-04***	5.25E-04 ± 1.41E-04
	total TAGs	6.79E-05 ± 2.04E-05***	2.74E-05 ± 4.55E-06
	total glycerophospholipids	7.39E-05 ± 1.57E-05*	5.19E-05 ± 1.54E-05
	total PCs	3.70E-05 ± 8.96E-06	2.85E-05 ± 8.60E-06
	total PAs	1.38E-05 ± 2.93E-06	1.11E-05 ± 3.54E-06
	total PIs	2.32E-05 ± 8.81E-06**	1.24E-05 ± 4.46E-06
	total sphingolipids	3.84E-04 ± 1.21E-04	3.06E-04 ± 1.57E-04
	total sphingobases	2.62E-04 ± 1.11E-04	2.37E-04 ± 1.36E-04
	total Cers	2.55E-05 ± 7.27E-06**	1.51E-05 ± 6.27E-06
	total SMs	1.88E-05 ± 2.79E-06	1.52E-05 ± 1.03E-05
	total Hexcers	7.76E-05 ± 3.67E-05*	3.94E-05 ± 1.16E-05
	total sterols	5.17E-04 ± 9.32E-05	5.83E-04 ± 3.26E-04
	total cholesterols	9.82E-05 ± 2.39E-05	1.88E-04 ± 1.78E-04
	total ergosterols	4.19E-04 ± 7.11E-05	3.96E-04 ± 1.68E-04
	Mannitol	1.54E-04 ± 2.64E-05	1.50E-04 ± 2.53E-05
	Vitamin E	1.17E-05 ± 4.54E-06	1.40E-05 ± 5.78E-06
qNMR^b^ (*µ*mol/g)	total glycerides	224.10 ± 11.36***	93.62 ± 19.59
	total TAGs	138.98 ± 10.81***	25.57 ± 11.21
	total DAGs	59.72 ± 8.34***	42.12 ± 6.96
	total MAGs	25.40 ± 6.11	25.93 ± 2.83

a Data were calculated from the normalized peak areas of essential ions by PID.

b The amount of glycerides in a 1 g sample of *C. sinensis* was calculated from the calibrated IS, benzoic acid (the signal at *δ* 7.60 ppm), and normalized as micro moles. *p < 0.05, **p < 0.01, ***p < 0.001 compared with NCS group (n = 9).

### 3.4 The qNMR Validation for the Measurement of Glycerides

**FIGURE 6:**
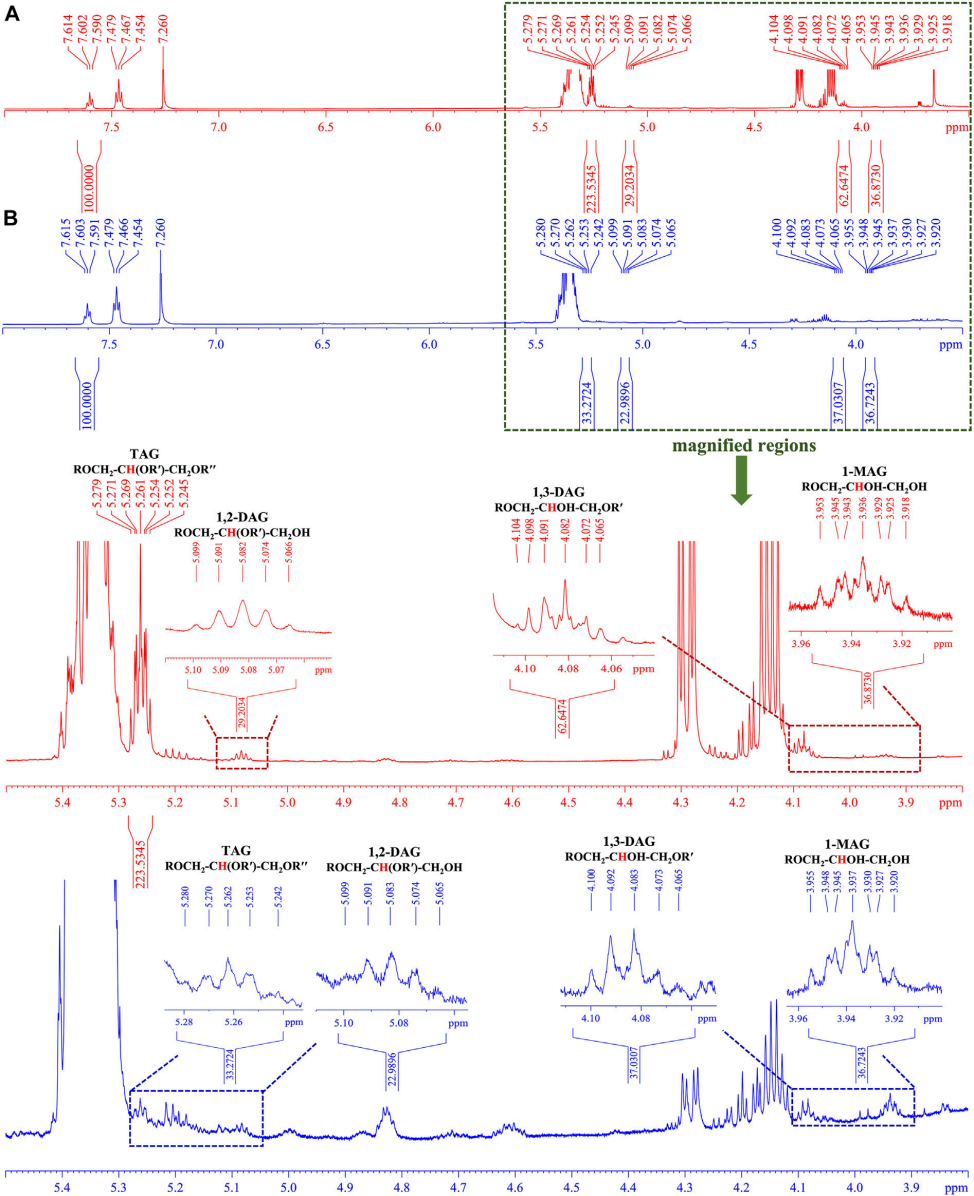
The representative ^1^H-NMR spectra (600 MHz) of cultured and natural *C. sinensis* in CDCl_3_ **(A,B)**: CCS sample (No. 2 in red) and NCS (No. 17 in blue) showing the range of chemical shift (*δ*) from 8.0 to 3.5 ppm, respectively. The tertiary protons at *sn*-2 position of detected glycerides were assigned in the magnified regions ranging from 5.5 ppm to 3.8 ppm.

Nuclear magnetic resonance (NMR) has emerged as one of the most powerful techniques for determining organic structures in complex matrices. It has a number of advantages, including the ability to simultaneously quantify multiple kinds of components when using a suitable internal standard compound. Since the tertiary proton at *sn*-2 of the glycerol backbone could be used to quantify TAGs, DAGs, and MAGs (Nieva-Echevarría et al., 2014), the glycerides in both CCS and NCS samples were thus quantified by using ^1^H-NMR in order to validate the trends of glycerides obtained by TOF-SIMS. As illustrated in Figure 6, the chemical shift (*δ*) at 5.26 ppm was assigned as the tertiary proton at *sn*-2 position in TAG [ROCH_2_-C **H** (OR′)-CH_2_OR′′], *δ* 5.08 ppm and *δ* 4.08 ppm were given by those in 1,2-DAG [ROCH_2_-C **H** (OR′)-CH_2_OH] and 1,3-DAG [ROCH_2_-C **H** OH-CH_2_OR′], respectively. The signal at *δ* 3.94 ppm was designated as the tertiary proton in 1-MAG (ROCH_2_-C **H** OH-CH_2_OH). The chemical shift at *δ* 4.08 ppm of tertiary proton at *sn*-2 of 1,3-DAG backbone was confirmed by the ^1^H-NMR spectrum of 1,3-dioleoylglycerol standard (Supplementary Figure S41). The calibrated benzoic acid was utilized as an internal standard compound, and the integral of the signal at *δ* 7.60 ppm was calibrated to 100 (Supplementary Figure S40). As the result (Table 2), the total glycerides in 1 g NCS sample were quantified to be 93.62 µmol, including 25.57 µmol TAG, 42.12 µmol DAG and 25.93 µmol 1-MAG. In sharp contrast, total glycerides in 1 g CCS sample were calculated to be 224.10 µmol, bearing 138.98 µmol TAG, 59.72 µmol DAG and 25.40 µmol 1-MAG. As a result of the remarkable contribution of TAG (*p* < 0.001) and DAG (*p* < 0.001) in CCS samples, total glycerides in CCS samples were significantly higher (*p* < 0.001) than the equivalents in NCS samples, which was consistent with the findings by TOF-SIMS. However, the tendency ​of relative contents of TAGs, DAGs and MAGs in TOF-SIMS differed from that of ^1^H-NMR, which might be due to the following factors. The TAGs were fragmented into DAG and MAG fragments due to the high energy of the primary ion beam during TOF-SIMS measurement. Similarly, DAGs were also broken into MAG pieces, as evidenced by the cleavage pattern of a 1,3-dioleoylglycerol standard compound in TOF-SIMS (Supplementary Figure S25 and Supplementary Tables S14). TAGs’ ionization efficiency may be lower than that of DAGs and MAGs due to its large molecular mass, which is a practical problem with almost all MS apparatuses. In brief, despite the fact that some compounds were easily fragmented by a high energy primary ion stream in TOF-SIMS, it was still reliable to calculate and compare the relative total content of a given class of components between homogenous samples, such as CCS and NCS samples.

### 3.5 Mass Spectrometry Imaging of Representative Components in *C. sinensis*

**FIGURE 7:**
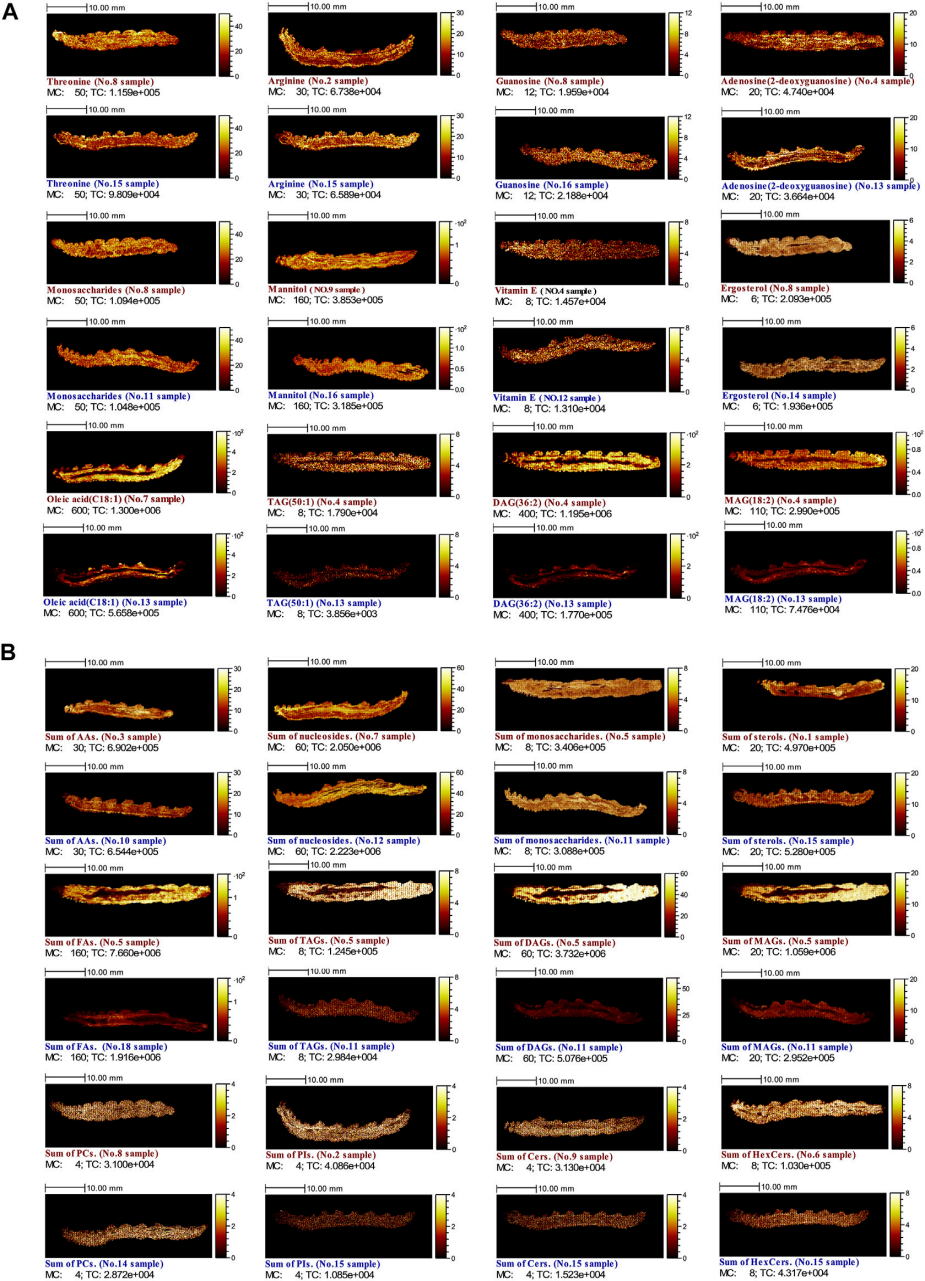
MSIs of both CCS (No. 1–9 in red) and NCS (No. 10–18 in blue) samples **(A)** Selected individual components **(B)** Total amounts of each kind of components. The MSIs of **(A)** were binned with 256 pixels. Except for the sum of TAGs, PCs, PIs, Cers, and HexCers with low intensities, which were binned independently with 64 pixels, the MSIs of **(B)** were binned with 16 pixels. MC stands for maximum counts; TC represents total counts.

MSI was utilized to visualize the chemical distribution of target ions with high lateral resolution (∼7 μm). The color scales’ amplitude was indicated as [0, mc], which corresponded to zero to the maximum number of counts (MC). For intuitive display of chemical distribution and quantitative comparison, the MSIs of the same target components in CCS and NCS samples were given the identical color thresholds. For a comprehensive MSI investigations, both individual components (Figure 7A) and the sum of a class of components (Figure 7B) were chosen, covering representative constituents of amino acids, monosaccharides, nucleosides, lipids and so on. As shown in Figure 7A, amino acids, monosaccharides, nucleosides and sterols were found to be very uniformly distributed throughout the entire body of the caterpillar in both CCS and NCS samples. However, TAGs, DAGs, MAGs, and FAs exhibited a distinctive distribution, with relatively high dispersion outside the caterpillar’s midsection, shaping the contour of the digestive chamber. The characteristic distribution of TAGs, DAGs, MAGs and FAs might be associated with the distribution of invaded fungi in the caterpillar body, and the metabolism of insect and fungi in the caterpillar body. The detected components in each homogeneous chemical category were pooled for an intuitively quantitative comparison based on MSI brightness. As illustrated in Figure 7B, no visible differences were observed in total amino acids, total nucleosides, total monosaccharides, total sterols, and total PCs between CCS and NCS samples based on the brightness of MSIs. However, TAGs, DAGs, MAGs, FAs, PIs, Cers, and HexCers were found in higher levels in CCS samples than NCS samples.

## 4 Conclusion

For cryosection sample preparation of *C. sinensis*, a double-layered media embedding approach was developed for the first time in this research, which was successfully employed for *in situ* chemical profiling and imaging of cultured and natural *C. sinensis* by using the TOF-SIMS methodology. More than 200 chemical components were simultaneously identified in the sliced CCS and NCS samples by TOF-SIMS. The identified constituents included amino acids, nucleosides, monosaccharides, fatty acids, glycerides, glycerophospholipids, sphingolipids, sterols, mannitol, myo-inositol, vitamin E, maltol and so on. Chemometric analysis was used to simplify and explain the massive TOF-SIMS data sets, showing that there was a high degree of similarity between cultured and natural *C. sinensis*. Relative quantitative analysis of TOF-SIMS data revealed that CCS has comparable proportions of major classes of chemical components to NCS, including amino acids, nucleosides, monosaccharides, sphingolipids, and sterols, except for a relatively high lipid level in fatty acids, glycerides, and glycerophospholipids. Furthermore, the higher quantity of glycerides in CCS samples than NCS samples were confirmed by quantitative ^1^H-NMR study, suggesting that TOF-SIMS method was reliable for the relative quantification of the homologous components in homogeneous samples. Meanwhile, the locations of detected ions were simultaneously recorded on samples’ surfaces by TOF-SIMS. MSIs revealed that most components, such as amino acids, monosaccharides and nucleosides, were evenly distributed throughout the whole caterpillar’s body, whereas some lipids, such as TAGs, DAGs, MAGs and FAs, showed a distinct distribution outside the caterpillar’s residual digestive chambers. To be consistent with the result of relative quantitative analysis, MSIs also intuitively demonstrated that most chemical components exhibited comparable amounts in both CCS and NCS samples. For instance, adenosine and mannitol, commonly regarded as chemical marks for quality control of *C. sinensis* (Shashidhar et al., 2013; Zhao, et al., 2014), showed similar levels of contents in CCS and NCS.

Conclusively, a new and reliable TOF-SIMS approach was firstly developed for *in situ* chemical investigation of cultured and natural *C. sinensi*s in this research. *In situ* profiling and imaging of TCMs has overcome the limitations of conventional analytical technologies which can only evaluate specific components extracted by defined solvents. Herein, diverse chemical components and their accurate locations on the surfaces of longitudinal slices of *C. sinensis* were simultaneously acquired by TOF-SIMS, and the explanations of these chemical information and spatial locations might provide us with new clues for the research of *C. sinensi*s.

## Data Availability

The raw data supporting the conclusions of this article will be made available by the authors, without undue reservation.

## Abbreviation

ADP, Adenosine 5′-diphosphate; AMP, Adenosine 5′-monophosphate; ATP, Adenosine 5′-triphosphate; CDP, Cytidine 5′-diphosphate; CCS, Cultured Cordyceps sinensis; Cer, Ceramide; CMP, Cytidine 5′-monophosphate; CMC, Carboxymethyl Cellulose; CTP, Cytidine 5′-triphosphate; *C. sinensis*/CS, Cordyceps sinensis; DAG, Diacylglycerol; FA, Fatty acid; GDP, Guanosine 5′-diphosphate; GMP, Guanosine 5′-monophosphate; GTP, Guanosine 5′-triphosphate; HCA, Hierarchical cluster analysis; HexCer, Hexosylceramide; LMIG, Liquid metal ion gun; NCS, Natural Cordyceps sinensis; MAG, Monoacylglycerol; MC, Maximum number of counts; MSI, Mass spectrometry imaging; OCT, Optimal Cutting Temperature Compound; OPLS-DA, Orthogonal partial least-squares discriminant analysis; PA, Phosphatidic acid; PC, Phosphatidylcholine; PCA, Principal component analysis; PI, Phosphatidylinositol; PID, Primary ion dose; Sa, Sphinganine; SNR: Signal-to-noise ratio; SM, Sphingomyelin; So, Sphingosine; ROI, Region of interest; TAG, Triacylglycerol; TMP, Thymidine 5′-monophosphate; TOF-SIMS, Time of Flight-Secondary Ion Mass Spectrometry; UDP, Uridine 5′-diphosphate; UMP, Uridine 5′-monophosphate; UTP, Uridine 5′-triphosphate; 2′-dADP, 2′-Deoxyadenosine 5′-diphosphate; 2′-dAMP, 2′-Deoxyadenosine 5′-monophosphate; 2′-dCDP, 2′-Deoxycytidine 5′-diphosphate; 2′-dCMP, 2′-Deoxycytidine 5′-monophosphate; 2′-dGDP, 2′-Deoxyguanosine 5′-diphosphate; 2′-dGMP, 2′-Deoxyguanosine 5′-monophosphate; ^1^H-NMR, Proton Nuclear Magnetic Resonance; qNMR, quantitative ^1^H-NMR.
