# Supplementary material for: In situ Chemical Profiling and Imaging of Cultured and Natural Cordyceps sinensis by TOF-SIMS
Source: Front Chem. 2022 Mar 24;10:862007. doi: 10.3389/fchem.2022.862007 (PMC8987775; doi:10.3389/fchem.2022.862007)
Supplement: Supplementary file 1 [file DataSheet1.PDF]

Supplementary Material

Content

1 Experimental part for monolayer medium embedding preparation 4

2 Results and discussion on monolayer medium embedding preparation 4

3 The optimized double-layer media embedding preparation 5

4 Supplementary Figures and Tables 5

Supplementary Figure S1. The integrity of representative sample solid blocks. 6

Supplementary Figure S2. The incomplete cryosection of the sample embedded in 4% CMC. 6

Supplementary Figure S3. The qualities of sample cryosections observed under a microscope with a 4x objective. 7

Supplementary Figure S4. The MSIs of total ion counts of samples in positive mode......... 7

Supplementary Figure S5. TOF-SIMS spectra demonstrating the chemical interference of OCT and 10 % gelatin on *C. sinensis* in the positive mode. 8

Supplementary Figure S6. MSIs demonstrating the overlay of matrix ions and sample components to show matrix interference on in *C. sinensis* in positive mode.......................... 9

Supplementary Figure S7. The MSIs of total ion counts of samples in negative mode........ 9

Supplementary Figure S8. TOF-SIMS spectra exhibiting the chemical interference of OCT and 10 % gelatin on *C. sinensis* in the negative mode. 10

Supplementary Figure S9. MSIs demonstrating the overlay of matrix ions and sample components to show matrix interference on in *C. sinensis* in negative mode....................... 11

Supplementary Figure S10. Evaluation of the migration of DAGs in *C. sinensis* with various sample preparation approaches by MSIs in positive mode. 12

Supplementary Figure S11. Evaluation of the chemical diffusion of FAs in *C. sinensis* with various sample preparation approaches by MSIs in negative mode..................................... 13

Supplementary Figure S12. The study on TOF-SIMS spectra of threonine standard in the positive mode... 14

Supplementary Table S1. The peak assignments of threonine standard in TOF-SIMS spectra in the positive mode. 14

Supplementary Figure S13. The study on TOF-SIMS spectra of histidine standard in the positive mode... 15

Supplementary Table S2. The peak assignments of histidine standard in TOF-SIMS spectra in the positive mode.. 15

Supplementary Figure S14. The study on TOF-SIMS spectra of phenylalanine standard in the positive mode.. 16

Supplementary Table S3. The peak assignments of phenylalanine standard in TOF-SIMS spectra in the positive mode. 16

Supplementary Figure S15. The study on TOF-SIMS spectra of adenosine standard in the positive mode.......... 17

Supplementary Table S4. The peak assignments of adenosine standard in TOF-SIMS spectra in the positive mode. 18

Supplementary Figure S16. The study on TOF-SIMS spectra of uridine standard in the positive mode.......... 19

Supplementary Table S5. The peak assignments of uridine standard in TOF-SIMS spectra in the positive mode.......... 19

Supplementary Figure S17. The study on TOF-SIMS spectra of cytidine standard in the positive mode.......... 20

Supplementary Table S6. The peak assignments of cytidine standard in TOF-SIMS spectra in the positive mode.......... 21

Supplementary Figure S18. The study on TOF-SIMS spectra of thymidine standard in the positive mode.......... 22

Supplementary Table S7. The peak assignments of thymidine standard in TOF-SIMS spectra in the positive mode. 22

Supplementary Figure S19. The study on TOF-SIMS spectra of 2-deoxyguanosine standard in the positive mode.......... 23

Supplementary Table S8. The peak assignments of 2-deoxyguanosine standard in TOF-SIMS spectra in the positive mode. 23

Supplementary Figure S20. The TOF-SIMS spectra of D-glucose standard in the positive mode. 25

Supplementary Table S9. The peak assignments of D-glucose standard in TOF-SIMS spectra in the positive mode. 25

Supplementary Figure S21. The study on TOF-SIMS spectra of D-fructose standard in the positive mode.......... 26

Supplementary Table S10. The peak assignments of D-fructose standard in TOF-SIMS spectra in the positive mode. 26

Supplementary Figure S22. The study on TOF-SIMS spectra of mannitol standard in the positive mode.......... 28

Supplementary Table S11. The peak assignments of mannitol standard in TOF-SIMS spectra in the positive mode. 28

Supplementary Figure S23. The study on TOF-SIMS spectra of cholesterol standard in the positive mode.......... 29

Supplementary Table S12. The peak assignments of cholesterol standard in TOF-SIMS spectra in the positive mode. 29

Supplementary Figure S24. The study on TOF-SIMS spectra of ergosterol standard in the positive mode. 31

Supplementary Table S13. The peak assignments of ergosterol standard in TOF-SIMS spectra in the positive mode. 31

Supplementary Figure S25. The study on TOF-SIMS spectra of 1,3-dioleoylglycerol standard in the positive mode. 33

Supplementary Table S14. The peak assignments of 1,3-dioleoylglycerol standard in TOF-SIMS spectra in the positive mode. 33

Supplementary Figure S26. The study on TOF-SIMS spectra of phosphatidylcholine standard in the positive mode. 35

Supplementary Table S15. The peak assignments of phosphatidylcholine standard in TOF-SIMS spectra in the positive mode. 35

Supplementary Figure S27. The study on TOF-SIMS spectra of threonine standard in the negative mode. 36

Supplementary Table S16. The peak assignments of threonine standard in TOF-SIMS spectra in the negative mode. 36

Supplementary Figure S28. The study on TOF-SIMS spectra of arginine standard in the negative mode. 37

Supplementary Table S17. The peak assignments of arginine standard in TOF-SIMS spectra in the negative mode. 37

Supplementary Figure S29. The study on TOF-SIMS spectra of palmitic acid standard in the negative mode. 38

Supplementary Table S18. The peak assignments of palmitic acid standard in TOF-SIMS spectra in the negative mode. 38

Supplementary Figure S30. The study on TOF-SIMS spectra of oleic acid standard in the negative mode. 40

Supplementary Table S19. The peak assignments of oleic acid standard in TOF-SIMS spectra in the negative mode. 40

Supplementary Figure S31. The study on TOF-SIMS spectra of adenosine standard in the negative mode. 41

Supplementary Table S20. The peak assignments of adenosine standard in TOF-SIMS spectra in the negative mode. 42

Supplementary Figure S32. The study on TOF-SIMS spectra of mannitol standard in the negative mode. 43

Supplementary Table S21. The peak assignments of mannitol standard in TOF-SIMS spectra in the negative mode. 43

Supplementary Figure S33. The TOF-SIMS fingerprints of *C. sinensis* in positive mode. 45

Supplementary Figure S34. The TOF-SIMS fingerprints of *C. sinensis* in negative mode. 46

Supplementary Figure S35. The magnified TOF-SIMS spectra of C. sinensis in positive mode. 47

Supplementary Figure S36. The magnified TOF-SIMS spectra of *C. sinensis* in negative mode. 48

Supplementary Figure S37. The enlarged TOF-SIMS spectra of cultured and natural *C. sinensis* in positive mode. 49

Supplementary Figure S38. The enlarged TOF-SIMS spectra of CCS and NCS samples in positive mode. 50

Supplementary Table S22. The result of similarity calculation of TOF-SIMS data. 51

Supplementary Figure S39. The 200 times of permutation test of OPLS-DA mode. 52

Supplementary Table S23. The differentially expressed components that were screened out by S-plot in positive mode. 52

Supplementary Table S24. The differentially expressed components that were screened out by S-plot in negative mode 53

Supplementary Figure S40. The representative ^{1}H-NMR spectra of *C. sinensis* with internal standard of benzoic acid in $CDCl_3$. 53

Supplementary Figure S41. The ^{1}H-NMR spectra of 1,3-dioleoylglycerol standard in $CDCl_3$. 54

1 Experimental part for monolayer medium embedding preparation

The sample preparation procedure was optimized by using a transverse section of cultured *C. sinensis* (n=3) due to the easy and simple operation compared to that of the longitudinal section. The CCS was initially wrapped in dust-free tissue moistened with MS grade water and placed in a vacuum tank for 3 hours. Under the vacuum condition, the dry samples could be restored the original shape completely in a short time. Then, the soft samples were cut into transverse sections by a stainless blade. The abdomens of CCS were chosen to evaluate the qualities of various preparation processes. Three types of embedding media, comprising OCT, 10% gelatin and 4% CMC were examined, as well as four coolants including liquid N_2, iso-pentane, pentane, and n-hexane. The solvents of isopentane, pentane, and n-hexane were pre-cooled in liquid N_2 in order to keep temperature equilibrium throughout the whole operation. The cross sections of CCS were firstly coasted by every embedding medium in the mold and then frozen by each coolant for several minutes. After that, the frozen blocks were kept for half an hour in a CM1860 cryostat (Leica Biosystems, Wetzlar, Germany) at -20°C to achieve temperature equilibrium. Subsequently, the samples were sliced into 10 μm slices and thaw-mounted on the silicon wafers and glass slides alternately.

2 Results and discussion on monolayer medium embedding preparation

The best combination of embedding medium and coolant should be satisfied with the basic requirements, including the integrity of both the frozen solid block and the cryosection of the tissue, no chemical diffusion and matrix impact on the analyzed sample cryosection. The completeness of a frozen solid block could be estimated intuitively. The integrity of the cryosection of the tissue has to be assessed under a microscope since ice crystals can damage tissues microscopically.

Furthermore, TOF-SIMS was also used to assess both the matrix effects on the sample and chemical migrations caused by different preparation methods.

Three kinds of embedding media including OCT, 4% CMC, and 10% gelatin were first examined for the sample embedding of *C. sinensis* with the use of four coolants (liquid N_2, and three pre-cooled organic solvents of iso-pentane, pentane, and n-hexane). As shown in **Supplementary Figure S1**, samples embedded in OCT and 4% CMC were obtained as solid sample blocks in all four coolants, and samples embedded in 10% gelatin were acquired as fine frozen blocks in three pre-cooled organic solvents, but broken ones in liquid N_2. This indicated that liquid N_2 was not a suitable coolant for the samples coated in 10% gelatin.

Supplementary Figure S2 showed that the frozen blocks embedded with 4% CMC were hard to obtain a complete cryosection, and 4% CMC was thus ruled out as a viable embedding medium for the sample of *C. sinensis*.

Supplementary Figure S3 presented the qualities of complete sample cryosections under a microscope. The appearances of sample cryosections revealed that sample slices embedded with 10% gelatin were easy to fold, indicating that the supportability of 10% gelatin for sample cryosection was poorer than that of OCT.

Supplementary Figure S4 - Supplementary S11 were data analysis of TOF-SIMS to further monitor the matrix effect and chemical migration on sample in both positive and negative modes. As the results, OCT had several positive dominating ions in the range with m/z value from 300 to 450 (especially two predominant positive ions with m/z 332.33 and m/z 360.36 in **Supplementary Figure S5A** and **Supplementary Figure S6A**) which might exert chemical interference on the samples. In sharp contrast, 10% gelatin exhibited the dominating ions with m/z value less than 100 in both positive and negative modes (**Supplementary Figure S5B, S6B, S8B** and **S9B**). Therefore, 10% gelatin had less matrix impact on the samples than that of OCT.

Supplementary Figure S10 and **Supplementary Figure S11** revealed that there were chemical migrations for samples frozen by both coolants of n-hexane and pentane, and no obvious migrations were observed in samples frozen by the pre-cooled iso-pentane. This finding indicated that only liquid N_2-cooled iso-pentane was a suitable coolant for both embedding media of OCT and 10% gelatin.

3 The optimized double-layer media embedding preparation

Based on the findings of single medium embedding methods, a double-layered media coating approach was devised for the sample preparation as follows. 10% gelatin was used as the first embedding medium to coat the sample, followed by the second one of OCT, and frozen by the liquid N_2-cooled iso-pentane for several minutes. The completeness of slice was examined under a microscope, and matrix effects on samples were subsequently evaluated by using TOF-SIMS in both positive and negative modes.

4 Supplementary Figures and Tables

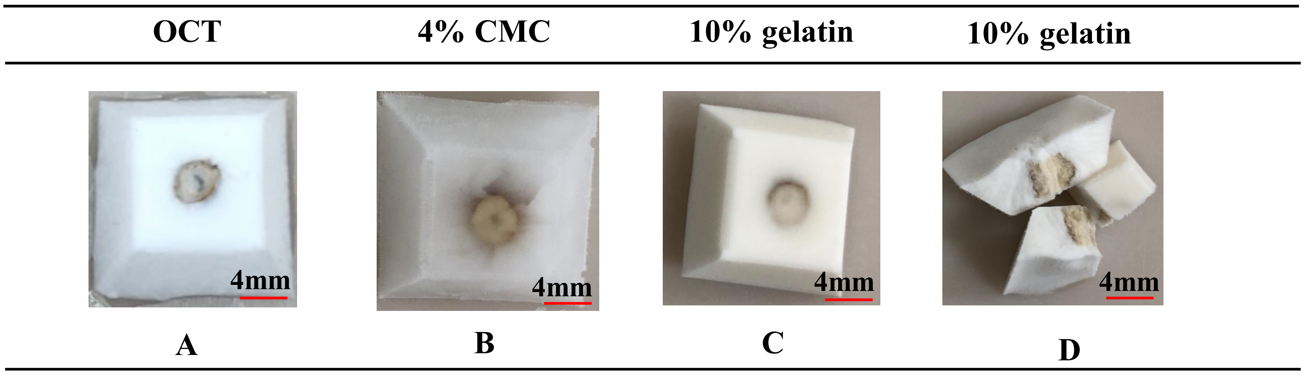

Supplementary Figure S1. The integrity of representative sample solid blocks. Liquid N_2, iso-pentane, pentane and n-hexane were used as cooling reagents. Iso-pentane, pentane and n-hexane were pre-cooled in liquid N_2 before use. The samples embedded in OCT (A), or 4% CMC (B) were obtained as complete solid blocks in all above-mentioned cooling reagents. (C, D) The samples embedded with 10% gelatin were acquired as complete solid blocks in pre-cooled organic solvents (C) but fractured ones when being cooled in liquid N_2 (D). The result indicated that liquid N_2 was not a suitable coolant for the samples coated in 10% gelatin.

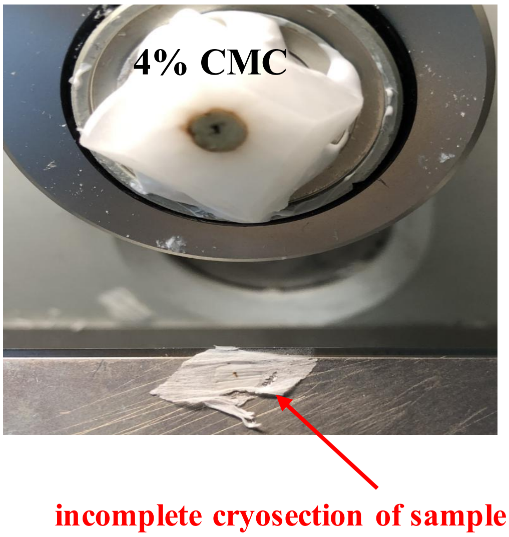

Supplementary Figure S2. The incomplete cryosection of the sample embedded in 4% CMC. It was hard to obtain a complete sliced cryosection from the frozen block embedded with 4% CMC. Therefore, 4% CMC was ruled out as a viable embedding medium for the sample of *C. sinensis*.

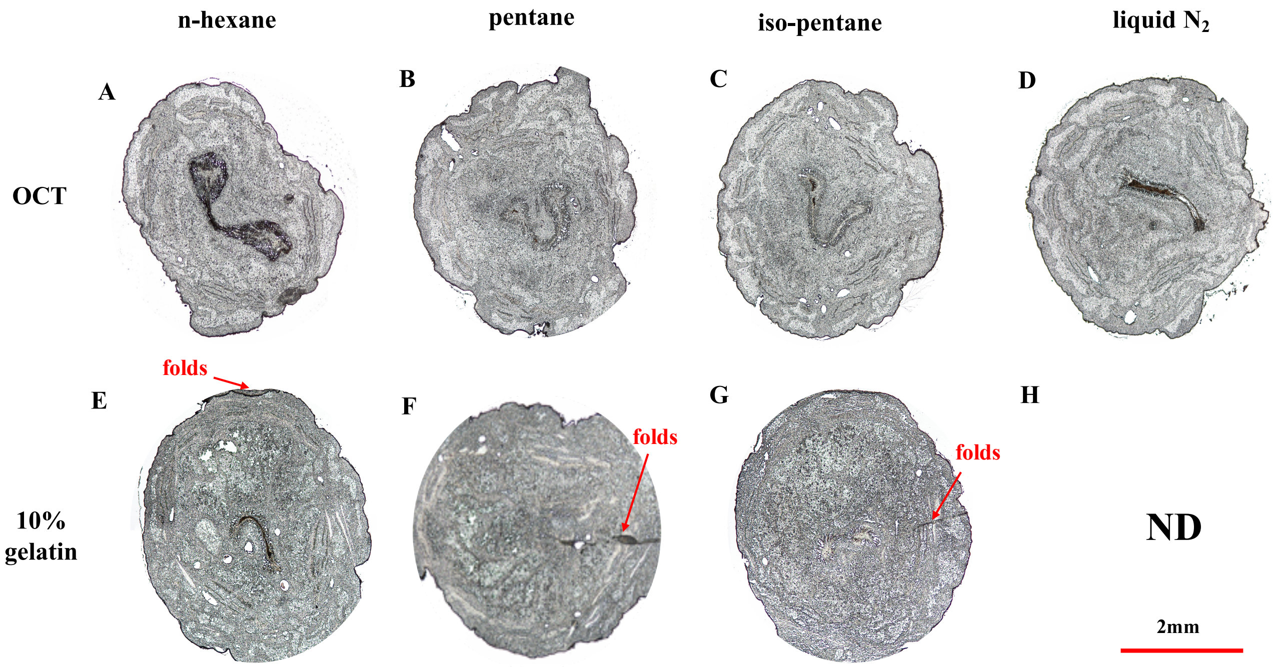

Supplementary Figure S3. The qualities of sample cryosections observed under a microscope with a 4x objective. (A, B, C, D) The samples were embedded in OCT, and (E, F, G) the samples were embedded with 10% gelatin. (A, E) The samples were frozen by pre-cooled n-hexane. (B, F) The samples were frozen by liquid N_2-cooled pentane. (C, G) The samples were frozen by liquid N_2-cooled iso-pentane. (D) The sample was frozen by liquid N2. (ND means not detected due to the broken blocks frozen in liquid N_2.) The appearances revealed that sample slices embedded with 10% gelatin were easy to fold, indicating that the supportability of 10% gelatin for sample cryosection was poorer than that of OCT.

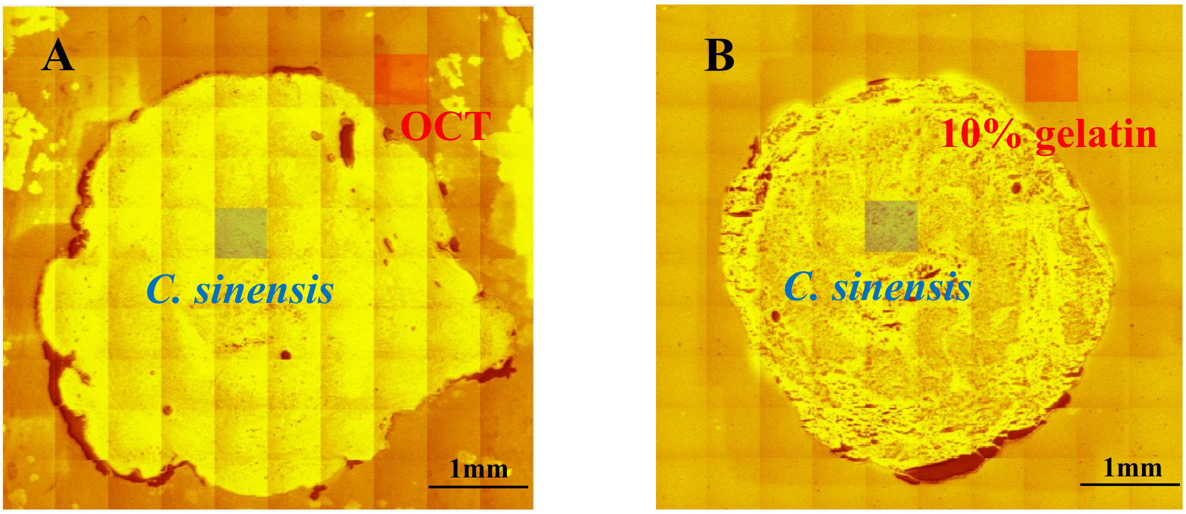

Supplementary Figure S4. The MSIs of total ion counts of samples in positive mode. (A) The image of total ion counts of the *C. sinensis* embedded in OCT with coolant of pre-cooled iso-pentane. (B) The image of total ion counts of the *C. sinensis* embedded by 10% gelatin with coolant of pre-cooled iso-pentane. The chosen regions (500 μm x 500 μm) of either OCT or 10% gelatin (red), and *C. sinensis* (blue) were separately reconstituted to study the matrix effects of OCT and 10% gelatin on *C. sinensis*.

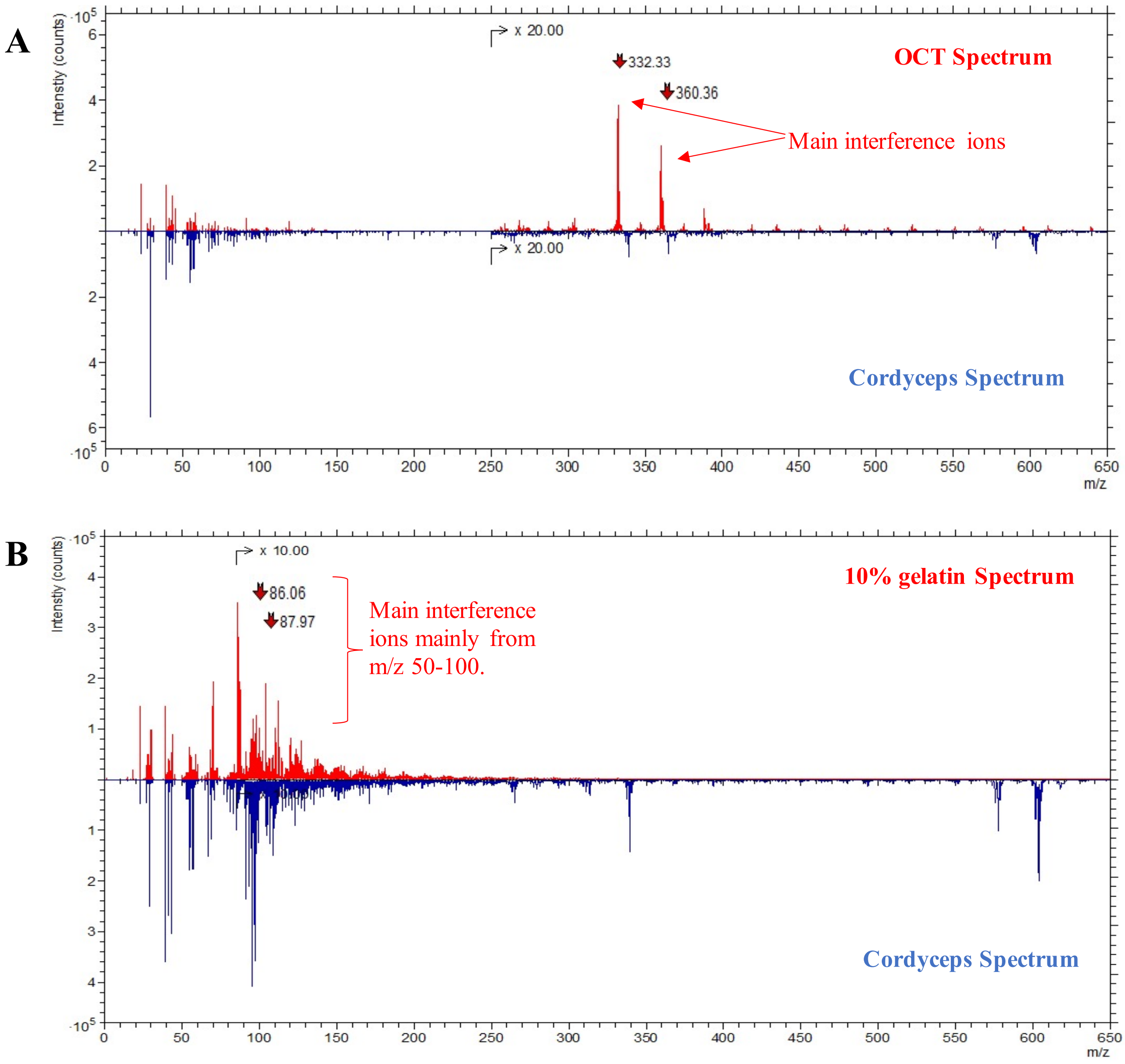

Supplementary Figure S5. TOF-SIMS spectra demonstrating the chemical interference of OCT and 10 % gelatin on *C. sinensis* in the positive mode. The spectrum in red was only reconstructed from the region of OCT (A) or 10 % gelatin (B), and the spectrum in blue was merely from *C. sinensis*. The TOF-SIMS spectrum of selected region was corresponding to that in supplementary Figure S4. The OCT spectrum showed that it had several dominating ions in the range with m/z value from 300 to 450 (especially the two predominant ions with m/z 332.33 and m/z 360.36) which might exert chemical interference on the samples. In sharp contrast, the dominating ions of 10% gelatin were mostly in the range with m/z value less than 100. It was thought that 10% gelatin had a lower matrix impact on the samples than OCT, because ions in the low *m/z* range were not employed for component identification in *C. sinensis*.

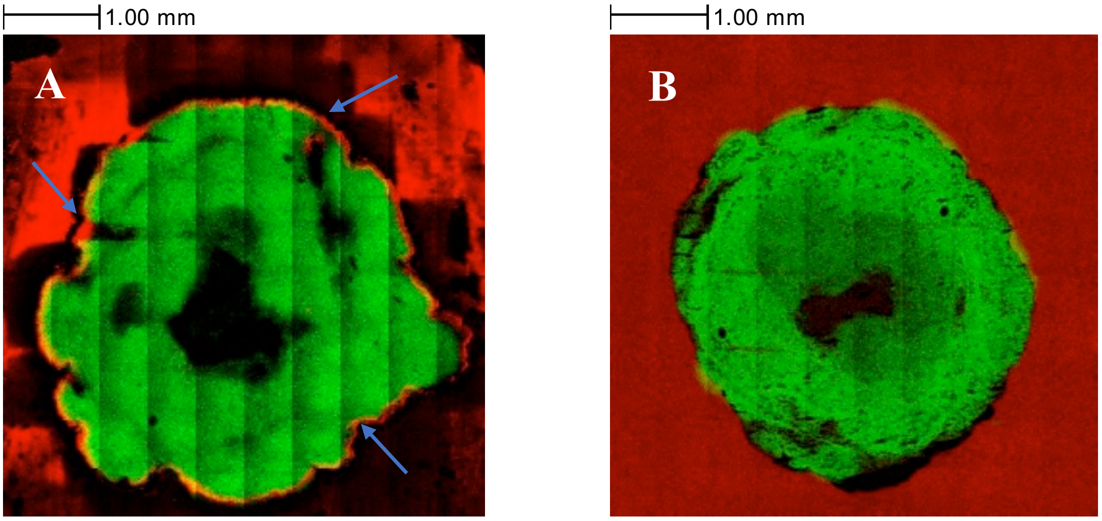

Overlay of Combined (332.33 u, 360.36 u), Combined ($C_{37}H_{69}O_4$+, $C_{39}H_{69}O_4$+, $C_{39}H_{71}O_4$+), Overlay of Combined (86.06 u, 87.97 u), Combined ($C_{37}H_{69}O_4$+, $C_{39}H_{69}O_4$+, $C_{39}H_{71}O_4$+)

Supplementary Figure S6. MSIs demonstrating the overlay of matrix ions and sample components to show matrix interference on in *C. sinensis* in positive mode. (A) The overlay of OCT's predominate ions (m/z 332.33 and 360.36 in red) and DAGs' ions (in green) in *C. sinensis*. (B) The overlay of main ions of 10% gelatin (m/z 86.06 and 87.97 in red) and DAGs' ions (in green) in *C. sinensis*. The chosen ions of DAGs were shown below the MSI's picture. Both samples were frozen in pre-cooled iso-pentane. In positive mode, the image in (A) showed that OCT easily stuck to the sample's edge (blue arrow), which might easily produce matrix interference. In sharp contrast, 10% gelatin rarely made matrix interference on the margin of the sample in positive mode due to the fact that the stickiness of 10% gelatin was poorer than that of OCT.

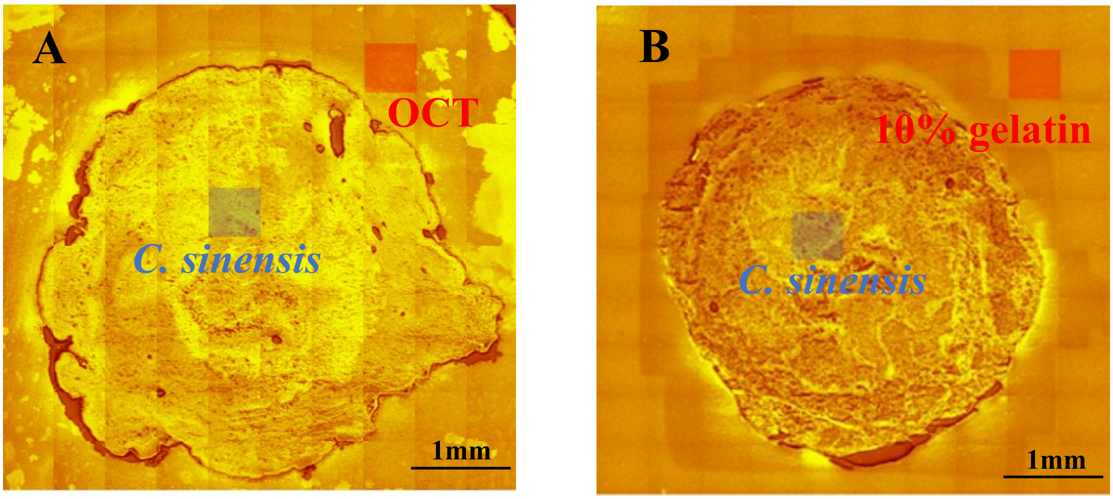

Supplementary Figure S7. The MSIs of total ion counts of samples in negative mode. (A) The image of total ion counts of the *C. sinensis* embedded with OCT with coolant of pre-cooled iso-pentane. (B) The image of total ion counts of the *C. sinensis* embedded by 10% gelatin with coolant of pre-cooled iso-pentane. The chosen regions (500 μm x 500 μm) of either OCT or 10% gelatin (in red), and *C. sinensis* (in blue) were separately recontributed to study the matrix impacts of OCT and 10% gelatin on *C. sinensis*.

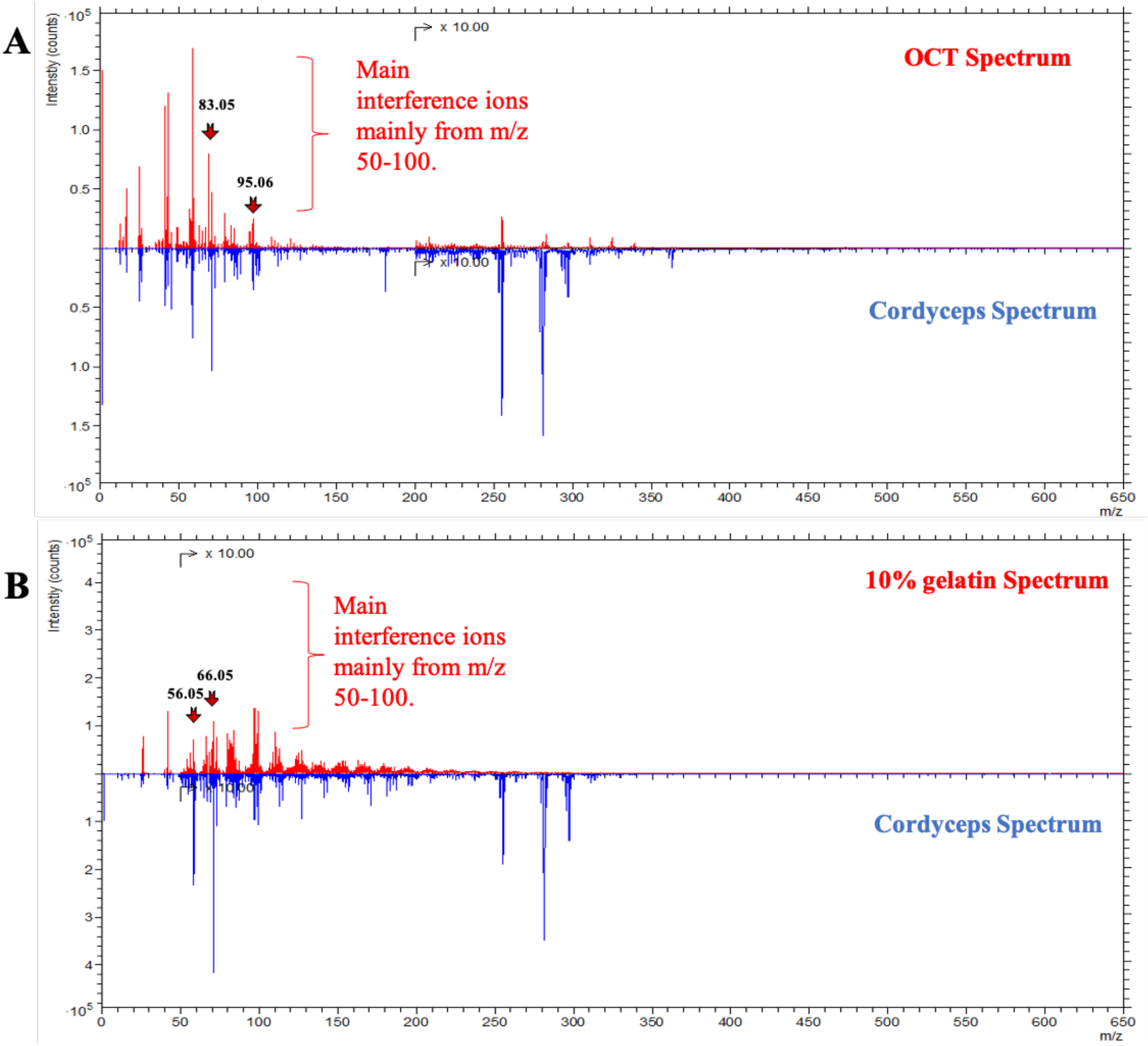

Supplementary Figure S8. TOF-SIMS spectra exhibiting the chemical interference of OCT and 10 % gelatin on *C. sinensis* in the negative mode. The spectrum (in red) was only generated from the region of OCT (A) or 10 % gelatin (B), and the spectrum (in blue) was merely from *C. sinensis*. The TOF-SIMS spectrum of selected region was corresponding to that in supplementary Figure S7. The result exhibited that the dominating ions of both OCT or 10% gelatin were mostly in the range with m/z value less than 100 in the negative mode, which might have less matrix impact on the samples.

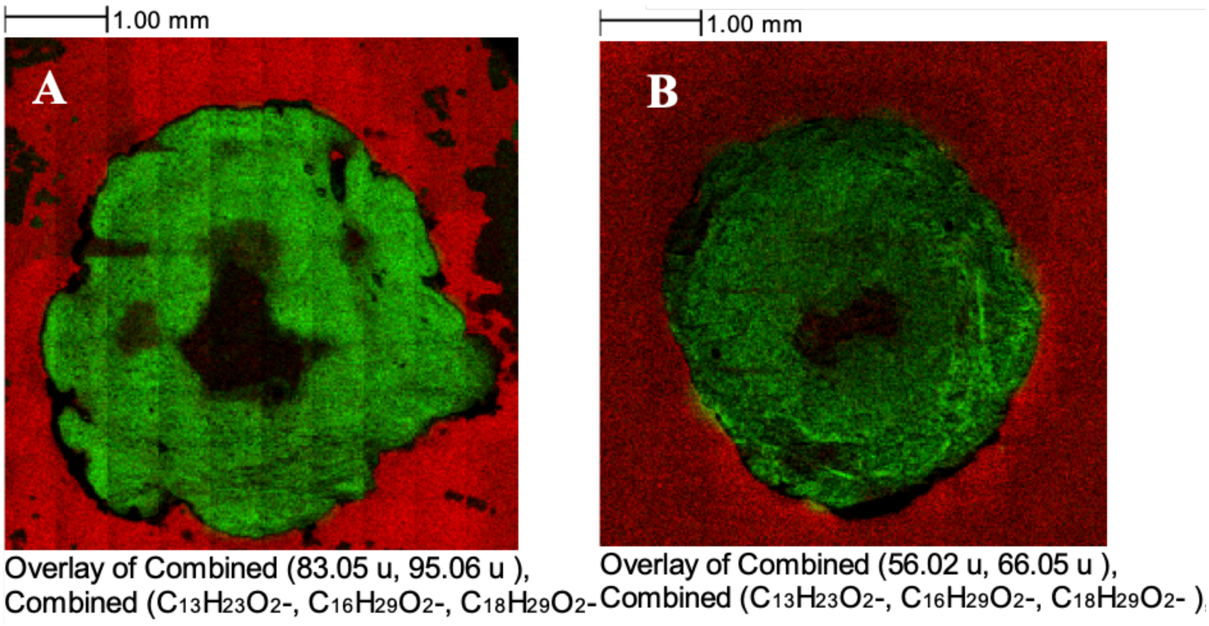

Supplementary Figure S9. MSIs demonstrating the overlay of matrix ions and sample components to show matrix interference on in *C. sinensis* in negative mode. (A) The overlay of predominant ions of OCT (m/z 83.05 and 95.06 in red) and essential ions of FAs (in green) in *C. sinensis*. (B) The overlay of main ions of 10% gelatin (m/z 56.02 and 66.05 in red) and essential ions of FAs (in green) in *C. sinensis*. The chosen ions of FAs were displayed below the pictures. Both samples were frozen in pre-cooled iso-pentane. In the negative mode, both OCT and 10% gelatin scarcely caused matrix interference according to the above results.

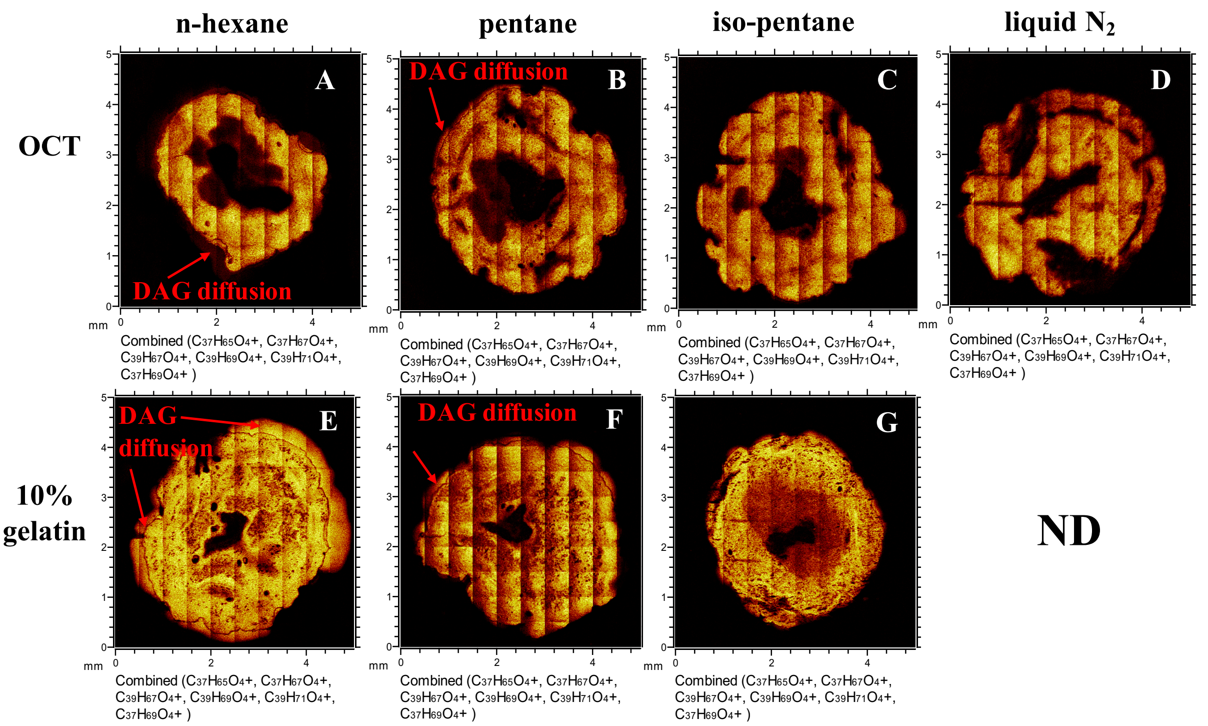

Supplementary Figure S10. Evaluation of the migration of DAGs in *C. sinensis* with various sample preparation approaches by MSIs in positive mode. (A, B, C, D) The samples were embedded by OCT and (E, F, G) the samples were embedded by 10 % gelatin. (A, E) The samples were frozen by pre-cooled n-hexane. (B, F) The samples were frozen by liquid N_2-cooled pentane. (C, G) The samples were frozen by liquid N_2-cooled iso-pentane. (D) The sample was frozen by liquid N2. (ND means not detected due to the broken blocks frozen in liquid N_2.). As the results, DAGs in samples (A, B, E, F) frozen in either n-hexane or pentane tended to migrate (red arrows), especially in samples (E, F) embedded with 10% gelatin. The above chemical migration might be attributed to the different freezing point of coolants and the physical properties of embedding media. DAGs didn't migration in samples (C, D, G) frozen with pre-cooled iso-pentane and liquid N_2, indicating iso-pentane was a suitable coolant for both embedding media of OCT and 10% gelatin.

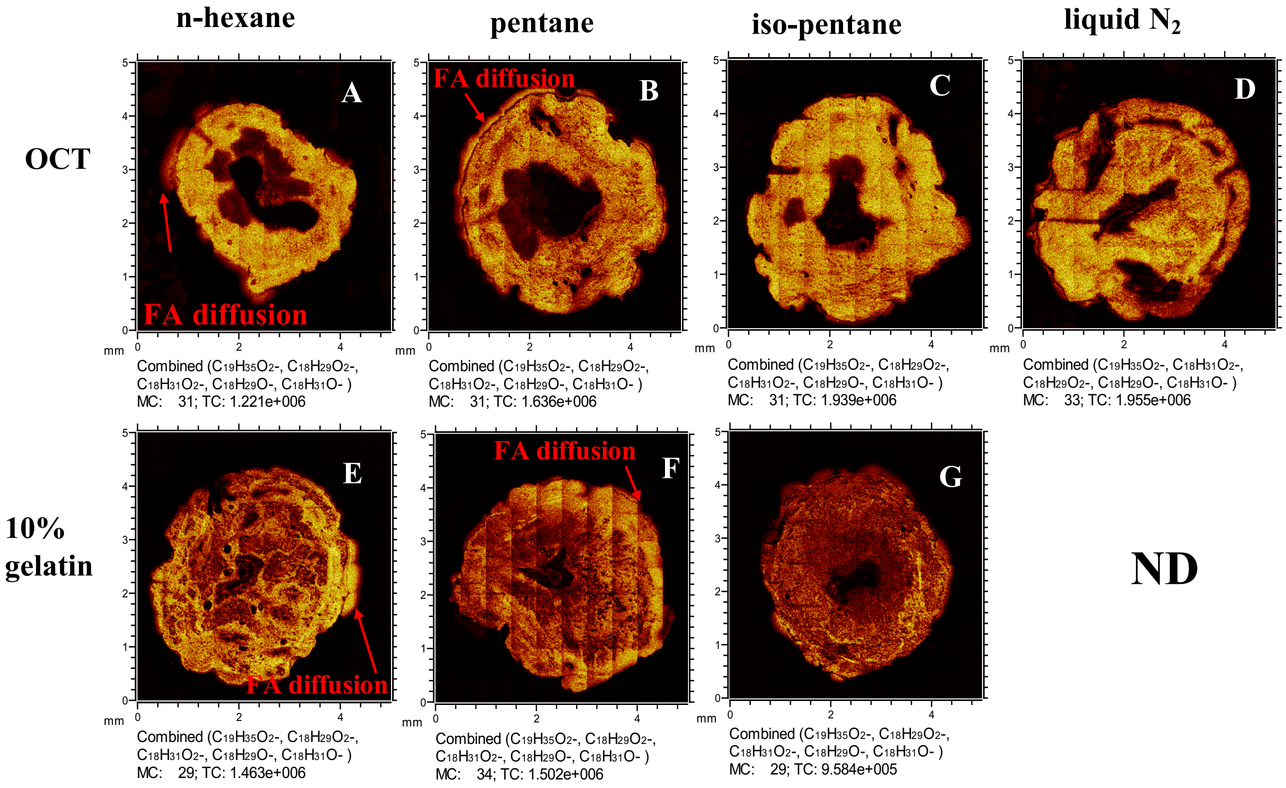

Supplementary Figure S11. Evaluation of the chemical diffusion of FAs in *C. sinensis* with various sample preparation approaches by MSIs in negative mode. (A, B, C, D) The samples were embedded with OCT and (E, F, G) the samples were embedded with 10% gelatin. (A, E) The samples were frozen by pre-cooled n-hexane. (B, F) The samples were frozen by liquid N_2-cooled pentane. (C, G) The samples were frozen by liquid N_2-cooled iso-pentane. (D) The sample was frozen by liquid N2. (ND means not detected due to the broken blocks frozen in liquid N_2.). FAs in samples (A, B, E, F) frozen in either n-hexane or pentane were found to migrate (red arrows), while no obvious migration of FAs was observed in samples (C, D, G) frozen in pre-cooled iso-pentane and liquid N_2. Iso-pentane was a suitable coolant for both embedding media of OCT and 10% gelatin.

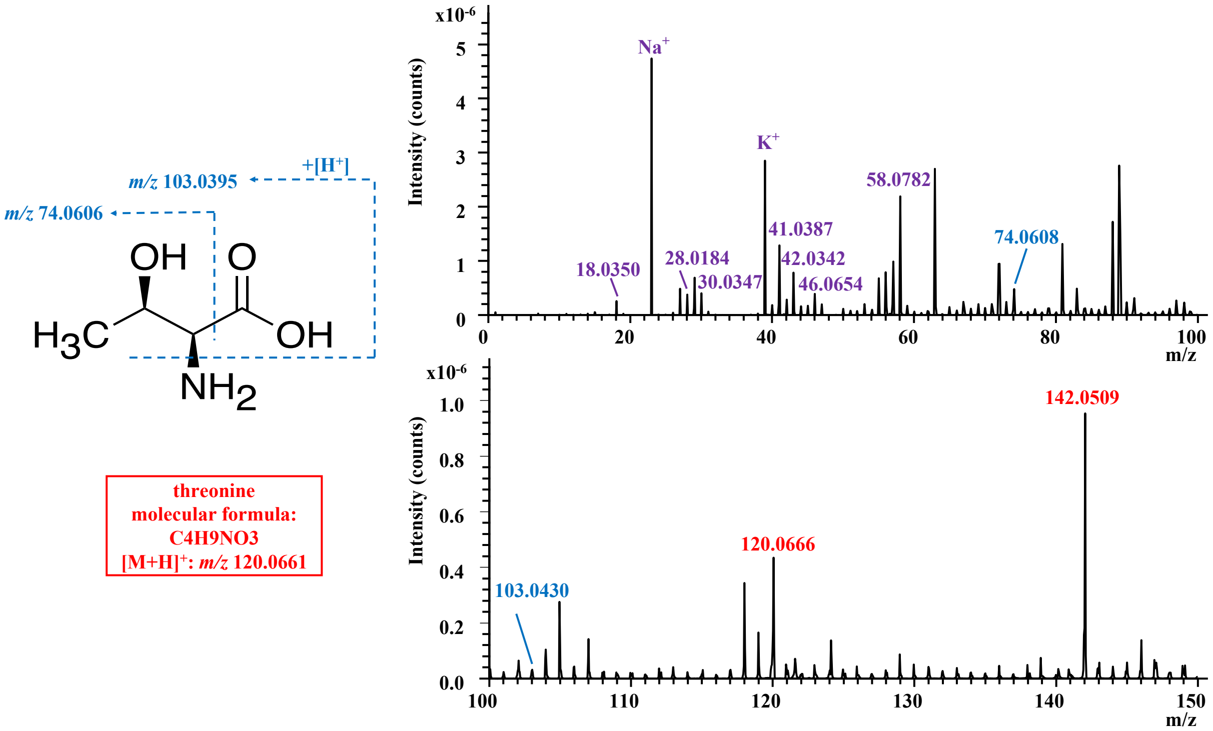

Supplementary Figure S12. The study on TOF-SIMS spectra of threonine standard in the positive mode.

Supplementary Table S1. The peak assignments of threonine standard in TOF-SIMS spectra in the positive mode.

No.	Observed mass (*m/z*)	Chemical formula	Deviation (ppm)	Species
		required peaks		
1	120.0666	$C4H10NO3^+$	8.6	$[M+H]^+$
2	142.0509	$C4H9NO3Na^+$	24.1	$[M+Na]^+$
		characteristic peaks		
3	103.0430	$C4H7O3^+$	37.8	$[M-(NH3)+H]^+$
4	74.0608	$C3H8NO^+$	10.6	$[M-COOH]^+$
		other peaks		
5	58.0782	$C3H8N^+$	133.8	-
6	46.0654	$C2H8N^+$	6.6	-
7	42.0342	$C2H4N^+$	8.8	-
8	41.0387	$C3H5^+$	3.4	-
9	30.0347	$CH4N^+$	28.7	-
10	28.0184	$CH2N^+$	8.1	-

11	18.0350	NH4$^+$	64.1	-

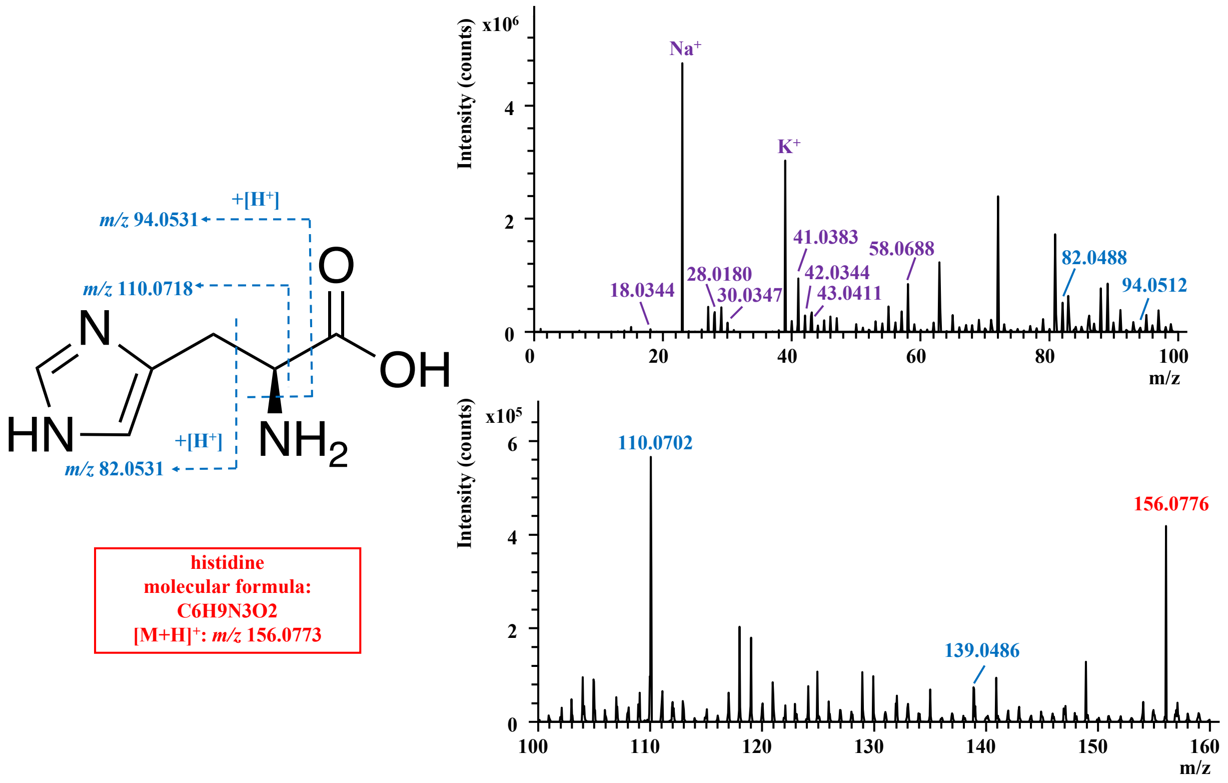

Supplementary Figure S13. The study on TOF-SIMS spectra of histidine standard in the positive mode.

Supplementary Table S2. The peak assignments of histidine standard in TOF-SIMS spectra in the positive mode.

No.	Observed mass (*m/z*)	Chemical formula	Deviation (ppm)	Species
		required peaks		
1	156.0776	C6H10N3O2$^+$	5.5	[M+H]+
		characteristic peaks		
2	139.0486	C6H7N2O2$^+$	-11.8	[M-(NH3)+H]$^+$
3	110.0702	C5H8N3$^+$	-9.6	[M-(COOH)]$^+$
4	94.0512	C5H6N2$^+$	-13.9	[M-(NH3)-(COOH)+H]$^+$
5	82.0488	C4H6N2$^+$	-45.6	[M-(CHNH2)-(COOH)+H]$^+$
		other peaks		
6	58.0688	C3H8N$^+$	64.0	-
7	43.0411	C2H5N$^+$	-13.1	-
8	42.0344	C2H4N$^+$	14.3	-

9	41.0383	$C3H5^+$	-6.9	-
10	30.0347	$CH4N^+$	28.7	-
11	28.0180	$CH2N^+$	-7.8	-
12	18.0344	$NH4^+$	33.1	-

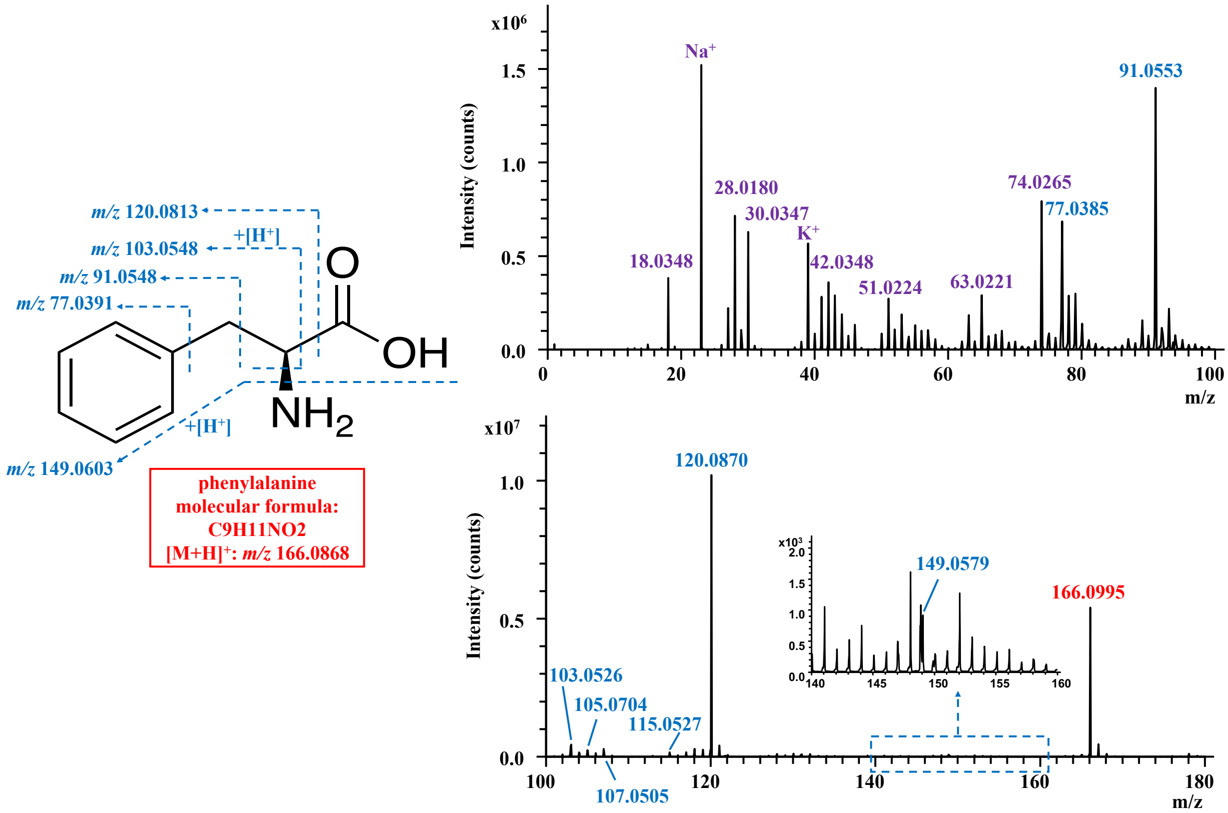

Supplementary Figure S14. The study on TOF-SIMS spectra of phenylalanine standard in the positive mode.

Supplementary Table S3. The peak assignments of phenylalanine standard in TOF-SIMS spectra in the positive mode.

No.	Observed mass (*m/z*)	Chemical formula	Deviation (ppm)	Species
		required peaks		
1	166.0995	$C9H12NO2^+$	3.8	$[M+H]^+$
		characteristic peaks		
2	149.0579	$C9H9O2^+$	-11.9	$[M-(NH3)+H]^+$
3	120.0870	$C8H10N^+$	19.4	$[M-(COOH)]^+$
4	103.0526	$C8H7^+$	-16.1	$[M-(NH3)-(COOH)]^+$
5	115.0527	$C5H9NO2^+$	-87.3	-
6	107.0505	$C7H7O^+$	12.7	-

7	105.0704	$C8H9^+$	5.5	-
8	91.0553	$C7H7^+$	11.5	$[M-(CHNH2)-(COOH)]^+$
9	77.0385	$C6H5^+$	-1.6	$[M-(CH2)-(CHNH2)-(COOH)]^+$
		other peaks		
10	74.0265	$C2H4NO2^+$	38.8	-
11	63.0221	$C5H3^+$	-13.7	-
12	51.0224	$C4H3^+$	-11	-
13	42.0348	$C2H4N^+$	24.1	-
14	30.0351	$CH4N^+$	41.9	-
15	28.0192	$CH2N^+$	34.8	-
16	18.0348	$NH4^+$	51.8	-

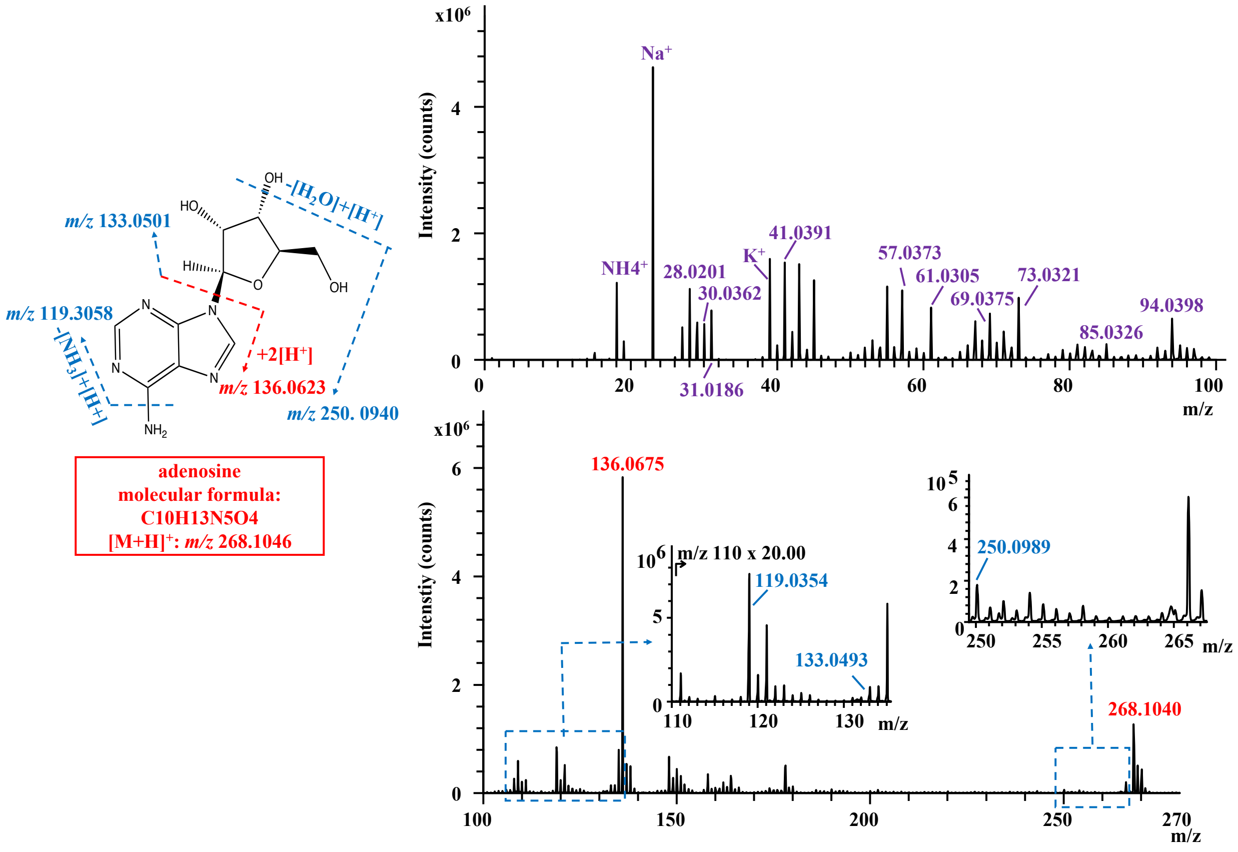

Supplementary Figure S15. The study on TOF-SIMS spectra of adenosine standard in the positive mode.

Supplementary Table S4. The peak assignments of adenosine standard in TOF-SIMS spectra in the positive mode.

No.	Observed mass (*m/z*)	Chemical formula	Deviation (ppm)	Species
		required peaks		
1	268.1040	$C10H14N5O4^{+}$	-0.2	$[M+H]^{+}$
		characteristic peaks		
2	250.0989	$C10H12N5O3^{+}$	21.7	$[M-(H_2O)+H]^{+}$
3	136.0675	$C5H6N5^{+}$	42.1	$[M(adenine)+H]^{+}$
4	133.0493	$C5H9O4^{+}$	-1.5	$[M(D\text{-}ribose)+H]^{+}$
5	119.0354	$C5H3N4^{+}$	1.4	$[M(adenine)-(NH_3)+H]^{+}$
		other peaks		
6	94.0398	$C4H4N3^{+}$	-2.4	-
7	85.0326	$C4H5O2^{+}$	49.1	-
8	73.0321	$C3H5O2^{+}$	50.8	-
9	69.0375	$C4H5O^{+}$	58.5	-
10	61.0305	$C2H5O2^{+}$	34.3	-
11	57.0373	$C3H5O^{+}$	67.1	-
12	41.0391	$C3H5^{+}$	12.5	-
13	31.0186	$CH3O^{+}$	24.8	-
14	30.0362	$CH4N^{+}$	78.5	-
15	28.0201	$CH2N^{+}$	68.7	-
16	18.0435	$NH4^{+}$	537.2	-

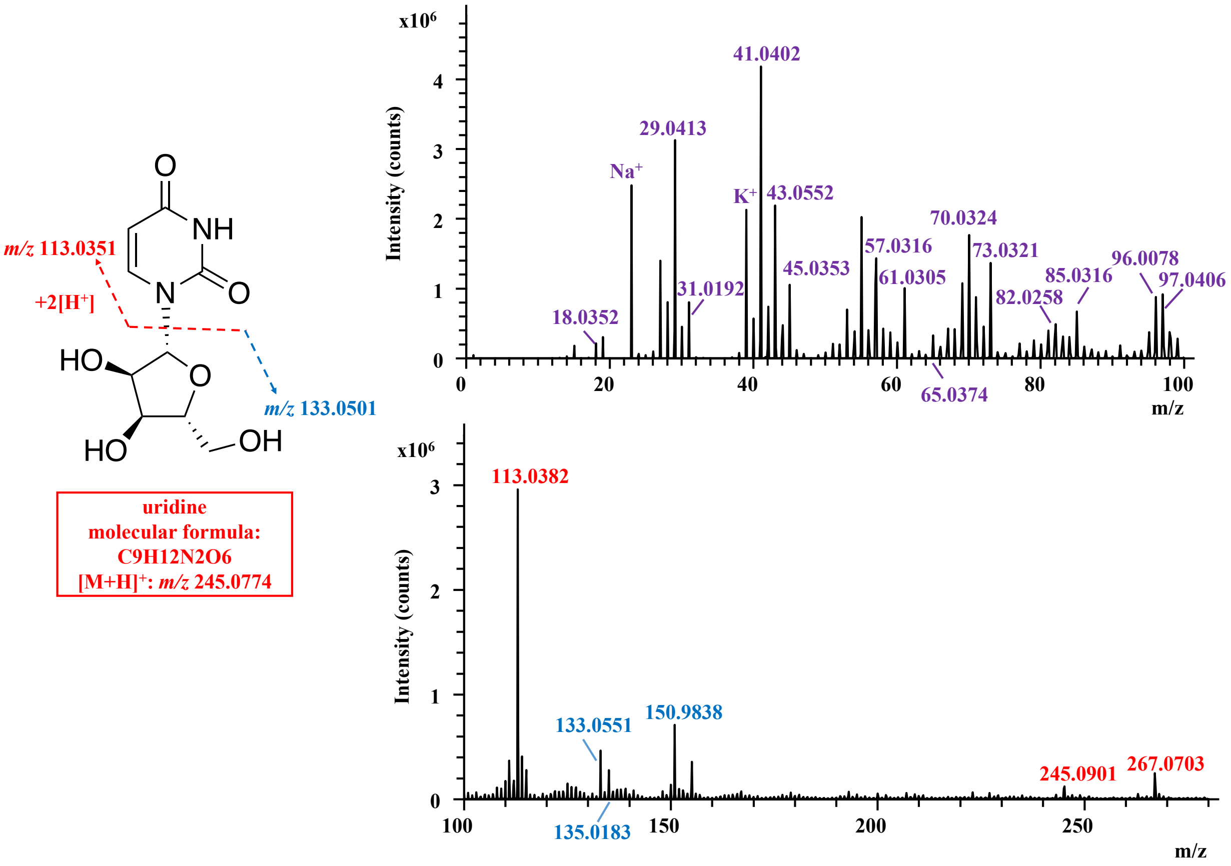

Supplementary Figure S16. The study on TOF-SIMS spectra of uridine standard in the positive mode.

Supplementary Table S5. The peak assignments of uridine standard in TOF-SIMS spectra in the positive mode.

No.	Observed mass (*m/z*)	Chemical formula	Deviation (ppm)	Species
		required peaks		
1	245.0901	$C9H13N2O6^{+}$	54.1	$[M+H]^{+}$
2	267.0703	C9H12N2O6Na+	43.1	$[M(uridine)+Na]^{+}$
		characteristic peaks		
3	150.9838	$C4H4N2O2K^{+}$	-43.8	$[M(uracil)+K]^{+}$
4	135.0183	$C4H4N2O2Na^{+}$	13.3	$[M(uracil)+Na]^{+}$
5	133.0551	$C5H9O4^{+}$	41.5	$[M(D\text{-}ribose)+H]^{+}$
6	113.0382	$C4H5N2O2^{+}$	32.2	$[M(uracil)+H]^{+}$
		other peaks		
7	97.0406	$C4H5N2O^{+}$	9.5	-
8	96.0078	$C4H2NO2^{+}$	-2.0	-

9	85.0316	$C4H5O2^+$	38.0	-
10	82.0258	$C4H4NO^+$	-35.5	-
11	73.0321	$C3H5O2^+$	50.8	-
12	70.0324	$C3H4NO^+$	52.0	-
13	61.0305	$C2H5O2^+$	34.7	-
14	57.0316	$C3H5O^+$	-33.9	-
15	45.0353	$C2H5O^+$	41.2	-
16	43.0552	$C3H7^+$	22.0	-
17	57.0316	$C3H5O^+$	-33.9	-
18	41.0402	$C3H5^+$	40.4	-
19	31.0192	$CH3O^+$	43.7	-
20	30.0354	$CH4N^+$	52.7	-
21	29.0413	$C2H5^+$	92.4	-
22	18.0352	$NH4^+$	78.1	-

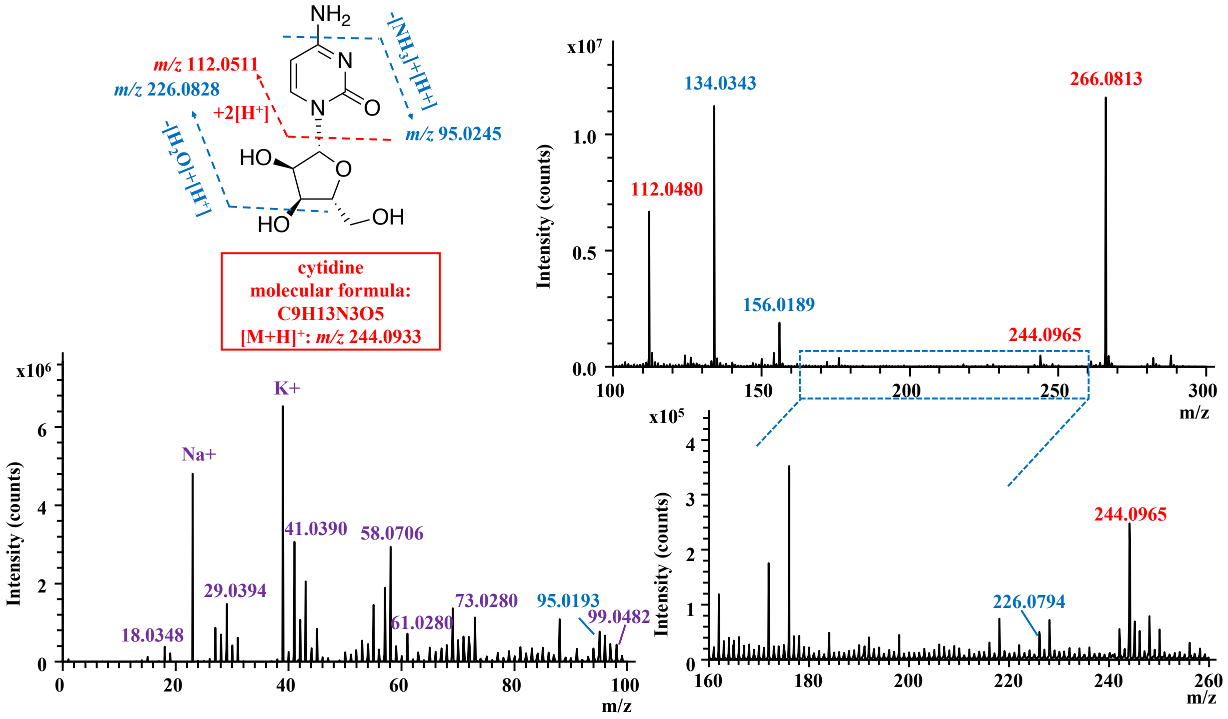

Supplementary Figure S17. The study on TOF-SIMS spectra of cytidine standard in the positive mode.

Supplementary Table S6. The peak assignments of cytidine standard in TOF-SIMS spectra in the positive mode.

No.	Observed mass (*m/z*)	Chemical formula	Deviation (ppm)	Species
		required peaks		
1	244.0965	$C9H14N3O5^{+}$	15.4	$[M+H]^{+}$
2	266.0813	$C9H13N3O5Na^{+}$	24.7	$[M+Na]^{+}$
		characteristic peaks		
3	226.0794	$C9H12N3O4^{+}$	-12.5	$[M-(H2O)+H]^{+}$
4	156.0189	$C4H4N3ONa2^{+}$	29.0	$[M(cytosine)-H+2Na]^{+}$
5	134.0343	$C4H5N3ONa^{+}$	13.3	$[M(cytosine)+Na]^{+}$
6	112.0480	$C4H6N3O^{+}$	-22.5	$[M(cytosine)+H]^{+}$
7	95.0193	$C4H3N2O^{+}$	-49.5	$[M(cytosine)-(NH3)+H]^{+}$
		other peaks		
8	99.0482	$C5H7O2^{+}$	41.4	-
9	73.0280	$C3H5O2^{+}$	-5.2	-
10	61.0280	$C2H5O2^{+}$	-5.8	-
11	41.0390	$C3H5^{+}$	9.2	-
12	31.0186	$CH3O^{+}$	23.4	-
13	30.0355	$CH4N^{+}$	55.3	-
14	29.0394	$C2H5^{+}$	28.9	-
15	28.0189	$CH2N^{+}$	24.8	-
16	18.0353	$NH4^{+}$	81.4	-

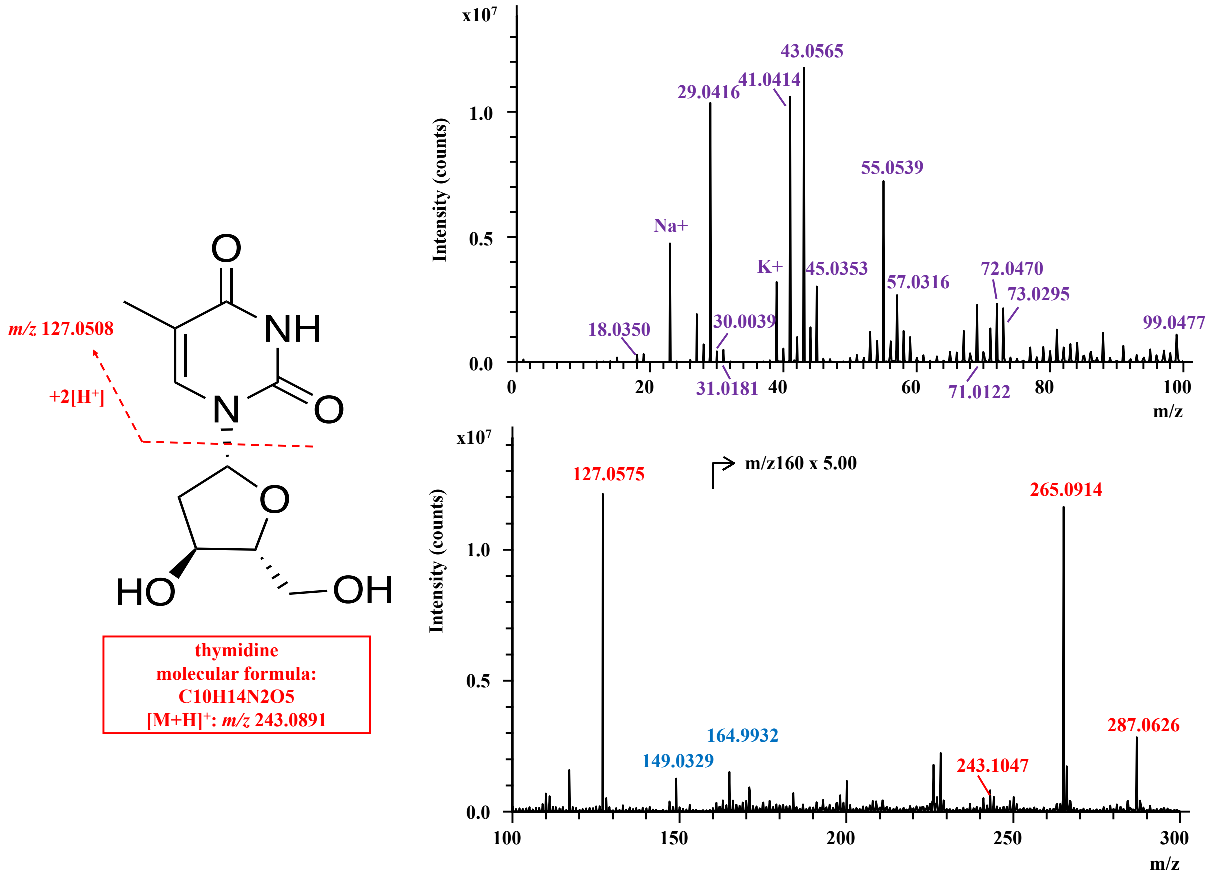

Supplementary Figure S18. The study on TOF-SIMS spectra of thymidine standard in the positive mode.

Supplementary Table S7. The peak assignments of thymidine standard in TOF-SIMS spectra in the positive mode.

No.	Observed mass (*m/z*)	Chemical formula	Deviation (ppm)	Species
		required peaks		
1	243.1047	$C10H15N2O5^{+}$	29.6	$[M+H]^{+}$
2	265.0914	$C10H14N2O5Na^{+}$	44.8	$[M+Na]^{+}$
3	287.0626	$C10H13N2O5Na2^{+}$	4.0	$[M-H+2Na]^{+}$
		characteristic peaks		
4	164.9932	$C5H6N2O2K^{+}$	-78.3	$[M(thymine)+K]^{+}$
5	149.0329	$C5H6N2O2Na^{+}$	5.3	$[M(thymine)+Na]^{+}$
6	127.0575	$C5H7N2O2^{+}$	57.7	$[M(thymine)+H]^{+}$
		other peaks		
7	99.0477	$C5H7O2^{+}$	37.0	-
8	73.0295	$C3H5O2^{+}$	14.6	-

9	72.0470	$C3H6NO^+$	36.1	-
10	71.0122	$C3H3O2^+$	-8.3	-
11	57.0316	$C3H5O^+$	-33.9	-
12	55.0539	$C4H7^+$	-5.3	-
13	45.0357	$C2H5O^+$	50.1	-
14	43.0565	$C3H7^+$	52.4	-
15	41.0409	$C3H5^+$	56.1	-
16	31.0181	$CH3O^+$	6.8	-
17	30.0339	$CH4N^+$	1.9	-
18	29.0416	$C2H5^+$	104.1	-
19	18.0345	$NH4^+$	39.5	-

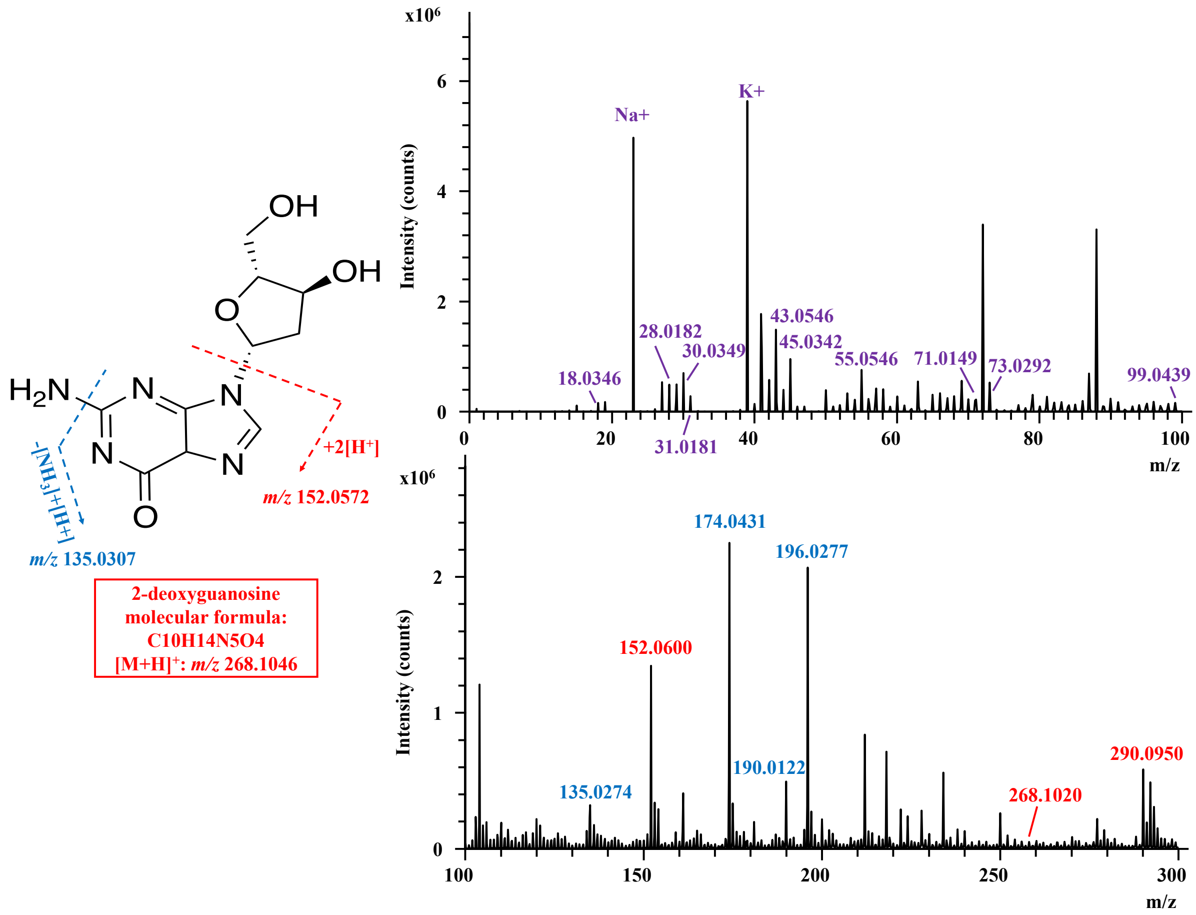

Supplementary Figure S19. The study on TOF-SIMS spectra of 2-deoxyguanosine standard in the positive mode.

Supplementary Table S8. The peak assignments of 2-deoxyguanosine standard in TOF-SIMS spectra in the positive mode.

No.	Observed mass (*m/z*)	Chemical formula	Deviation (ppm)	Species
		required peaks		
1	268.1020	$C10H14N5O4^{+}$	-7.5	$[M+H]^{+}$
2	290.0950	$C10H13N5O4Na^{+}$	31.0	$[M+Na]^{+}$
		characteristic peaks		
3	196.0277	$C5H4N5ONa2^{+}$	36.1	$[M(guanine)-H+2Na]^{+}$
4	190.0122	$C5H5N5OK^{+}$	-2.2	$[M(guanine)+K]^{+}$
5	174.0431	$C5H5N5ONa^{+}$	25.5	$[M(guanine)+Na]^{+}$
6	152.0600	$C5H6N5O^{+}$	21.6	$[M(guanine)+H]^{+}$
7	135.0274	$C5H3N4O^{+}$	-20.3	$[M(guanine)-(NH3)+H]^{+}$
		other peaks		
8	99.0439	$C5H7O2^{+}$	-1.4	-
9	73.0292	$C3H5O2^{+}$	11.5	-
10	71.0149	$C3H3O2^{+}$	2.8	-
11	55.0546	$C4H7^{+}$	6.9	-
12	45.0342	$C2H5O^{+}$	15.8	-
13	43.0546	$C3H7^{+}$	9.8	-
14	31.0181	$CH3O^{+}$	9.3	-
15	30.0349	$CH4N^{+}$	35.1	-
16	28.0182	$CH2N^{+}$	1.0	-
17	18.0349	$NH4^{+}$	10.7	-

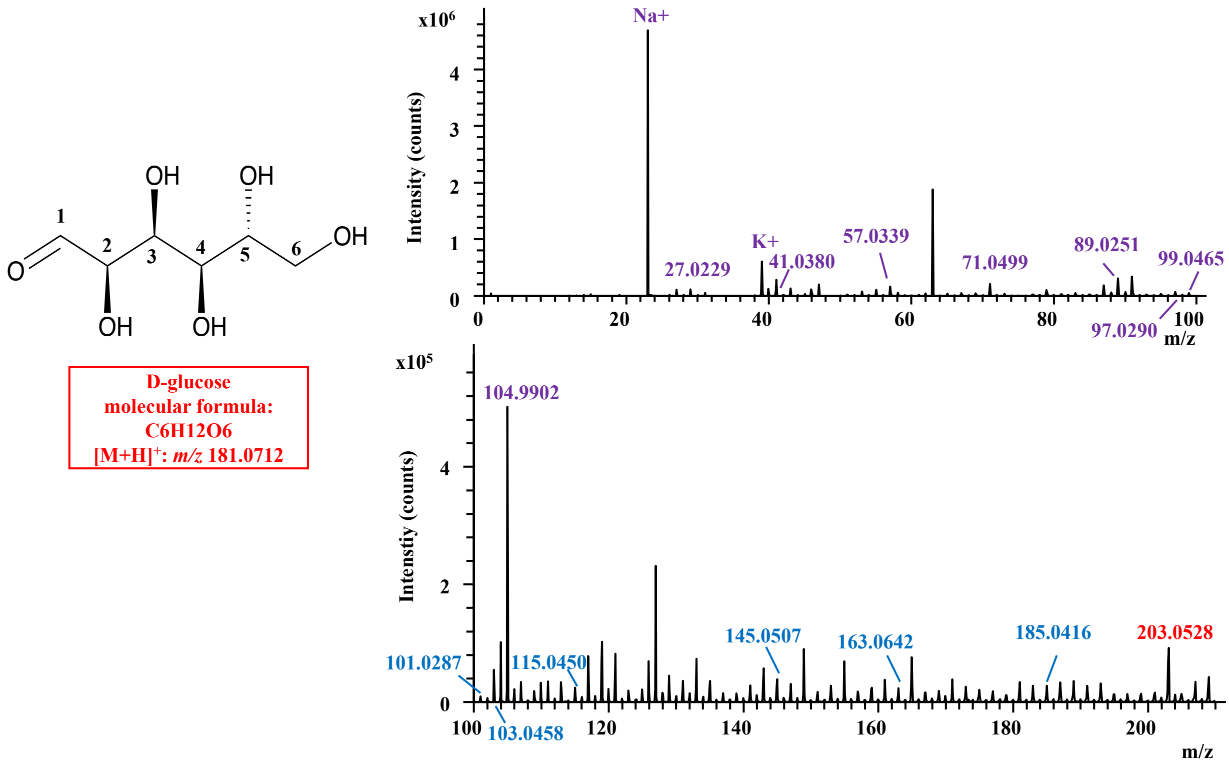

Supplementary Figure S20. The TOF-SIMS spectra of D-glucose standard in the positive mode.

Supplementary Table S9. The peak assignments of D-glucose standard in TOF-SIMS spectra in the positive mode.

No.	Observed mass (*m/z*)	Chemical formula	Deviation (ppm)	Species
		required peaks		
1	203.0564	$C6H12O6Na^+$	18.8	$[M+Na]^+$
		characteristic peaks		
2	185.0416	$C6H10O5Na^+$	-2.3	$[M-(H_2O)+Na]^+$
3	163.0642	$C6H11O5^+$	24.9	$[M-(H_2O)+H]^+$
4	145.0507	$C6H9O4^+$	8.2	$[M-2(H_2O)+H]^+$
5	127.0379	$C6H7O3^+$	-8.5	$[M-3(H2O)+H]^+$
6	115.0450	$C5H7O3^+$	52.2	-
7	103.0458	$C4H7O3^+$	66.7	-
8	101.0287	$C4H5O3^+$	53.0	-
		other peaks		
9	104.9902	$C2H3O2Na2^+$	-20.3	-
10	99.0465	$C5H7O2^+$	24.7	-

11	97.0290	$C5H5O2^{+}$	6.0	-
12	89.0251	$C3H5O3^{+}$	20.2	-
13	71.0499	$C4H7O^{+}$	11.2	-
14	57.0343	$C3H5O^{+}$	13.9	-
15	41.0399	$C3H5^{+}$	31.8	-
16	27.0233	$C2H3^{+}$	12.4	-

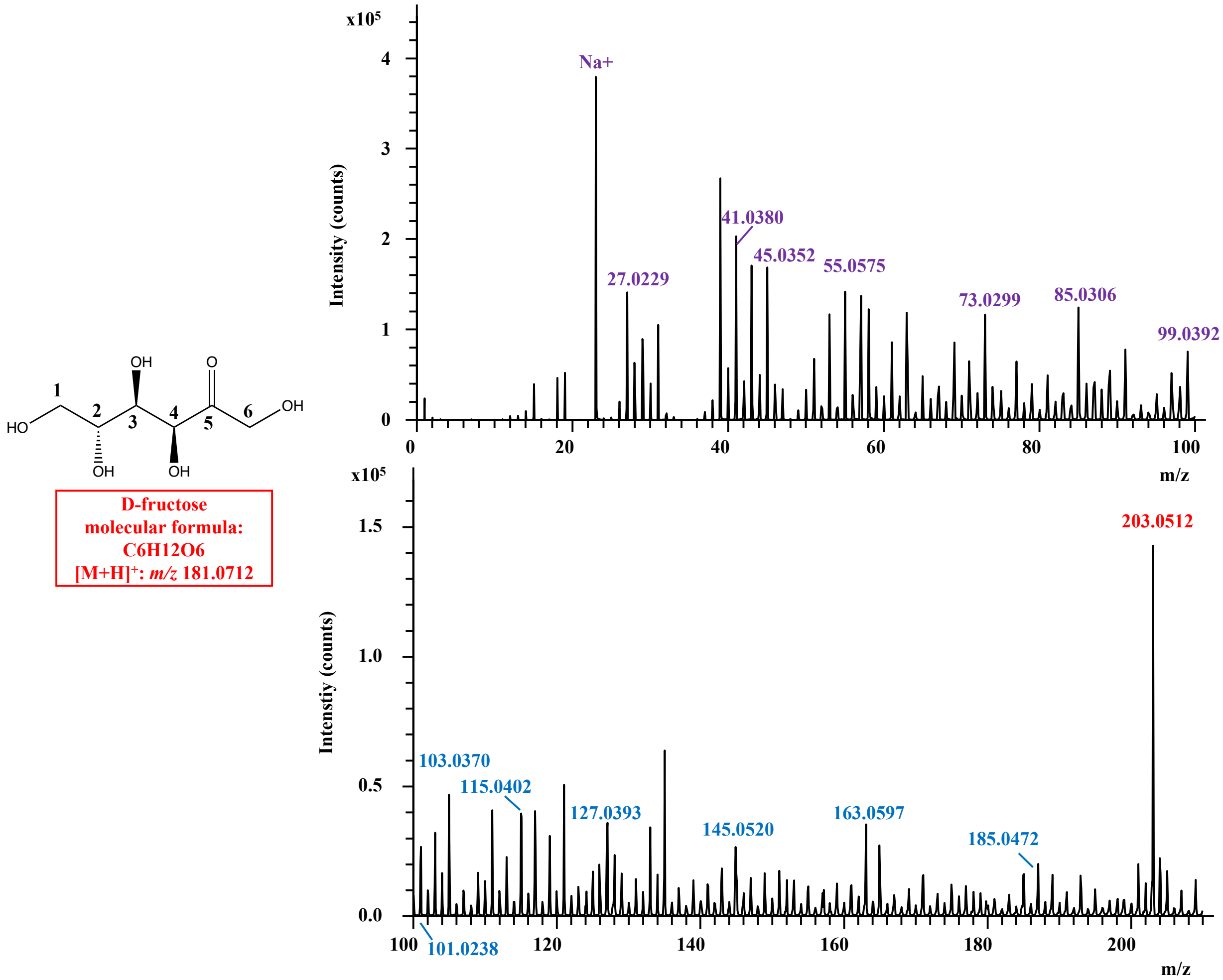

Supplementary Figure S21. The study on TOF-SIMS spectra of D-fructose standard in the positive mode.

Supplementary Table S10. The peak assignments of D-fructose standard in TOF-SIMS spectra in the positive mode.

No.	Observed mass (*m/z*)	Chemical formula	Deviation (ppm)	Species
		required peaks		
1	203.0564	$C6H12O6Na^{+}$	18.8	$[M+Na]^{+}$

		characteristic peaks		
2	185.0427	$C6H10O5Na^+$	3.4	$[M-(H_2O)+Na]^+$
3	163.0617	$C6H11O5^+$	9.6	$[M-(H_2O)+H]^+$
4	145.0520	$C6H9O4^+$	16.7	$[M-2(H_2O)+H]^+$
5	127.0379	$C6H7O3^+$	-8.5	$[M-3(H_2O)+H]^+$
6	115.0362	$C5H7O3^+$	-23.7	-
7	103.0361	$C4H7O3^+$	-27.4	-
8	101.0287	$C4H5O3^+$	53.0	-
		other peaks		
9	104.9898	$C2H3O2Na2^+$	-23.8	-
10	99.0465	$C5H7O2^+$	24.7	-
11	97.0290	$C5H5O2^+$	6.0	-
12	87.0531	$C4H7O2^+$	103.5	-
13	85.0348	$C4H5O2^+$	75.7	-
14	73.0326	$C3H5O2^+$	56.8	-
15	71.0499	$C4H7O^+$	11.2	-
16	57.0343	$C3H5O^+$	13.9	-
17	55.0183	$C3H3O^+$	8.9	-
18	45.0341	$C2H5O^+$	14.4	-
19	41.0399	$C3H5^+$	31.8	-
20	31.0175	$CH3O^+$	-10.6	-
21	27.0233	$C2H3^+$	12.4	-

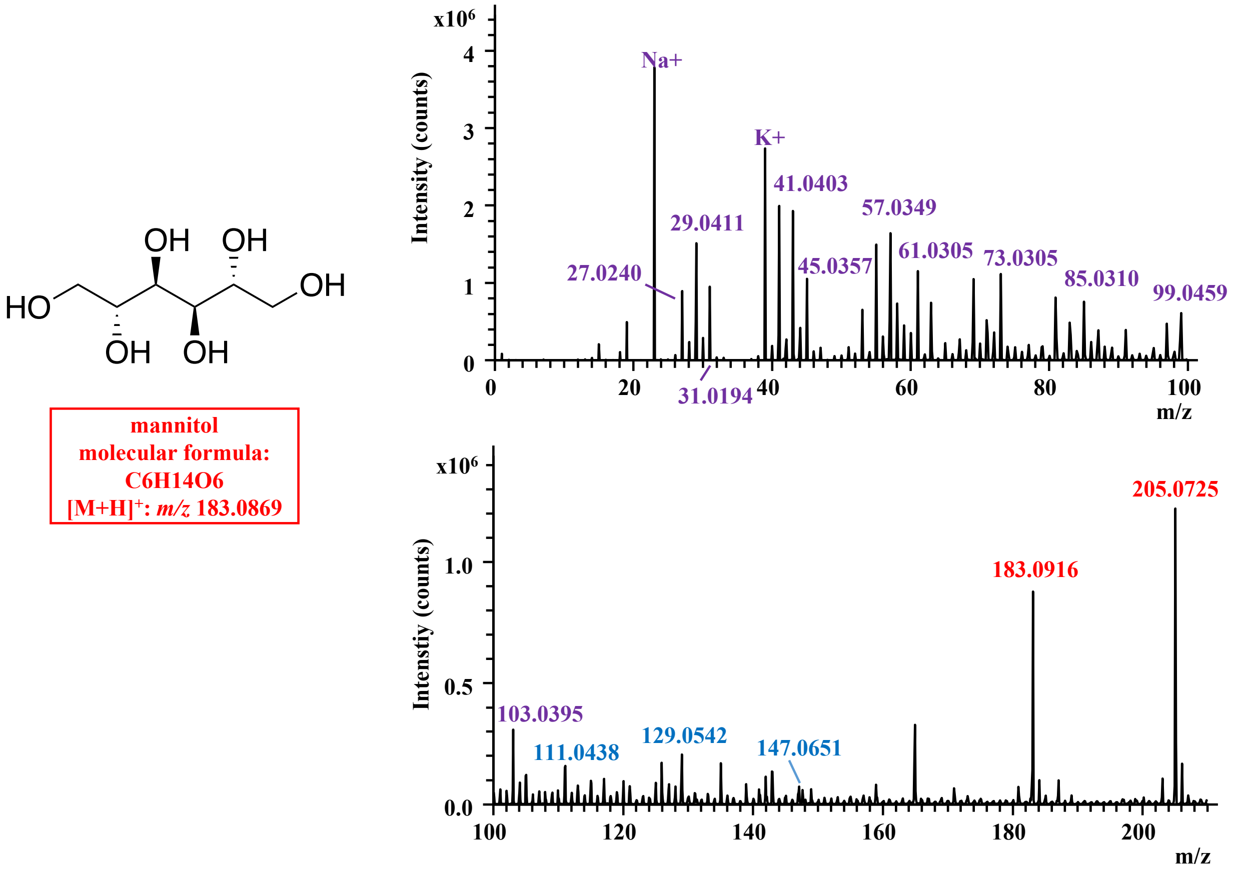

Supplementary Figure S22. The study on TOF-SIMS spectra of mannitol standard in the positive mode.

Supplementary Table S11. The peak assignments of mannitol standard in TOF-SIMS spectra in the positive mode.

No.	Observed mass (*m/z*)	Chemical formula	Deviation (ppm)	Species
		required peaks		
1	183.0916	$C6H15O6^{+}$	28.8	$[M+H]^{+}$
2	205.0725	$C6H14O6Na^{+}$	20.7	$[M+Na]^{+}$
		characteristic peaks		
3	147.0651	$C6H11O4^{+}$	-0.4	$[M+H-2(H2O)]^{+}$
4	129.0542	$C6H9O3^{+}$	-0.7	$[M+H-3(H2O)]^{+}$
5	111.0438	$C6H7O2^{+}$	-2.1	$[M+H-4(H2O)]^{+}$
		other peaks		
6	103.0458	$C4H7O3^{+}$	66.7	-
7	99.0459	$C5H7O2^{+}$	18.1	-
8	85.0310	$C4H5O2^{+}$	30.4	-

9	73.0305	$C3H5O2^{+}$	28.9	-
10	61.0305	$C2H5O2^{+}$	35.0	-
11	57.0349	$C3H5O^{+}$	24.7	-
12	45.0357	$C2H5O^{+}$	48.5	-
13	41.0403	$C3H5^{+}$	43.1	-
14	31.0194	$CH3O^{+}$	49.8	-
15	29.0411	$C2H5^{+}$	86.3	-
16	27.0240	$C2H3^{+}$	39.0	-

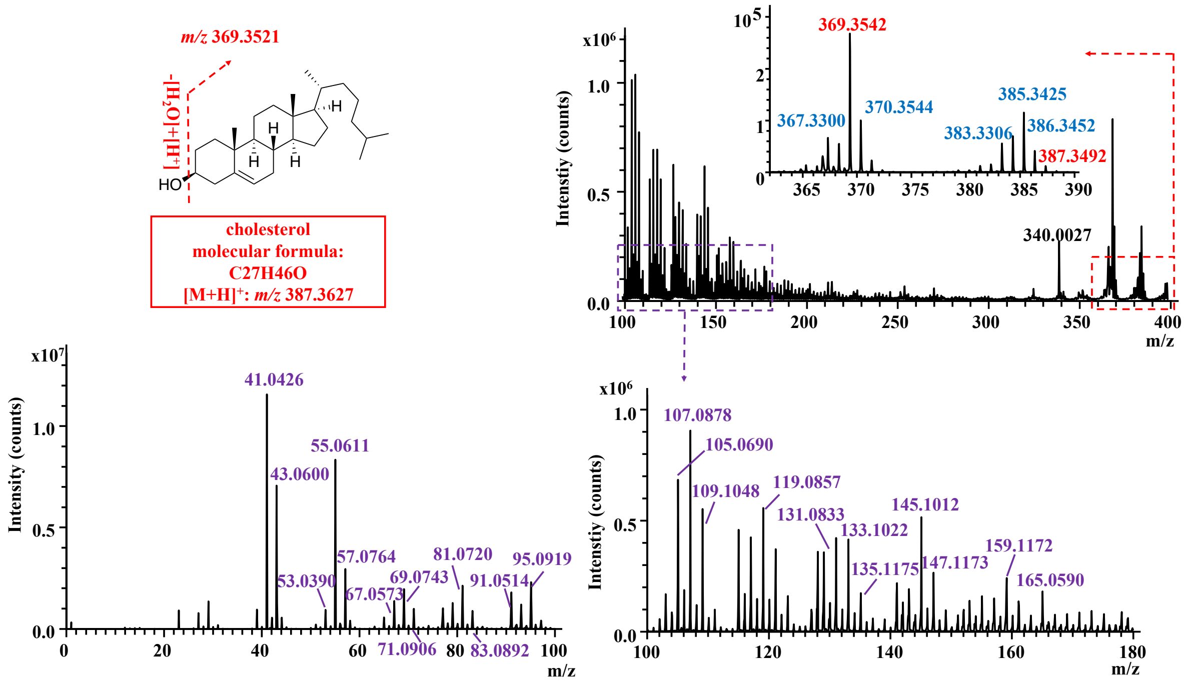

Supplementary Figure S23. The study on TOF-SIMS spectra of cholesterol standard in the positive mode.

Supplementary Table S12. The peak assignments of cholesterol standard in TOF-SIMS spectra in the positive mode.

No.	Observed mass (*m/z*)	Chemical formula	Deviation (ppm)	Species
		required peaks		
1	369.3542	$C27H45^{+}$	7.1	$[M-(H2O)+H]^{+}$
2	387.3492	$C27H47O^{+}$	-33.3	$[M+H]^{+}$
		characteristic peaks		
3	386.3452	$C27H46O^{+}$	-23.6	-

4	385.3425	$C27H45O^{+}$	-10.4	-
5	383.3306	$C27H43O^{+}$	-0.7	-
6	370.3544	$C27H46^{+}$	-13.6	-
7	367.3300	$C27H43^{+}$	-8.0	-
		other peaks		
8	165.0590	$C9H9O3^{+}$	26.8	-
9	159.1172	$C12H15^{+}$	2.1	-
10	147.1173	$C11H15^{+}$	2.1	-
11	145.1012	$C11H13^{+}$	0.2	-
12	135.1175	$C10H15^{+}$	4.9	-
13	133.1022	$C10H13^{+}$	7.7	-
14	131.0833	$C10H11^{+}$	16.8	-
15	119.0857	$C9H11^{+}$	1.7	-
16	109.1048	$C8H13^{+}$	33.5	-
17	107.0878	$C8H11^{+}$	21.3	-
18	105.0690	$C8H9^{+}$	-8.6	-
19	95.0919	$C7H11^{+}$	66.5	-
20	91.0514	$C7H7^{+}$	-30.6	-
21	83.0892	$C6H11^{+}$	43.6	-
22	81.0720	$C6H9^{+}$	26.1	-
23	71.0906	$C5H11^{+}$	71.3	-
24	69.0743	$C5H9^{+}$	64.4	-
25	67.0573	$C5H7^{+}$	46.2	-
26	57.0764	$C4H9^{+}$	115.1	-
27	55.0611	$C4H7^{+}$	125.1	-
28	53.0390	$C4H5^{+}$	8.2	-
29	43.0600	$C3H7^{+}$	135.1	-
30	41.0426	$C3H5^{+}$	97.3	-

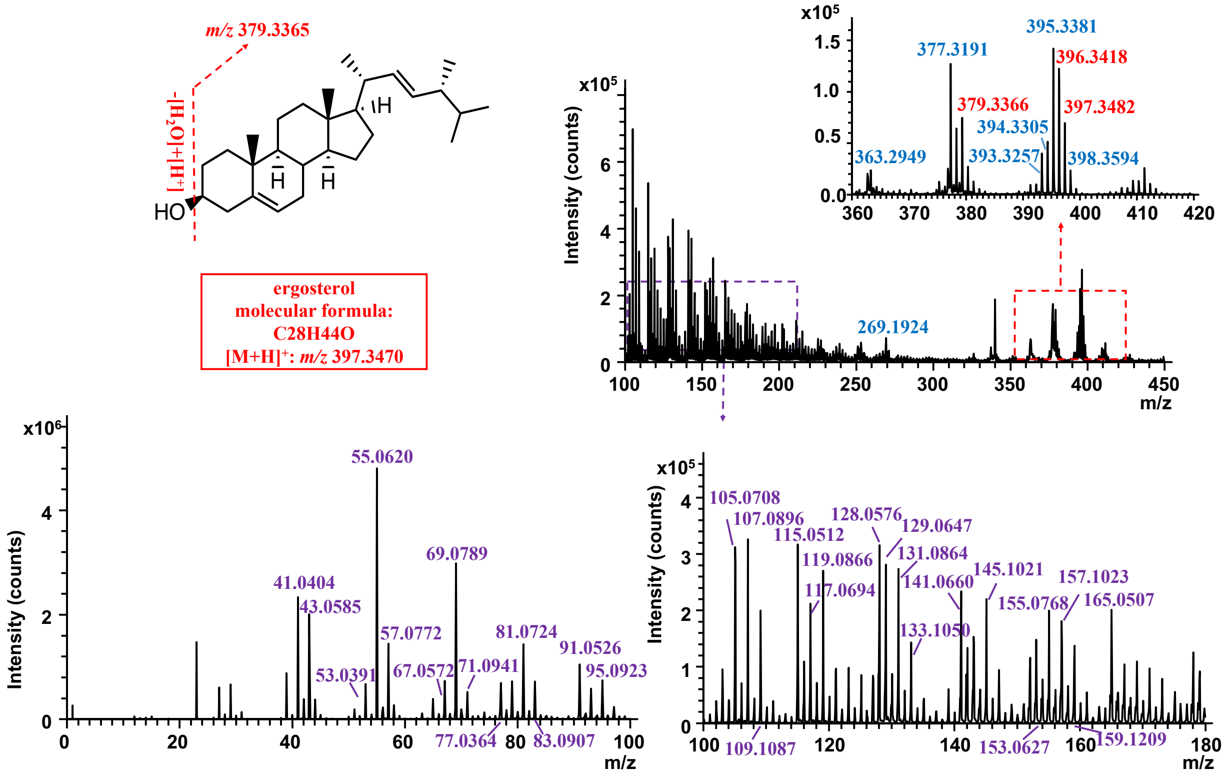

Supplementary Figure S24. The study on TOF-SIMS spectra of ergosterol standard in the positive mode.

Supplementary Table S13. The peak assignments of ergosterol standard in TOF-SIMS spectra in the positive mode.

No.	Observed mass (*m/z*)	Chemical formula	Deviation (ppm)	Species
		required peaks		
1	379.3366	$C28H43^{+}$	1.7	$[M-(H2O)+H]^{+}$
2	397.3482	$C28H45O^{+}$	4.3	$[M+H]^{+}$
3	396.3418	$C28H44O^{+}$	8.0	$[M]^{+\cdot}$
		characteristic peaks		
4	398.3594	$C28H46O^{+}$	12.8	-
5	395.3381	$C28H43O^{+}$	18.3	-
6	394.3305	$C28H42O^{+}$	19.1	-
7	393.3257	$C28H41O^{+}$	26.6	-
8	377.3191	$C28H41^{+}$	-3.2	-
9	363.2949	$C27H39^{+}$	-26.9	-
10	269.1924	$C19H25O^{+}$	8.8	-

11	145.1021	$C11H13^{+}$	6.6	-
12	141.0660	$C11H9^{+}$	-27.6	-
		other peaks		
13	165.0507	$C9H9O3^{+}$	-23.8	-
14	159.1209	$C12H15^{+}$	25.6	-
15	157.1023	$C12H13^{+}$	7.2	-
16	155.0768	$C12H11^{+}$	-56.1	-
17	153.0627	$C12H9^{+}$	-46.9	-
18	145.1037	$C11H13^{+}$	17.4	-
19	141.0660	$C11H9^{+}$	-27.6	-
20	133.1050	$C10H13^{+}$	28.4	-
21	131.0864	$C10H11^{+}$	6.6	-
22	129.0647	$C10H9^{+}$	-40.1	-
23	128.0558	$C10H8^{+}$	-49.0	-
24	119.0881	$C9H11^{+}$	21.8	-
25	117.0694	$C9H9^{+}$	-4.0	-
26	115.0497	$C9H7^{+}$	-38.9	-
27	109.1087	$C8H13^{+}$	68.7	-
28	107.0914	$C8H11^{+}$	54.8	-
29	105.0708	$C8H9^{+}$	8.5	-
30	95.0923	$C7H11^{+}$	70.7	-
31	91.0526	$C7H7^{+}$	-17.9	-
32	83.0907	$C6H11^{+}$	62.8	-
33	81.0724	$C6H9^{+}$	30.6	-
34	77.0364	$C6H5^{+}$	-28.7	-
35	71.0941	$C5H11^{+}$	120.1	-
36	69.0789	$C5H9^{+}$	129.9	-
37	67.0572	$C5H7^{+}$	44.9	-
38	57.0772	$C4H9^{+}$	127.5	-
39	55.0620	$C4H7^{+}$	141.1	-

40	53.0391	$C4H5^+$	9.6	-
41	43.0585	$C3H7^+$	100.4	-
51	41.0404	$C3H5^+$	44.8	-

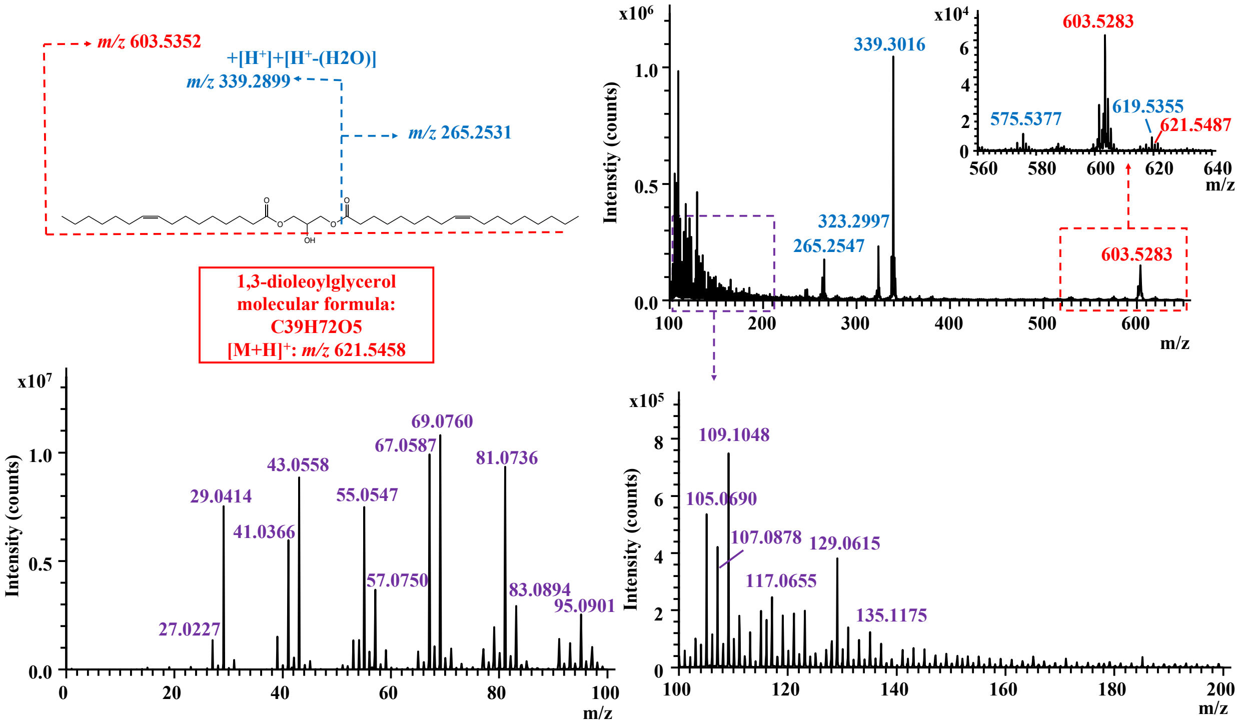

Supplementary Figure S25. The study on TOF-SIMS spectra of 1,3-dioleoylglycerol standard in the positive mode.

Supplementary Table S14. The peak assignments of 1,3-dioleoylglycerol standard in TOF-SIMS spectra in the positive mode.

No.	Observed mass (*m/z*)	Chemical formula	Deviation (ppm)	Species
		required peaks		
1	603.5283	$C39H71O4^+$	-10.5	$[M-(H2O)+H]^+$
2	621.5487	$C39H73O5^+$	5.6	$[M+H]^+$
		characteristic peaks		
3	619.5355	$C39H71O5^+$	9.5	-
4	575.5377	$C34H71O6^+$	22.9	-
5	339.3016	$C21H39O3^+$	36.0	$[M(MAG)-(H2O)+H]^+$
6	323.2997	$C21H39O2^+$	16.4	-
7	265.2547	$C18H33O^+$	8.0	$[M(oleic acid)-(H2O)+H]^+$

		other peaks		
8	135.1175	$C10H15^{+}$	4.9	-
9	129.0615	$C10H9^{+}$	-64.9	-
10	117.0655	$C9H9^{+}$	-37.7	-
11	109.1048	$C8H13^{+}$	33.5	-
12	107.0878	$C8H11^{+}$	21.3	-
13	105.0690	$C8H9^{+}$	-8.6	-
14	95.0901	$C7H11^{+}$	48.6	-
15	83.0894	$C6H11^{+}$	46.1	-
16	81.0736	$C6H9^{+}$	45.8	-
17	69.0760	$C5H9^{+}$	89.4	-
18	67.0587	$C5H7^{+}$	67.4	-
19	57.0750	$C4H9^{+}$	89.5	-
20	55.0547	$C4H7^{+}$	9.3	-
21	43.0558	$C3H7^{+}$	37.2	-
22	41.0366	$C3H5^{+}$	37.2	-
23	29.0414	$C2H5^{+}$	97.4	-
24	27.0227	$C2H3^{+}$	-7.8	-

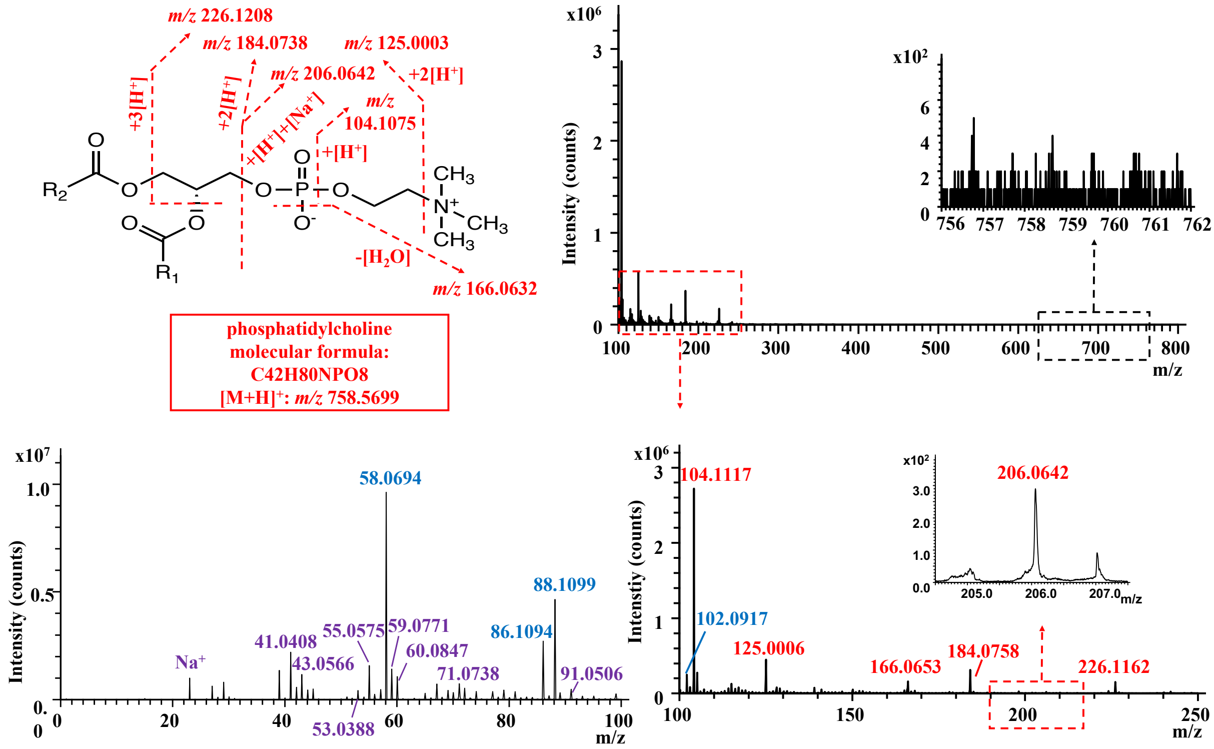

Supplementary Figure S26. The study on TOF-SIMS spectra of phosphatidylcholine standard in the positive mode.

Supplementary Table S15. The peak assignments of phosphatidylcholine standard in TOF-SIMS spectra in the positive mode.

No.	Observed mass (*m/z*)	Chemical formula	Deviation (ppm)	Species
		required peaks		
1	226.1223	$C8H21NPO4^{+}$	8.9	-
2	206.0642	$C5H14NPO4Na^{+}$	43.5	
3	184.0758	$C5H15NPO4^{+}$	13.7	-
4	166.0653	$C5H13NPO3^{+}$	15.2	-
5	125.0006	$C2H6PO4^{+}$	6.0	-
6	104.1117	$C5H14NO^{+}$	44.9	-
		characteristic peaks		
7	102.0912	$C5H12NO^{+}$	-1.1	-
8	88.1099	$C5H14N^{+}$	113.0	-
9	86.1094	$C5H12N^{+}$	150.6	-
10	58.0694	$C3H8N^{+}$	73.2	-

		other peaks		
11	91.0506	C7H7$_+$	-40.1	-
12	71.0738	C4H9N$_+$	12.5	-
13	60.0847	C3H10N$_+$	66.0	-
14	59.0771	C3H9N$_+$	69.6	-
15	55.0575	C4H7$^+$	60.1	-
16	53.0388	C4H5$^+$	5.0	-
17	43.0566	C3H7$^+$	54.1	-
18	41.0408	C3H5$^+$	53.7	-

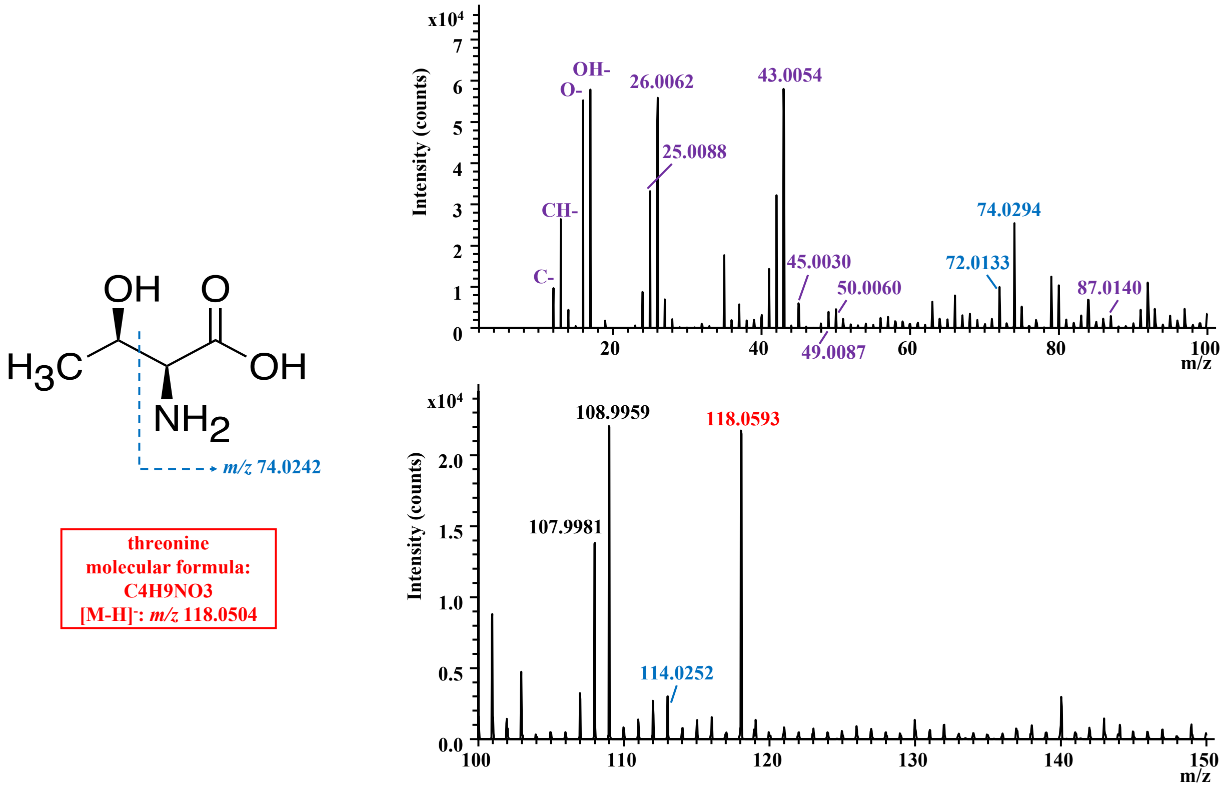

Supplementary Figure S27. The study on TOF-SIMS spectra of threonine standard in the negative mode.

Supplementary Table S16. The peak assignments of threonine standard in TOF-SIMS spectra in the negative mode.

No.	Observed mass (*m/z*)	Chemical formula	Deviation (ppm)	Species
		required peaks		
1	118.0593	C4H8NO3$^-$	70.7	[M-H]$^-$
		characteristic peaks		

2	114.0252	C4H4NO3$^-$	48.9	-
3	74.0294	C2H4NO2$^-$	62.3	[M-CH3CHOH]$^-$
4	72.0133	C2H2NO2$^-$	58.4	-
		other peaks		
5	87.0140	C3H3O3$^-$	60.6	-
6	50.0060	C3N$^-$	47.6	-
7	49.0087	C4H$^-$	6.1	-
8	45.0030	CHO2$^-$	47.5	-
9	43.0054	CHNO$^-$	-22.0	-
10	25.0088	C2H$^-$	18.9	-
11	26.0062	CN$^-$	97.5	-

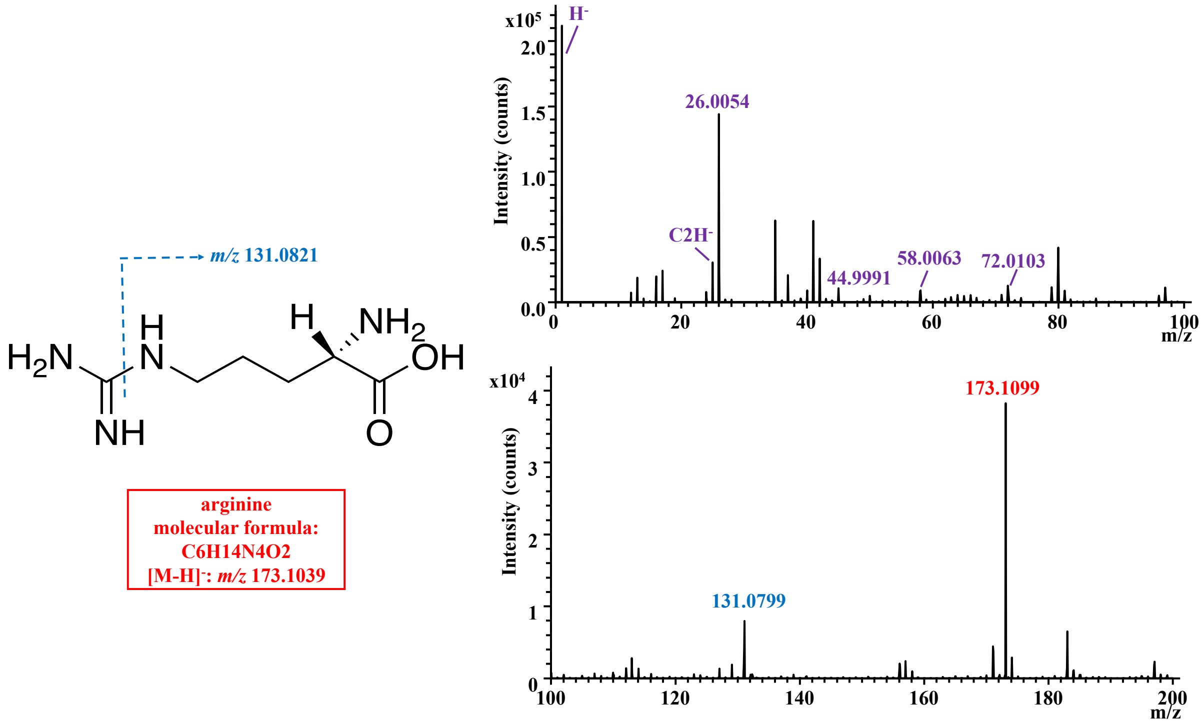

Supplementary Figure S28. The study on TOF-SIMS spectra of arginine standard in the negative mode.

Supplementary Table S17. The peak assignments of arginine standard in TOF-SIMS spectra in the negative mode.

No.	Observed mass (*m/z*)	Chemical formula	Deviation (ppm)	Species
		required peaks		
1	173.1099	C6H13N4O2$^-$	31.9	[M-H]$^-$

		characteristic peaks		
2	131.0799	$C5H11N2O2^-$	-20.6	-
		other peaks		
3	72.0103	$C2H2NO2^-$	16.8	-
4	58.0063	$C2H2O2^-$	5.4	-
5	44.9991	$CHO2^-$	19.7	-
6	26.0054	CN^-	69.8	-

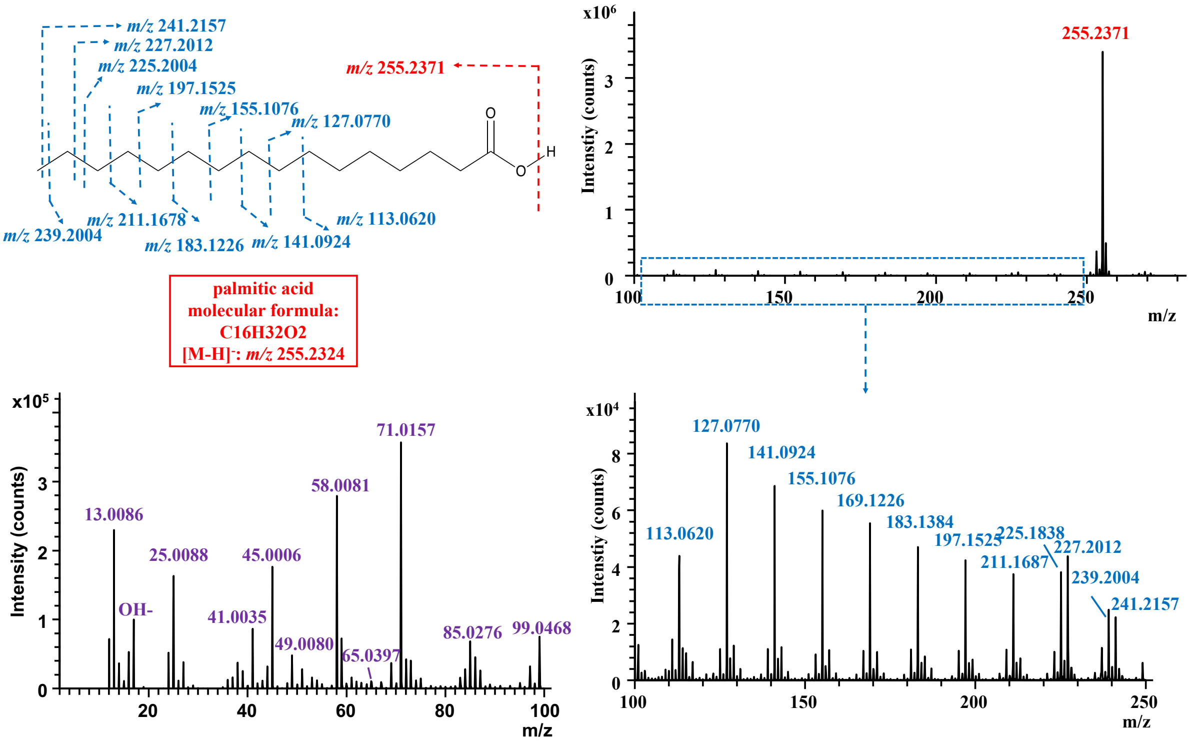

Supplementary Figure S29. The study on TOF-SIMS spectra of palmitic acid standard in the negative mode.

Supplementary Table S18. The peak assignments of palmitic acid standard in TOF-SIMS spectra in the negative mode.

No.	Observed mass (*m/z*)	Chemical formula	Deviation (ppm)	Species
		required peaks		
1	255.2371	$C16H31O2^-$	16.3	$[M-H]^-$
		characteristic peaks		
2	253.2176	$C16H29O2^-$	1.0	$[M-3H]^-$
3	241.2157	$C15H29O2^-$	-6.5	$[M-CH3]^-$

4	239.2004	$C15H27O2^-$	-5.2	$[M-(CH4)-H]^-$
5	227.2012	$C14H27O2^-$	-2.2	$[M-(C2H5)]^-$
6	225.1838	$C14H25O2^-$	-9.8	$[M-(C2H6)-H]^-$
7	211.1678	$C13H23O2^-$	-1.7	$[M-(C3H8)-H]^-$
8	197.1525	$C12H21O2^-$	-10.9	$[M-(C4H10)-H]^-$
9	183.1384	$C11H19O2^-$	-3.4	$[M-(C5H12)-H]^-$
10	169.1226	$C10H17O2^-$	-4.8	$[M-(C6H14)-H]^-$
11	155.1076	$C9H15O2^-$	-0.9	$[M-(C7H16)-H]^-$
12	141.0924	$C8H13O2^-$	2.4	$[M-(C8H18)-H]^-$
13	127.0770	$C7H11O2^-$	4.5	$[M-(C9H20)-H]^-$
14	113.0620	$C6H9O2^-$	10.8	$[M-(C10H22)-H]^-$
		other peaks		
15	99.0468	$C5H7O2^-$	16.6	-
16	85.0276	$C4H5O2^-$	-22.7	-
17	71.0157	$C3H3O2^-$	26.1	-
18	65.0397	$C5H5^-$	0.5	-
19	58.0081	$C2H2O2^-$	35.5	-
20	49.0080	$C4H^-$	-6.8	-
21	45.0006	$CHO2^-$	53.2	-
22	41.0035	$C2HO^-$	5.5	-
23	25.0088	$C2H^-$	18.9	-

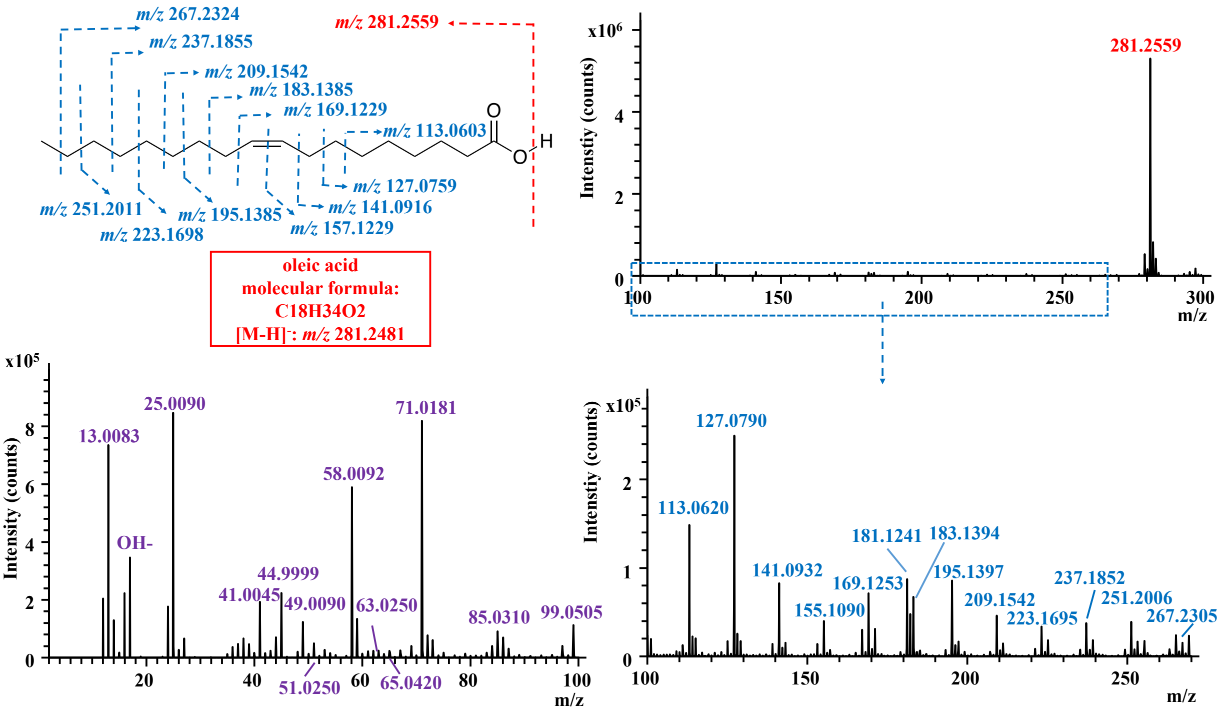

Supplementary Figure S30. The study on TOF-SIMS spectra of oleic acid standard in the negative mode.

Supplementary Table S19. The peak assignments of oleic acid standard in TOF-SIMS spectra in the negative mode.

No.	Observed mass (*m/z*)	Chemical formula	Deviation (ppm)	Species
		required peaks		
1	281.2559	$C18H33O2^-$	25.8	$[M-H]^-$
		characteristic peaks		
2	279.2400	$C18H31O2^-$	25.2	$[M-3H]^-$
3	267.2305	$C17H31O2^-$	-9.3	$[M-(CH3)]^-$
4	251.2006	$C16H27O2^-$	-4.2	$[M-(C2H6)-H]^-$
5	237.1852	$C15H25O2^-$	-3.4	$[M-(C3H8)-H]^-$
6	223.1695	$C14H23O2^-$	-4.0	$[M-(C4H10)-H]^-$
7	209.1542	$C13H21O2^-$	-2.3	$[M-(C5H12)-H]^-$
8	195.1397	$C12H19O2^-$	3.5	$[M-(C6H14)-H]^-$
9	183.1394	$C11H19O2^-$	2.0	$[M-(C7H15)]^-$
10	181.1241	$C11H17O2^-$	3.7	$[M-(C7H16)-H]^-$
11	169.1253	$C10H17O2^-$	11.3	$[M-(C8H17)]^-$
12	155.1090	$C9H15O2^-$	7.8	$[M-(C9H20)-H]^-$

13	141.0932	$C8H13O2^-$	7.4	$[M-(C10H22)-H]^-$
14	127.0790	$C7H11O2^-$	20.3	$[M-(C11H24)-H]^-$
15	113.0620	$C6H9O2^-$	11.0	$[M-(C12H26)-H]^-$
		other peaks		
16	99.0505	$C5H7O2^-$	54.4	-
17	85.0310	$C4H5O2^-$	17.3	-
18	71.0181	$C3H3O2^-$	60.6	-
19	65.0420	$C5H5^-$	35.5	-
20	63.0250	$C5H3^-$	15.8	-
21	58.0092	$C2H2O2^-$	55.4	-
22	51.0250	$C4H3^-$	18.3	-
23	49.0090	$C4H^-$	13.4	-
24	44.9999	$CHO2^-$	37.6	-
25	41.0045	$C2HO^-$	28.7	-
26	25.0090	$C2H^-$	24.3	-

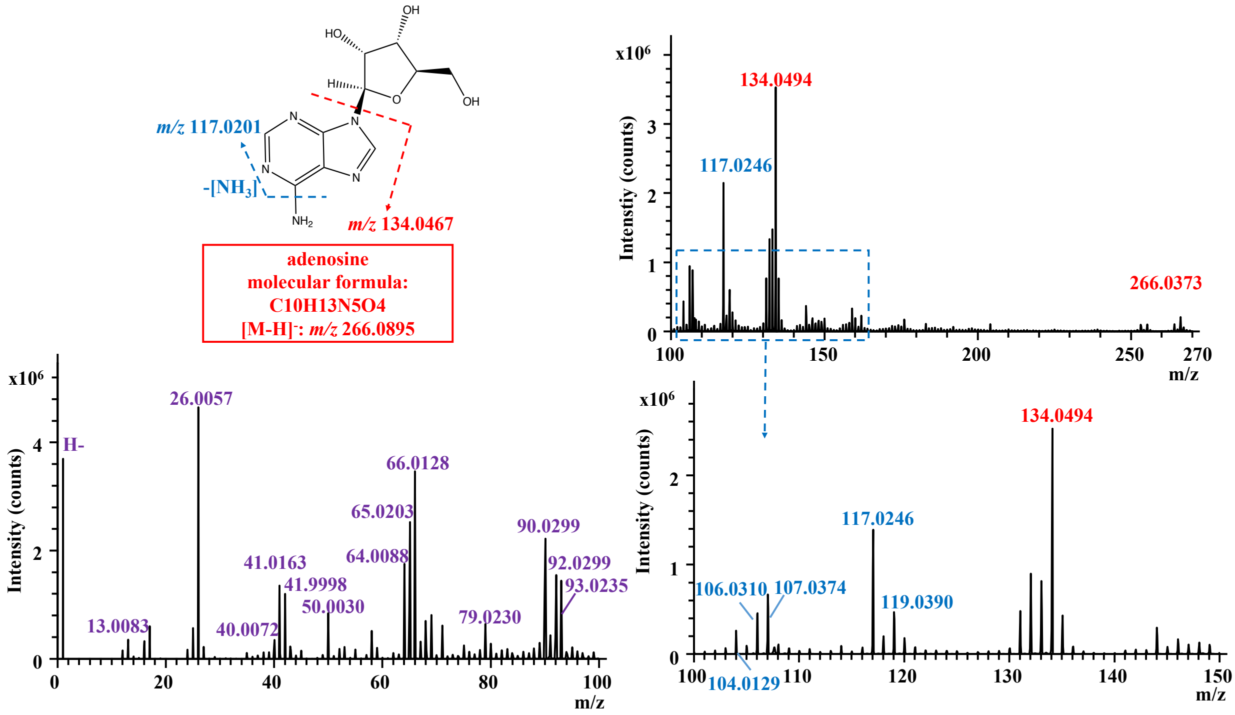

Supplementary Figure S31. The study on TOF-SIMS spectra of adenosine standard in the negative mode.

Supplementary Table S20. The peak assignments of adenosine standard in TOF-SIMS spectra in the negative mode.

No.	Observed mass (*m/z*)	Chemical formula	Deviation (ppm)	Species
		required peaks		
1	266.1170	$C10H12N5O4^{-}$	28.9	$[M-H]^{-}$
		characteristic peaks		
2	134.0478	$C5H4N5^{-}$	4.6	$[M(adenine)-H]^{-}$
3	119.0390	$C5H3N4^{-}$	22.6	-
4	117.0190	$C5HN4^{-}$	-14.2	$[M(adenine)-(NH3)-H]^{-}$
5	107.0388	$C4H3N4^{-}$	23.1	-
6	106.0310	$C4H2N4^{-}$	24.0	-
7	104.0142	$C4N4^{-}$	13.4	-
		other peaks		
8	93.0235	$C2H5O4^{-}$	44.6	-
9	92.0299	$C5H4N2^{-}$	-88.5	-
10	90.0299	$C5H2N2^{-}$	-56.5	-
11	79.0230	$C3HN3^{-}$	68.1	-
12	66.0128	$C2N3^{-}$	46.4	-
13	65.0203	$C3HN2^{-}$	89.1	-
14	64.0088	$C3N2^{-}$	32.5	-
15	50.0029	$C3N^{-}$	-13.6	-
16	41.9998	CNO^{-}	29.8	-
17	41.0168	$CHN2^{-}$	54.5	-
18	40.0063	$CN2^{-}$	-10.2	-
19	26.0049	CN^{-}	50.6	-

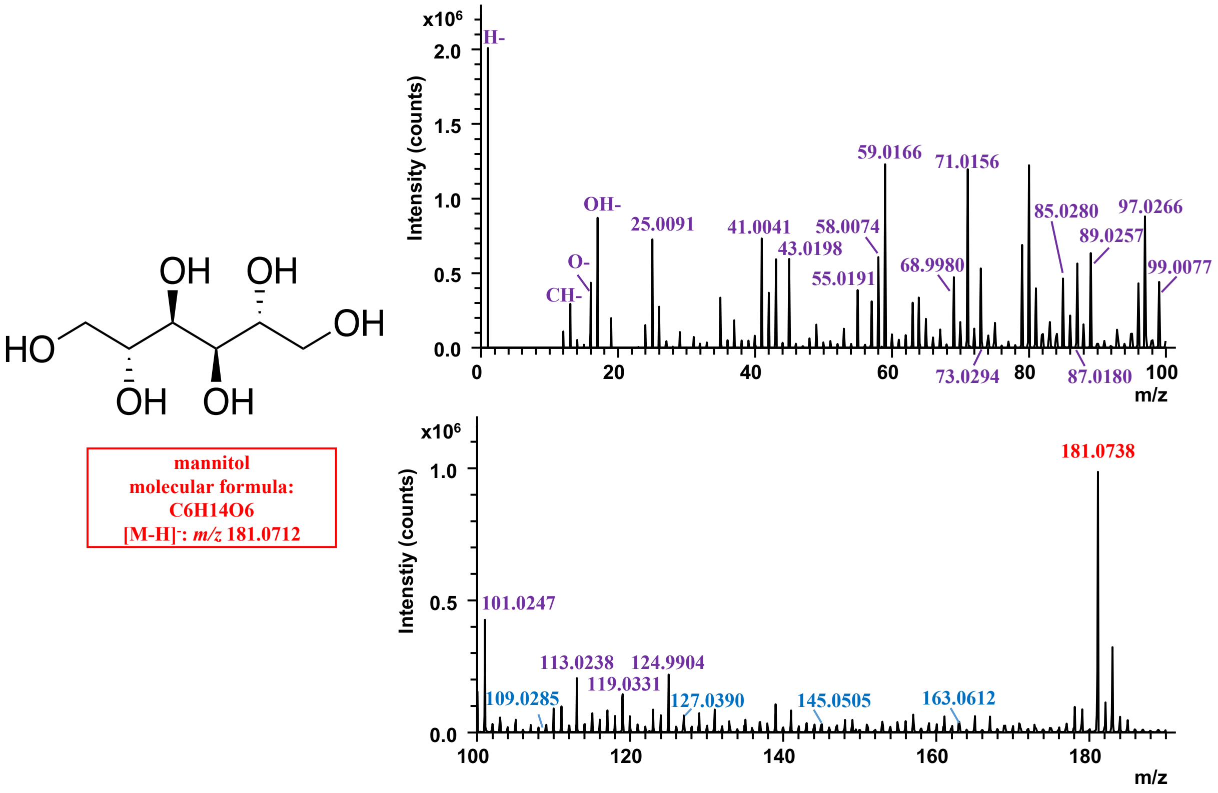

Supplementary Figure S32. The study on TOF-SIMS spectra of mannitol standard in the negative mode.

Supplementary Table S21. The peak assignments of mannitol standard in TOF-SIMS spectra in the negative mode.

No.	Observed mass (*m/z*)	Chemical formula	Deviation (ppm)	Species
		required peaks		
1	181.0738	C6H13O6$^-$	11.5	[M-H]$^-$
		characteristic peaks		
2	163.0612	C6H11O5$^-$	-0.2	[M-(H2O)-H]$^-$
3	145.0505	C6H9O4$^-$	-1.3	[M-2(H2O)-H]$^-$
4	127.0390	C6H7O3$^-$	-8.6	[M-3(H2O)-H]$^-$
5	109.0285	C6H5O2$^-$	-9.0	[M-4(H2O)-H]$^-$
		other peaks		
6	124.9904	C5HO4$^-$	19.3	-
7	119.0331	C4H7O4$^-$	-16.1	-
8	113.0238	C5H5O3$^-$	-5.5	-
9	101.0247	C4H5O3$^-$	2.8	-

10	99.0077	$C4H3O3^-$	-11.1	-
11	97.0266	$C5H5O2^-$	-29.7	-
12	89.0257	$C3H5O3^-$	14.8	-
13	87.0108	$C3H3O3^-$	23.8	-
14	85.0280	$C4H5O2^-$	-17.5	-
15	73.0294	$C3H5O2^-$	-1.0	-
16	71.0156	$C3H3O2^-$	24.1	-
17	68.9980	$C3HO2^-$	-3.0	-
18	59.0166	$C2H3O2^-$	46.2	-
19	58.0074	$C2H2O2^-$	23.5	-
20	55.0191	$C3H3O^-$	3.4	-
21	43.0198	$C2H3O^-$	20.6	-
22	41.0041	$C2HO^-$	19.9	-
23	25.0091	$C2H^-$	30.3	-

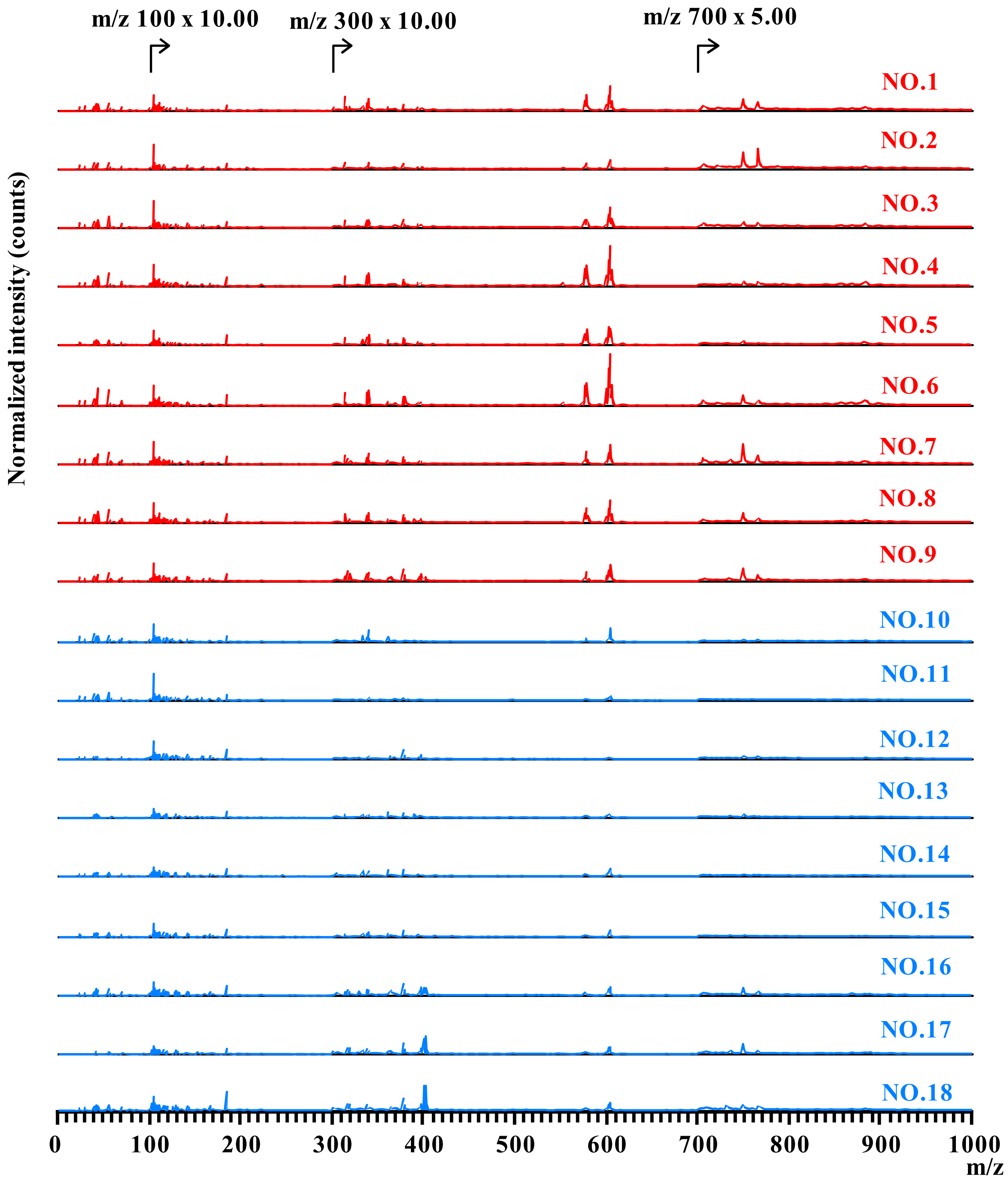

Supplementary Figure S33. The TOF-SIMS fingerprints of *C. sinensis* in positive mode. The representative spectra of CCS (No. 1-9 in red) and NCS (No. 10-18 in blue) samples were normalized by primary ion dose (PID).

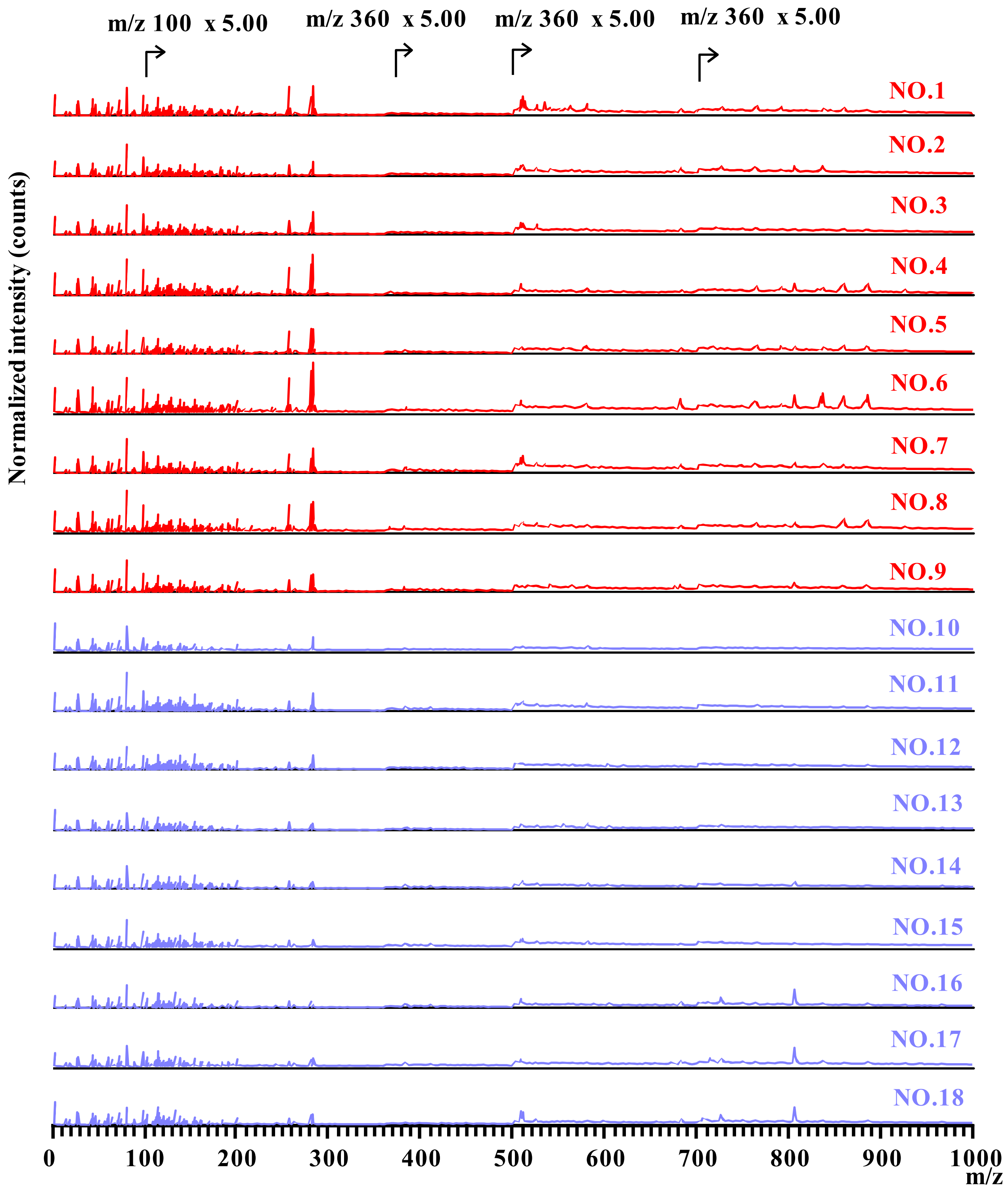

Supplementary Figure S34. The TOF-SIMS fingerprints of *C. sinensis* in negative mode. The representative spectra of CCS (No. 1-9 in red) and NCS (No. 10-18 in blue) samples were normalized by primary ion dose (PID).

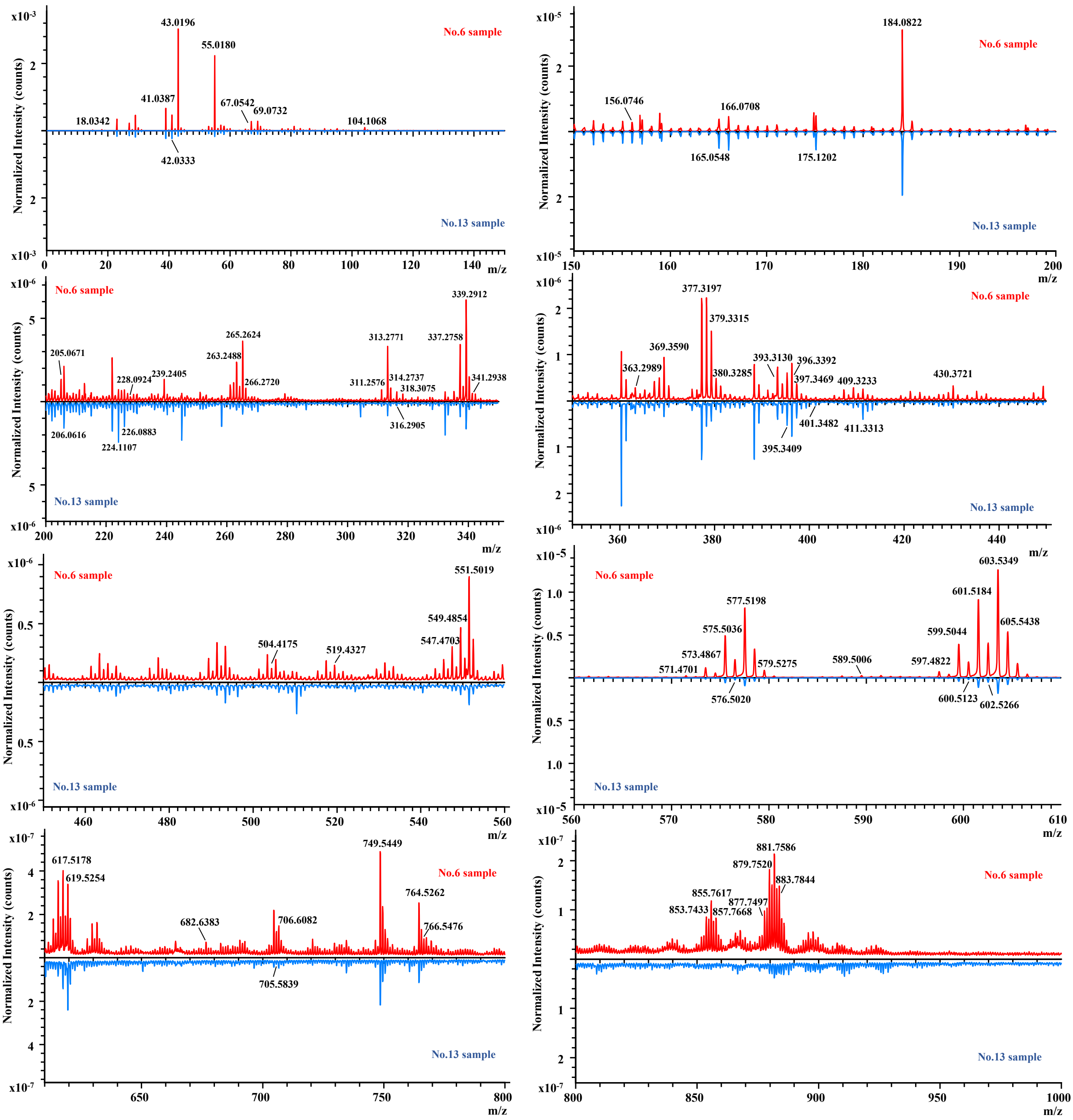

Supplementary Figure S35. The magnified TOF-SIMS spectra of C. sinensis in positive mode. The representative spectra of CCS (No. 6 in red) and NCS (No. 13 in blue) samples were normalized by primary ion dose (PID).

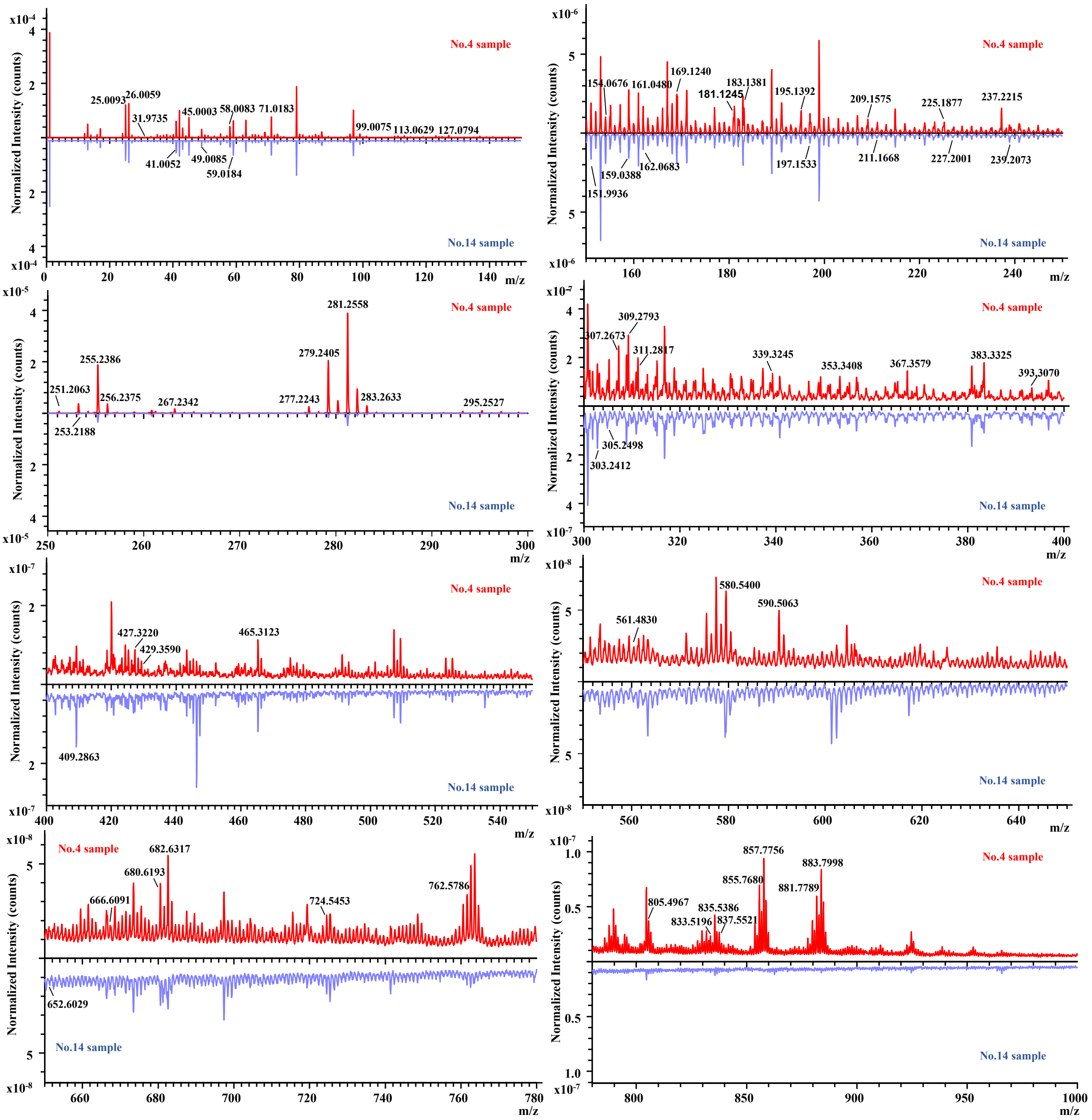

Supplementary Figure S36. The magnified TOF-SIMS spectra of *C. sinensis* in negative mode. The representative spectra of CCS (No. 4 in red) and NCS (No. 14 in blue) samples were normalized by primary ion dose (PID).

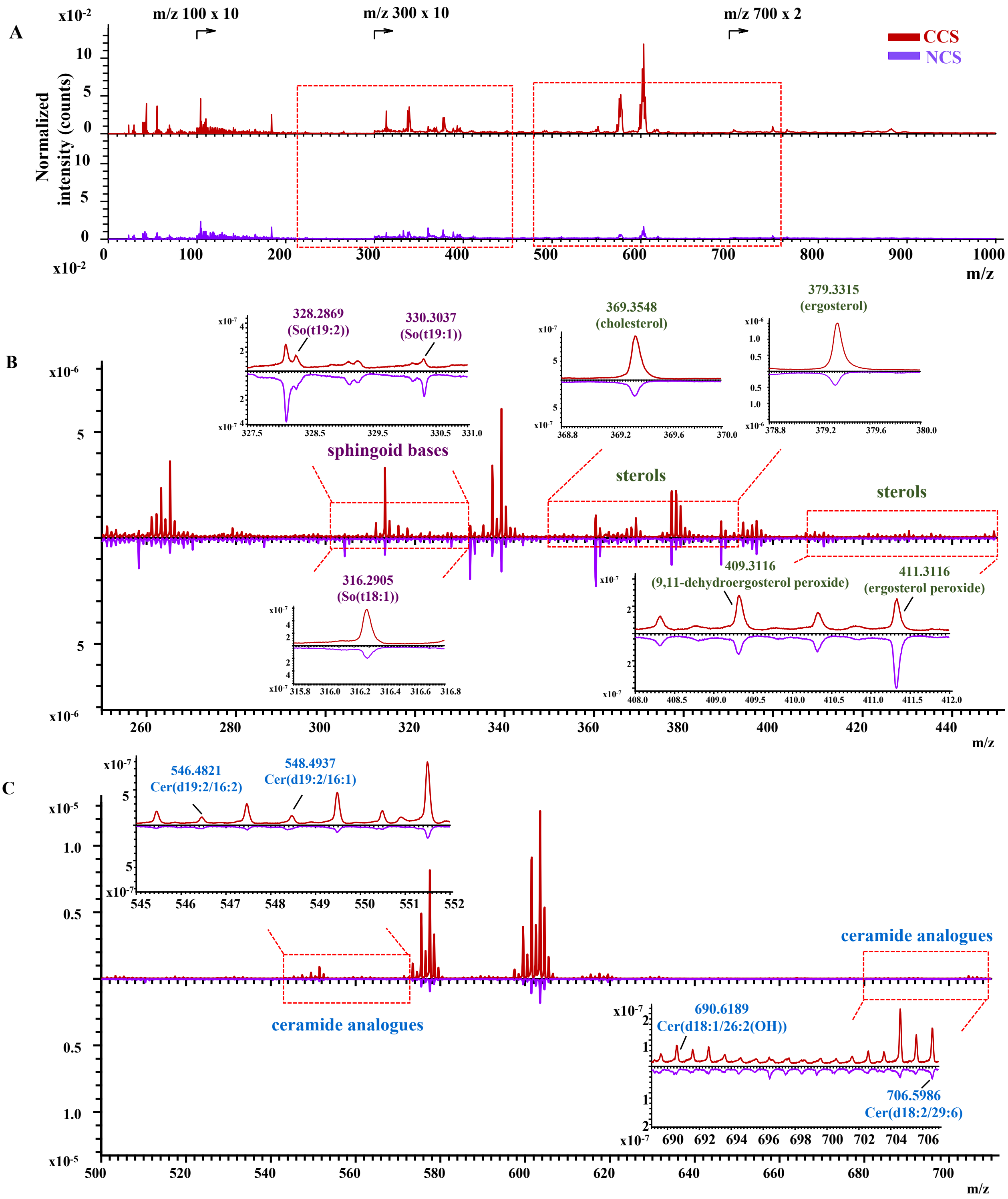

Supplementary Figure S37. The enlarged TOF-SIMS spectra of cultured and natural *C. sinensis* in positive mode. (A) The representative spectra of CCS (No. 6 in red) and NCS (No. 13 in purple) samples. (B) Identification of representative sphingoid bases and sterols in the magnified region with m/z value ranging from 250 to 450. (C) Characterization of representative ceramide analogues in the magnified region with m/z value from 500 to 710. The TOF-SIMS spectra were normalized by primary ion dose (PID).

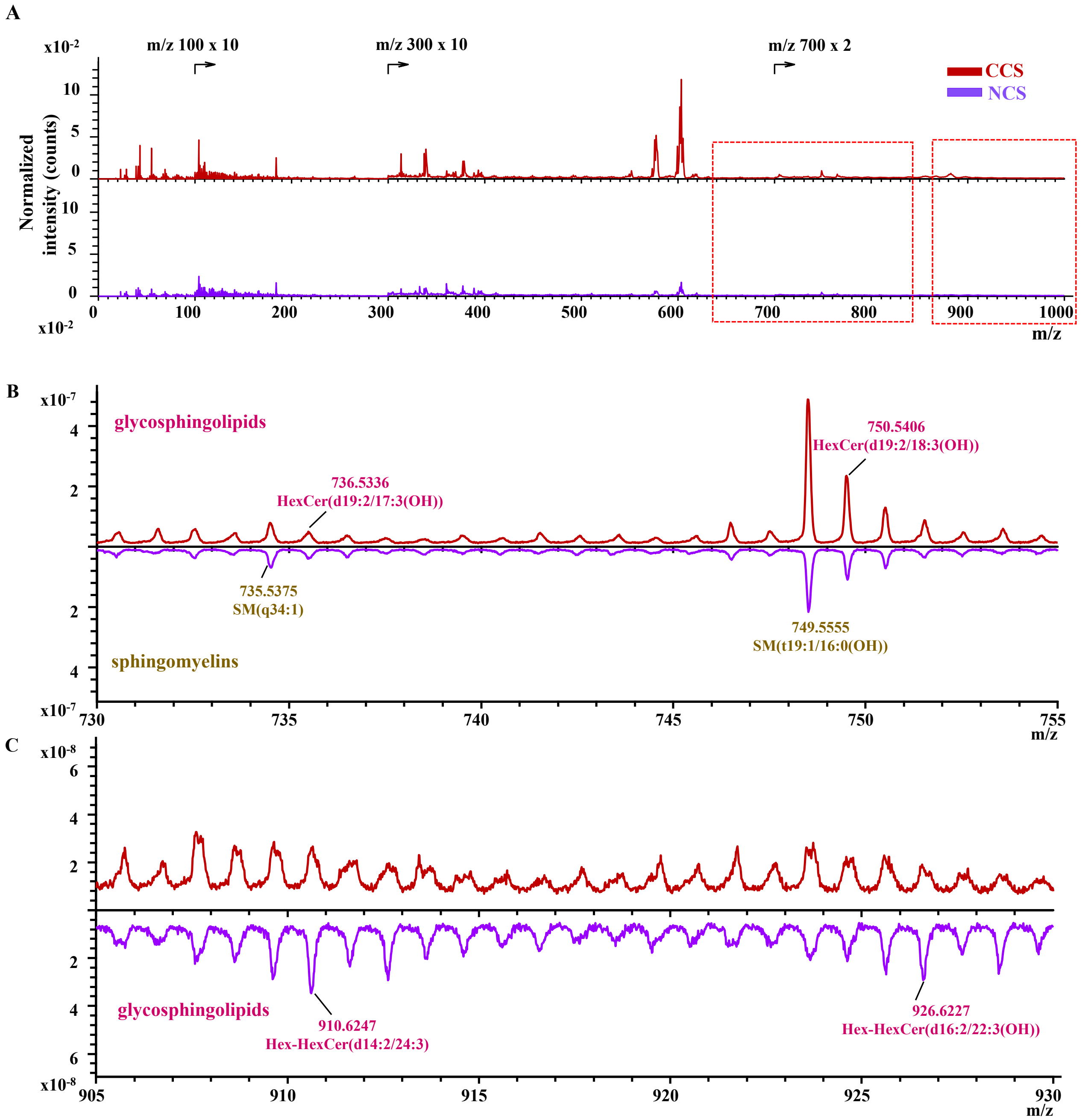

Supplementary Figure S38. The enlarged TOF-SIMS spectra of CCS and NCS samples in positive mode. (A) The representative spectra of CCS (No. 6 in red) and NCS (No. 13 in purple) samples. (B) Identification of representative glycosphingolipids and sphingomyelins in the magnified region with m/z value ranging from 730 to 755 (C) Characterization of representative glycosphingolipids in the magnified region with m/z value from 905 to 930. The TOF-SIMS spectra were normalized by primary ion dose (PID).

Supplementary Table S22. The result of similarity calculation of TOF-SIMS data.

sample	Cosine coefficient		Tanimoto coefficient	
	positive spectra	negative spectra	positive spectra	negative spectra
No.1	0.9768	0.9657	0.9432	0.8857
No.2	0.9515	0.9670	0.9011	0.9156
No.3	0.9593	0.9910	0.8648	0.9818
No.4	0.9844	0.9479	0.9487	0.7518
No.5	0.9783	0.9544	0.9545	0.8527
No.6	0.9674	0.9257	0.9208	0.6013
No.7	0.9857	0.9934	0.9420	0.9842
No.8	0.9907	0.9649	0.9648	0.8252
No.9	0.9918	0.9891	0.9836	0.9778
No.10	0.9882	0.9759	0.9719	0.7484
No.11	0.9454	0.9522	0.8607	0.9079
No.12	0.9821	0.9400	0.9608	0.8865
No.13	0.9644	0.6532	0.8819	0.8100
No.14	0.9419	0.9437	0.8554	0.8638
No.15	0.9870	0.9093	0.9650	0.8150
No.16	0.9691	0.8767	0.9375	0.7560
No.17	0.9273	0.9212	0.8127	0.8232
No.18	0.9093	0.9238	0.8140	0.8304

The correlation coefficient is in the range of 0 to 1. The best, the better or the worst similarity with a corresponding correlation coefficient is above 0.9, between 0.8 and 0.9, or below 0.8, respectively. It is considered as identical if the correlation coefficient equals 1.

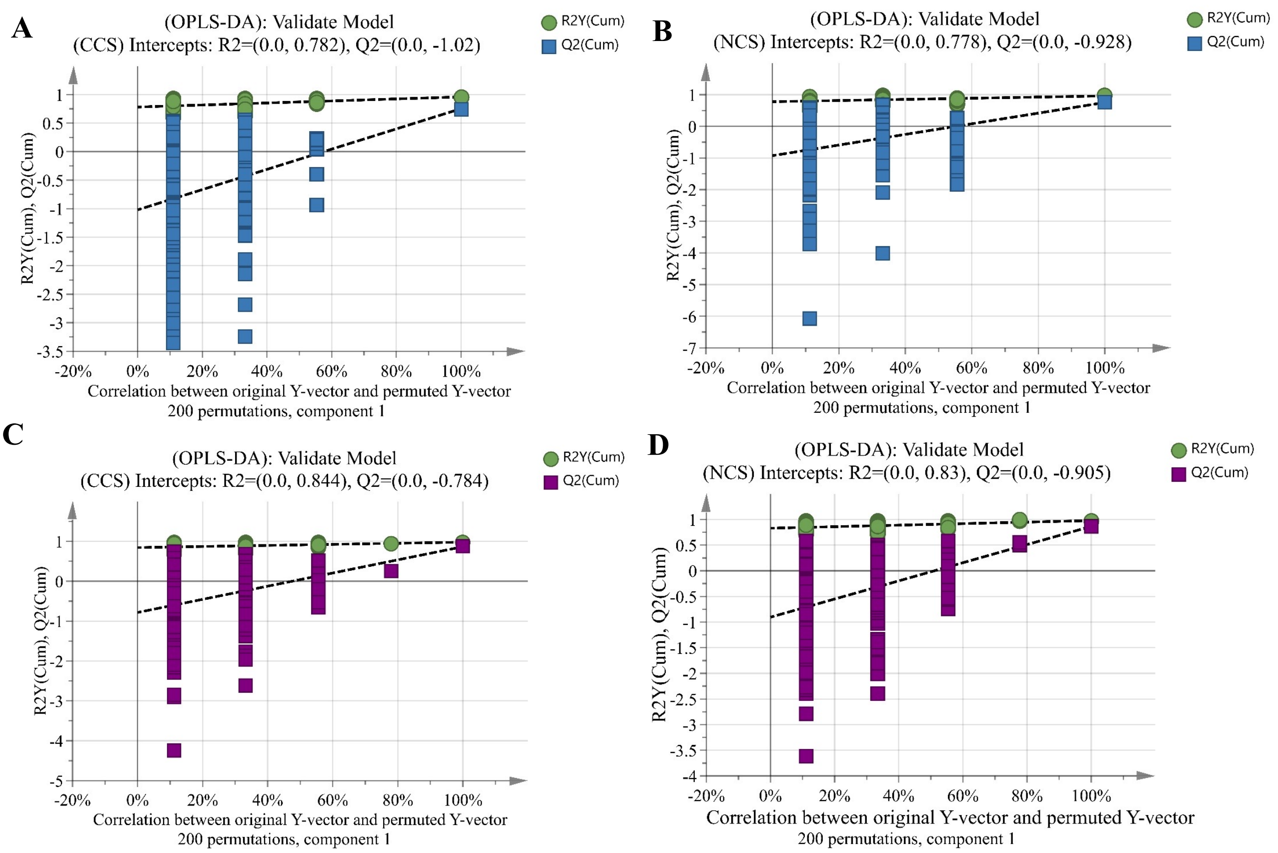

Supplementary Figure S39. The 200 times of permutation test of OPLS-DA mode. The TOF-SIMS spectra dataset in the positive mode (A, B) and negative mode (C, D).

Supplementary Table S23. The differentially expressed components that were screened out by S-plot in positive mode.

polarity	*m/z*	Ion formula	assignment	*p*-value [a]
	104.1100	C5H14NO+	PC fragment	0.0248 *
	603.5370	C39H71O4+	DAG (36:2)	0.0027 **
	577.5200	C37H69O4+	DAG (34:1)	0.0011**
positive mode	110.0780	C5H8N3+	arginine and histidine fragment	0.0135 *
	601.5220	C39H69O4+	DAG (36:3)	0.0039 **
	118.0780	unknown	unknown	/

[a] The p-value was calculated using Student's t-test based on TOF-SIMS data. (n=9). * $p < 0.05$, ** $p < 0.01$, *** $p < 0.001$

Supplementary Table S24. The differentially expressed components that were screened out by S-plot in negative mode

polarity	*m/z*	Ion formula	assignment	p-value [a]
negative mode	281.2620	C18H33O2-	oleic acid (18:1)	0.0015 **
	255.2400	C16H31O2-	palmitic acid (C16:0)	0.0006 ***
	279.2460	C18H31O-	linoleic acid (C18:2)	0.0025 **
	282.2640	C18H34O2-	oleic acid (18:1)	0.0021 **
	132.0480	C4H6NO4-	aspartic acid	0.0030 **

[a] The p-value was calculated using Student's t-test based on TOF-SIMS data. (n=9). * $p < 0.05$, ** $p < 0.01$, *** $p < 0.001$

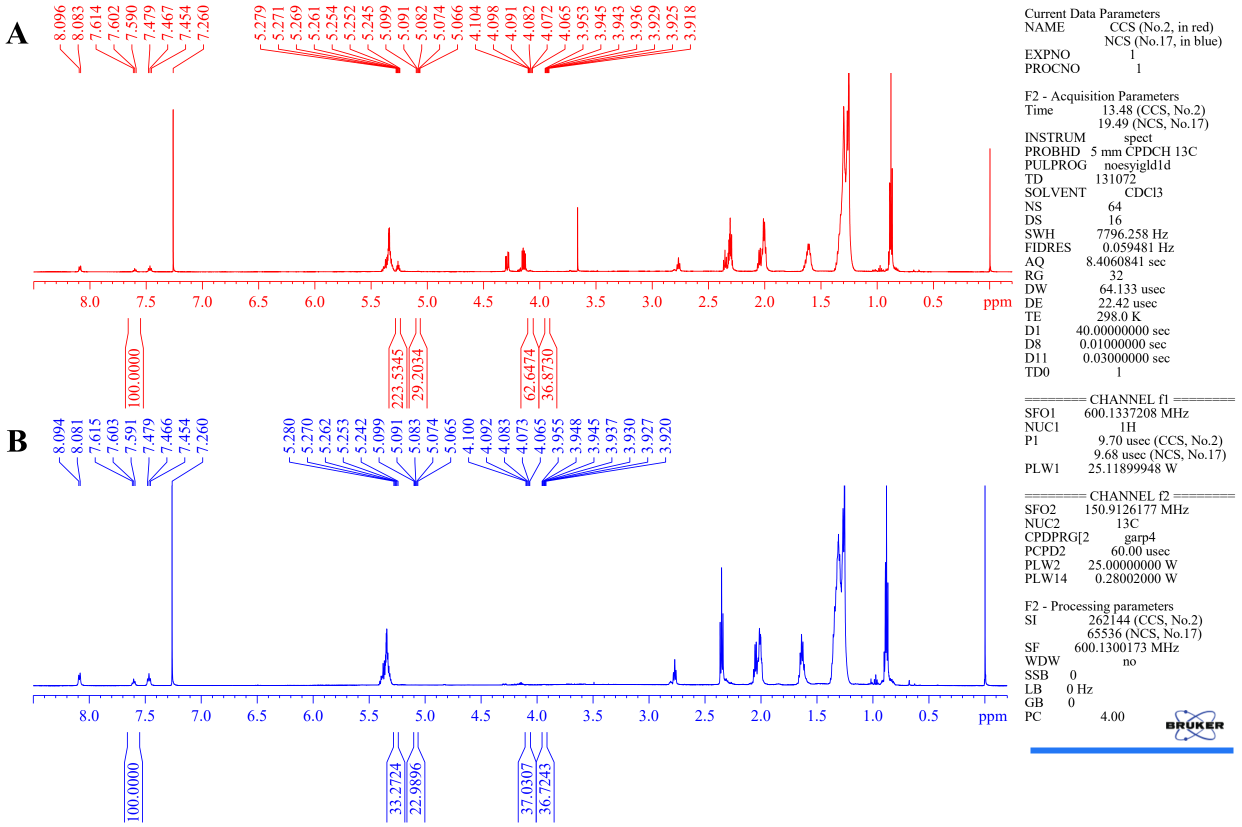

Supplementary Figure S40. The representative ^{1}H-NMR spectra (600 MHz) of *C. sinensis* with internal standard of benzoic acid in $CDCl_3$. (A) The sample of CCS (No.2 in red). (B) The sample of NCS (No.17 in blue).

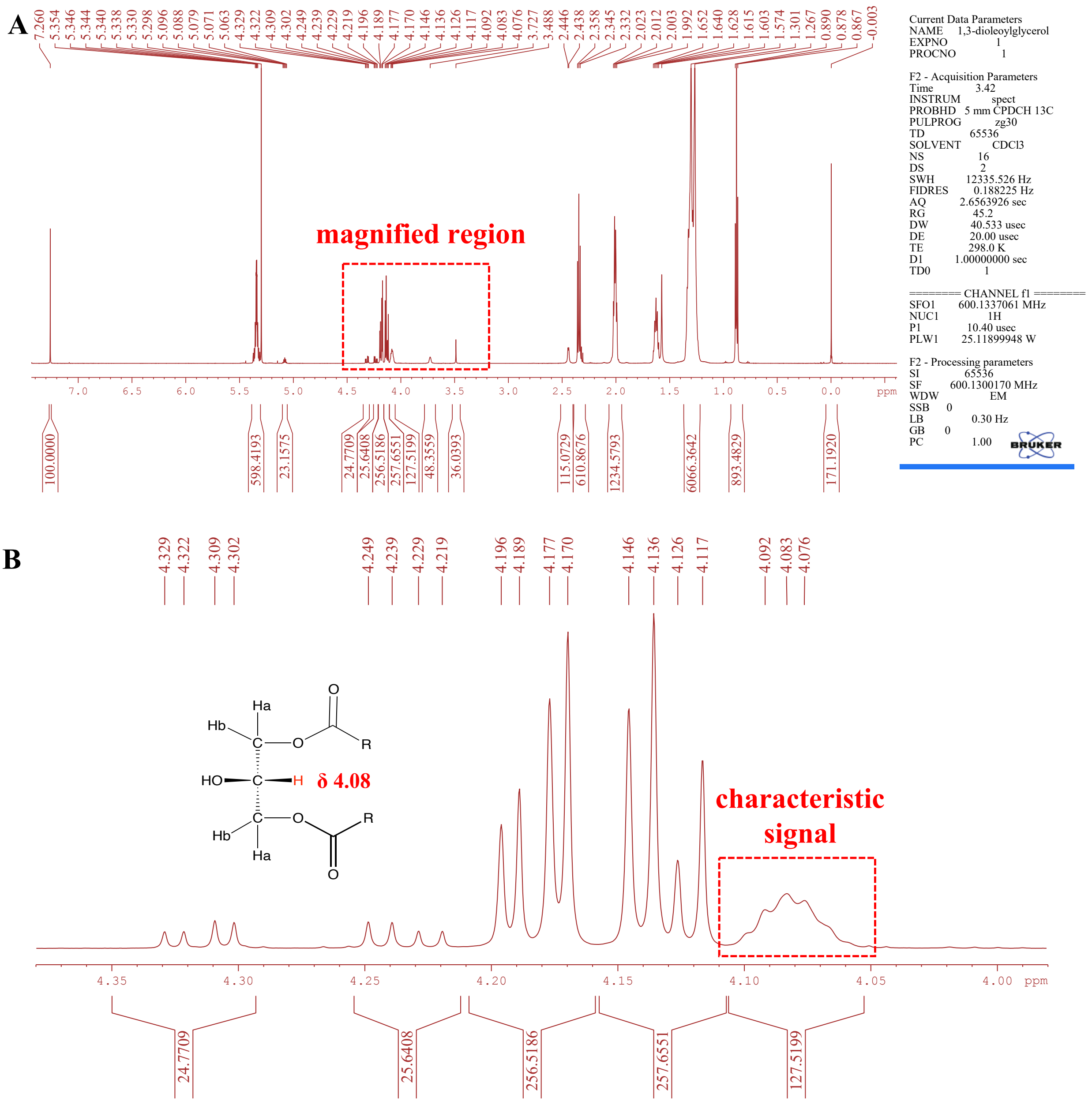

Supplementary Figure S41. The ^{1}H-NMR spectra (600 MHz) of 1,3-dioleoylglycerol standard in $CDCl_3$. (A) The whole ^{1}H-NMR spectrum of 1,3-dioleoylglycerol. (B) The magnified region from δ 4.38 ppm to δ 3.98 ppm, the signal at δ 4.08 ppm was ascribed to the proton at *sn-2* of 1,3-dioleoylglycerol.
